# Conjugation of the Ubiquitin Activating Enzyme UBE1 with the Ubiquitin-Like Modifier FAT10 Targets It for Proteasomal Degradation

**DOI:** 10.1371/journal.pone.0120329

**Published:** 2015-03-13

**Authors:** Johanna Bialas, Marcus Groettrup, Annette Aichem

**Affiliations:** 1 Biotechnology Institute Thurgau at the University of Konstanz, Unterseestrasse 47, CH-8280, Kreuzlingen, Switzerland; 2 Division of Immunology, Department of Biology, University of Konstanz, D-78457, Konstanz, Germany; University of California, Davis, UNITED STATES

## Abstract

The ubiquitin-like modifier HLA-F adjacent transcript 10 (FAT10) directly targets its substrates for proteasomal degradation by becoming covalently attached via its C-terminal diglycine motif to internal lysine residues of its substrate proteins. The conjugation machinery consists of the bispecific E1 activating enzyme Ubiquitin-like modifier activating enzyme 6 (UBA6), the likewise bispecific E2 conjugating enzyme UBA6-specific E2 enzyme 1 (USE1), and possibly E3 ligases. By mass spectrometry analysis the ubiquitin E1 activating enzyme ubiquitin-activating enzyme 1 (UBE1) was identified as putative substrate of FAT10. Here, we confirm that UBE1 and FAT10 form a stable non-reducible conjugate under overexpression as well as under endogenous conditions after induction of endogenous FAT10 expression with proinflammatory cytokines. FAT10ylation of UBE1 depends on the diglycine motif of FAT10. By specifically downregulating FAT10, UBA6 or USE1 with siRNAs, we show that UBE1 modification depends on the FAT10 conjugation pathway. Furthermore, we confirm that UBE1 does not act as a second E1 activating enzyme for FAT10 but that FAT10ylation of UBE1 leads to its proteasomal degradation, implying a putative regulatory role of FAT10 in the ubiquitin conjugation pathway.

## Introduction

The ubiquitin proteasome system (UPS) is one of the major pathways for the regulation of protein homeostasis in human cells. Besides ubiquitin, several related proteins were identified sharing the similar three-dimensional structure, the ubiquitin or β-grasp fold [1]. These ubiquitin-like modifiers (ULM) such as HLA-F adjacent transcript 10 (FAT10), small-ubiquitin-like modifier (SUMO)-1/2/3, ISG15, NEDD8, ATG12, UFM-1 and URM-1 become covalently attached to target proteins via the carboxyl group of their C-terminal glycine residue and form an isopeptide linkage with a lysine residue of the substrate protein [2]. The ubiquitin-like modifier FAT10 (formerly named di-ubiquitin or ubiquitin D) was initially discovered to be encoded in the human Major Histocompatibility Complex (MHC) class I locus [3]. Similar to ubiquitylation also the posttranslational modification with FAT10 was described to directly target proteins for degradation by the 26S proteasome but in case of FAT10 this is performed independently of ubiquitylation [4–6]. FAT10 is an interferon (IFN)γ and tumor necrosis factor (TNF)α inducible protein [7] and it is constitutively expressed in mature dendritic cells [8]. The basal level of FAT10 expression is the highest in organs of the immune system [9–11]. FAT10 overexpression was shown to induce apoptosis in a caspase-dependent manner in mouse fibroblasts, renal tubular epithelial cells and HeLa cells [7,12,13]. It was further shown to play a crucial role in MHC class I-restricted antigen presentation, cell cycle control and chromosomal segregation [14–16]. Additionally, FAT10 is highly upregulated in certain types of cancer [10,11] implying a putative role of FAT10 in the pathogenesis of cancer. The mechanism behind the conjugation of FAT10 to its target proteins is mostly similar to the conjugation pathway of other ubiquitin-like modifiers. Generally, a modifier uses its private cascade consisting of one E1 activating enzyme, one or more E2 conjugating enzymes, and usually several different E3 ligases to become covalently attached to its substrate proteins [17]. In case of FAT10 this is different because the E1 activating enzyme ubiquitin-like modifier activating enzyme 6 (UBA6) as well as the E2 conjugating enzyme UBA6-specific E2 enzyme 1 (USE1) are both bispecific for FAT10 and ubiquitin [18–21]. FAT10ylated proteins are guided to proteasomal degradation and this was e.g. shown for single substrates such as the autophagosomal receptor p62/SQSTM1 or the E2 conjugating enzyme USE1, but also for bulk FAT10 conjugates [22,23]. Next to this function, FAT10 was also shown to interact non-covalently with target proteins such as histone deacetylase 6 (HDAC6) [24], the mitotic spindle checkpoint protein MAD2 [12] or aryl hydrocarbon receptor-interacting protein-like 1 (AIPL1) playing an important role during eye development [25]. In order to identify new interaction partners and substrates of FAT10 we recently performed a large mass spectrometry analysis and showed that endogenous FAT10 becomes conjugated to more than 100 proteins with various functions in several different cell biological processes [23]. One of the identified proteins was the ubiquitin-activating enzyme E1 (UBE1) which we classified as putative substrate of FAT10. UBE1 was first published to solely activate ubiquitin [26,27] but only recently, UBE1 was shown to activate and transfer also the ubiquitin-like modifier NEDD8 under several stress conditions *in cellulo* and *in vitro* [28,29]. Interestingly, an additional function of UBE1 in Atg7- and Atg3-independent autophagy was shown by Chang et al. [30] implying an overlap between the conjugation pathways of different modifiers. In case of UBA6 and USE1, which are both bispecific for ubiquitin and FAT10, the decision which of the modifiers is conjugated to substrate proteins is controlled at least partially on the level of the E1 activating enzyme UBA6. It was shown that UBA6 binds FAT10 with higher affinity than ubiquitin although the adenylation and transthiolation reaction was slower for FAT10 than for ubiquitin [31]. We recently showed that the likewise bispecific E2 conjugating enzyme USE1 undergoes self-FAT10ylation *in cis* which leads to its proteasomal degradation but, interestingly, does not lead to its inactivation concerning FAT10 and ubiquitin transfer [18,22]. We suggested that USE1 auto-FAT10ylation might influence the ability of USE1 to interact with either ubiquitin- or FAT10 specific E3 ligases which would implicate an additional regulatory step for these bispecific conjugation pathways [18,22]. The identification of UBE1 as a putative substrate of FAT10 implies an additional overlap between the two different modifier pathways of FAT10 and ubiquitin. Therefore we were interested to characterize the interaction of UBE1 and FAT10 in more detail. We show here that UBE1 does not act as a second E1 activating enzyme for FAT10 but that it becomes covalently modified by one single FAT10 molecule and this conjugate resists reducing and denaturing conditions. Furthermore we show by specific siRNA-mediated knockdown that FAT10 modification of UBE1 is dependent on the FAT10 specific E1 and E2 enzymes UBA6 and USE1. We suggest that the main function of UBE1-FAT10ylation is to target it to proteasomal degradation which would further favor the FAT10ylation pathway upon the high up-regulation of FAT10 expression during inflammatory processes and imply a regulatory role for FAT10 in the ubiquitin conjugation pathway.

## Results

### UBE1 becomes covalently modified with a single FAT10 molecule

**Fig 1:**
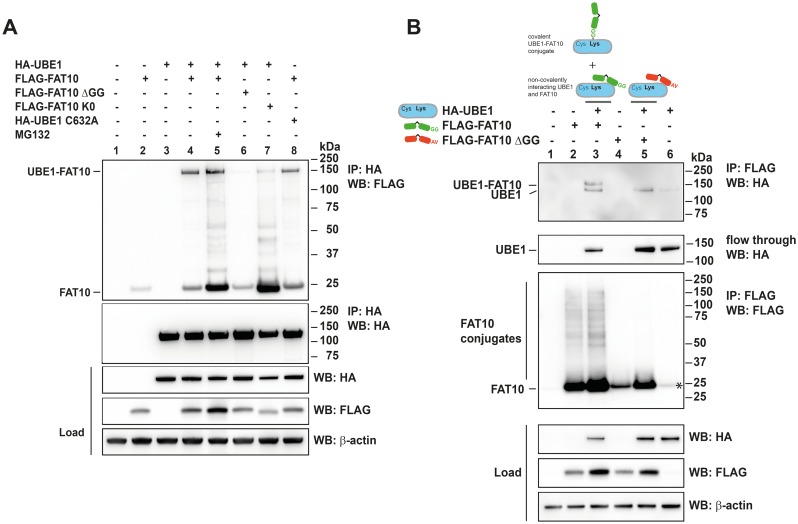
UBE1 is a substrate of FAT10ylation. (A) HEK293 cells were transiently transfected with expression plasmids for HA-UBE1, the active site cysteine mutant of HA-UBE1 (HA-UBE1 C632A), His-3xFLAG-FAT10 (FLAG-FAT10), His-3xFLAG-FAT10 with a mutated diglycine motif at the C terminus (FLAG-FAT10 ΔGG), or a lysine-less mutant of His-3xFLAG-FAT10 (FLAG-FAT10 K0). Where indicated, cells were additionally treated with 10 μM of the proteasome inhibitor MG132 for six hours prior to harvesting. Cell lysates were subjected to immunoprecipitation with anti-HA antibody coupled to agarose. Proteins were separated on 4–12% Bis/Tris NuPAGE gels, and western blot analysis was performed with antibodies reactive against HA or FLAG. β-actin was used as loading control. The upper panels show the immunoprecipitated proteins and the lower western blot panels show the total protein expression in the cell lysates (load). One representative experiment out of three experiments with similar outcomes is shown. (B) HEK293 cells were transfected with the expression constructs indicated, harvested and lysed as described in (A). Cell lysates were subjected to immunoprecipitation with anti-FLAG antibody coupled to agarose and subsequent western blots were performed as described in (A). An asterisk in lane 6 marks an unspecific background band in the WB against FLAG. Cartoons describe the covalent and non-covalent interactions of UBE1 and FAT10. One representative experiment out of three experiments with similar outcomes is shown.

Recently we published proteomic data to identify new interaction partners and substrates of endogenous FAT10. In this analysis, the ubiquitin-activating enzyme UBE1 was identified and classified as a novel putative substrate of FAT10 [23]. In order to confirm this classification and to show that UBE1 and FAT10 indeed interact and form a stable conjugate, co-immunoprecipitations of HA-tagged UBE1 (HA-UBE1) and different versions of FLAG-tagged FAT10 (FLAG-FAT10) transiently overexpressed in HEK293 cells were performed and analyzed under reducing conditions by western blot analysis (Fig. 1A). Upon expression of HA-UBE1 and FLAG-FAT10 a non-reducible conjugate with an apparent molecular mass of approximately 150 kDa was formed (Fig. 1A, upper panel, lane 4) and the amount of the conjugate increased upon proteasome inhibition with MG132 (Fig. 1A, lane 5), suggesting that FAT10ylated UBE1 may be guided to proteasomal degradation. When HA-UBE1 was co-expressed with a FLAG-FAT10 mutant lacking the C-terminal diglycine motif (FLAG-FAT1ΔGG) only a minor portion of FLAG-FAT10ΔGG formed a conjugate with HA-UBE1, most probably due to the high protein concentrations under overexpression conditions (Fig. 1A, lane 6), further supporting the conception of a covalent attachment of FAT10 to UBE1 via its C-terminal diglycine motif. Additionally, the overexpression of a lysine-less FAT10 mutant (FLAG-FAT10 K0) revealed a conjugate with the same size as the wildtype HA-UBE1-FLAG-FAT10 conjugate, indicating that UBE1 becomes modified by one single FAT10 molecule (Fig. 1A, lane 7). Monomeric FAT10 was also immunoprecipitated together with HA-UBE1, pointing to an additional non-covalent interaction of the two proteins (Fig. 1A, lanes 4–8). To further characterize the non-covalent interaction of FAT10 and UBE1, an immunoprecipitation of FLAG-tagged FAT10 or its diglycine mutant FLAG-FAT10ΔGG was performed and the non-covalent interaction of HA-UBE1 was investigated by a subsequent western blot analysis using an HA-reactive antibody (Fig. 1B). As shown in Fig. 1B, both, the HA-UBE1-FLAG-FAT10 conjugate as well as unconjugated HA-UBE1 were immunoprecipitated with FLAG-FAT10 (Fig. 1B, lane 3). HA-UBE1 was also immunoprecipitated with the FAT10 diglycine mutant FLAG-FAT10ΔGG (Fig. 1B, lane 5), indicating a non-covalent interaction of UBE1 and FAT10 which might be independent of the FAT10 diglycine motif. To verify the formation of a stable non-reducible isopeptide linkage between UBE1 and FAT10, an immunoprecipitation of overexpressed proteins under denaturing conditions was performed. A subsequent western blot analysis under reducing conditions revealed that the UBE1-FAT10 conjugate was still detectable confirming that UBE1 and FAT10 formed a stable isopeptide linkage (S1 Fig.).

**Fig 2:**
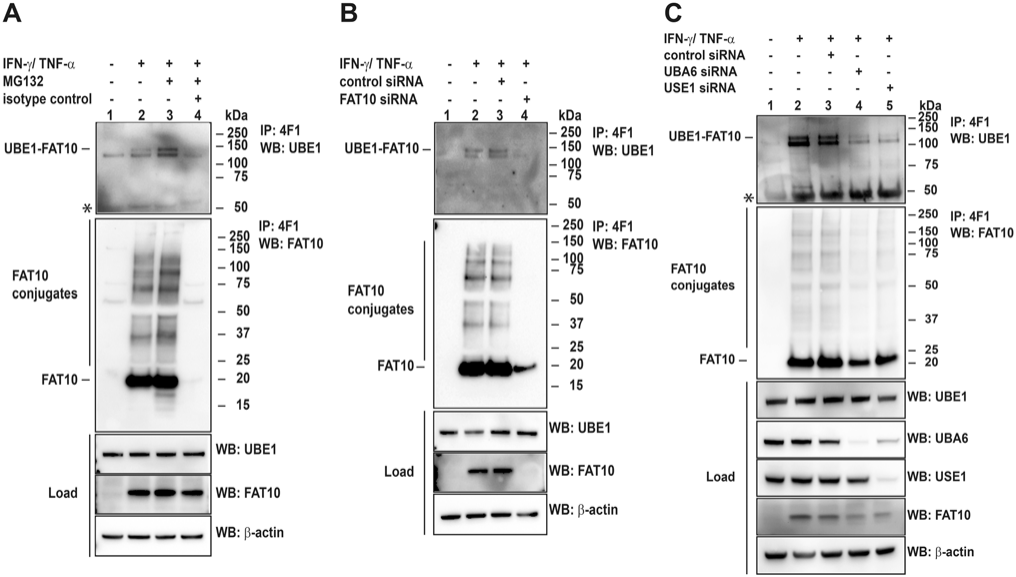
Endogenous UBE1-FAT10 conjugate formation is dependent on UBA6 and USE1. (A) Total cell extracts from IFNγ and TNFα-stimulated HEK293 cells were used to immunoprecipitate endogenous FAT10 and the UBE1-FAT10 conjugate with a mAb against FAT10 (4F1) or an unspecific IgG-agarose as control, followed by western blot analysis using polyclonal antibodies against FAT10 and UBE1. Before harvesting, cells were treated with proteasome inhibitor MG132 for 6 hours, where indicated. Proteins were separated under reducing conditions (4% 2-mercaptoethanol) on 4–12% Bis/Tris NuPAGE gels. The upper panel shows the immunoprecipitated UBE1-FAT10 conjugate, the middle panel the immunoprecipitated FAT10 conjugates, the lower western blot panels show protein expression levels in total cell lysates (load). β-actin was used as loading control. An asterisk indicates the heavy chain of the antibody used for immunoprecipitation. One representative experiment out of three experiments with similar outcomes is shown. (B) HEK293 cells were treated with IFNγ/TNFα to stimulate endogenous FAT10 expression as described in (A). Additionally, cells were treated on two days either with control siRNA or FAT10-specific siRNA to downregulate endogenous FAT10 expression at the same time as it was induced. Cells were harvested on day three, and cell lysates were subjected to immunoprecipitation of endogenous FAT10 and the UBE1-FAT10 conjugate with a mAb against FAT10 (4F1). Proteins were separated on 4–12% NuPAGE gels and western blot analysis was performed under reducing conditions (4% 2-mercaptoethanol) with polyclonal antibodies against FAT10 and UBE1. β-actin was used as loading control. The upper western blot panel shows the disappearance of the UBE1-FAT10 conjugate after siRNA treatment, the middle panel shows immunoprecipitated FAT10 conjugates, and the lower panels show protein expression levels in total protein lysates (load). One representative experiment out of three experiments with similar outcomes is shown. (C) Same experimental setup as in (B) only that UBA6 and USE1 were specifically knocked down by treating HEK293 cells with specific siRNAs against UBA6 and USE1, respectively. Control cells were either untreated or treated with unspecific control siRNA. An asterisk indicates the heavy chain of the antibody used for immunoprecipitation. One representative experiment out of three experiments with similar outcomes is shown.

To verify that the UBE1-FAT10 conjugate formation was not due to overexpression of the two proteins but was formed also under endogenous conditions, HEK293 cells were treated for 24 hours with the proinflammatory cytokines interferon (IFN)γ and tumor necrosis factor (TNF)α to induce endogenous FAT10 expression. To visualize the endogenous UBE1-FAT10 conjugate, endogenous FAT10 was immunoprecipitated with a FAT10-reactive monoclonal antibody followed by western blot analysis with an antibody specific for endogenous UBE1. As shown in Fig. 2A, the UBE1-FAT10 conjugate was formed also under endogenous conditions, it appeared as a double band and was absent when the unspecific isotype control instead of the FAT10-reactive antibody was used for immunoprecipitation (Fig. 2A, lane 4). Mass spectrometry analysis of the two bands revealed that the upper band contained the UBE1-FAT10 conjugate because both proteins were identified at a molecular mass, corresponding to the UBE1-FAT10 conjugate (S1 Table, sample S2). As a control, by analysis of corresponding gel slices deriving from unstimulated cells and therefore from cells, not expressing FAT10, no UBE1 or FAT10 was identified (S1 Table, sample C2). UBE1 and FAT10 were both also identified in the lower band deriving from cytokine stimulated cells (S1 Table, sample S1), but UBE1 was also identified in the respective unstimulated control (S1 Table, sample C1). Therefore we suggest that a portion of UBE1 unspecifically interacted with the protein A sepharose used for immunoprecipitation of FAT10. However, since in most cases the amount of UBE1 in the lower band increased when FAT10 was expressed and immunoprecipitated and since at the same time the amount of UBE1 in the lysate (load) remained stable, it might also represent UBE1, non-covalently interacting with FAT10 as already shown in Fig. 1B in case of HA-UBE1 and FLAG-FAT10. As shown for the conjugate formed under overexpression conditions, the endogenous UBE1-FAT10 conjugate accumulated when cells were treated for six hours with the proteasome inhibitor MG132 (Fig. 2A, lanes 2 and 3). Modification of substrate proteins with FAT10 has previously been shown to depend on the activation of FAT10 by the E1 activating enzyme UBA6, and its transfer by the E2 conjugating enzyme USE1 [18]. To investigate whether this is also necessary for the formation of the UBE1-FAT10 conjugate, we used siRNAs to down-regulate FAT10, UBA6 or USE1 upon induction of FAT10 expression with cytokines. As expected, upon knockdown of endogenous FAT10 no monomeric FAT10 and no FAT10 conjugates were detectable anymore. In addition, the amount of the UBE1-FAT10 conjugate was clearly reduced, further supporting the UBE1-FAT10 conjugate formation under endogenous conditions (Fig. 2B, lane 4). A similar result was obtained after knockdown of UBA6 and USE1 mRNAs by about 85% which also strongly abrogated the formation of the endogenous UBE1-FAT10 conjugate (Fig. 2C, lanes 4 and 5). Taken together, the results obtained so far show that UBE1 is a novel substrate of FAT10 and that UBE1-FAT10ylation is dependent on a functional FAT10 conjugation pathway.

### UBE1 does not act as a second E1 enzyme for FAT10

**Fig 3:**
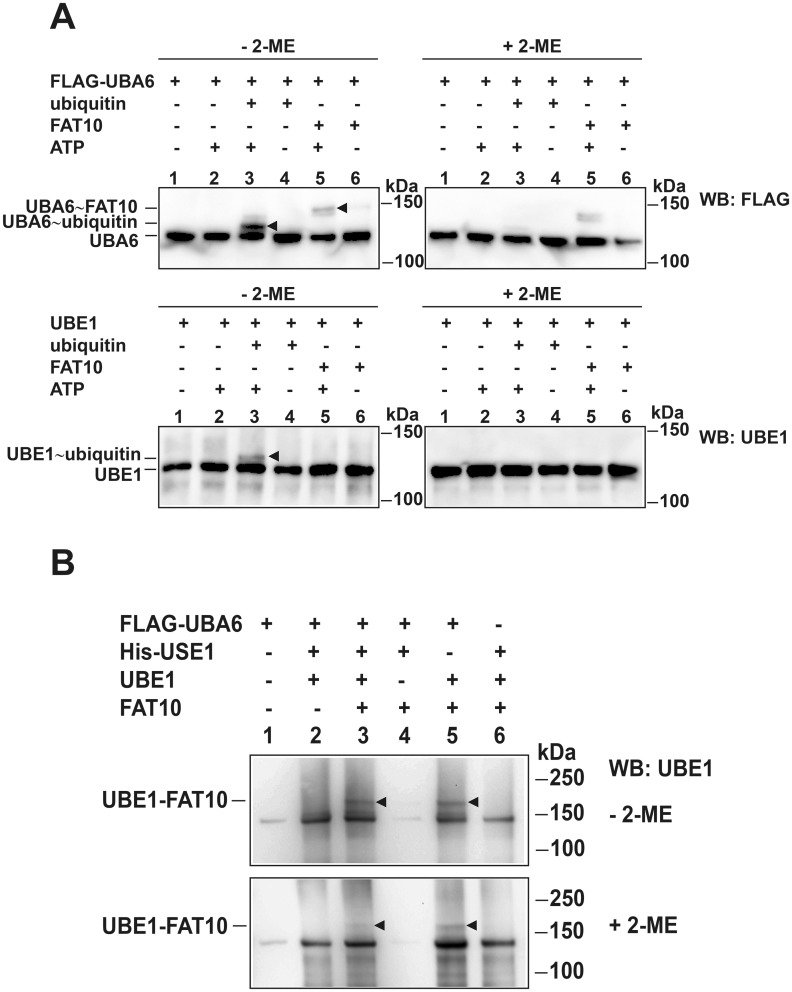
UBE1 is not a second E1 activating enzyme for FAT10, but a substrate of FAT10ylation *in vitro*. Recombinant proteins were incubated in reaction buffer at 30°C for 60 min and separated on 3–8% gradient Tris/Acetate NuPAGE SDS gels. Western blot analysis was performed under non-reducing (-2-mercaptoethanol (2-ME), left panels) or reducing (+ 2-ME (4%), right panels) conditions by using an antibody against FLAG to visualize FLAG-UBA6 or a polyclonal antibody reactive to UBE1. (A) Upper western blot panels show FLAG-UBA6 thioester-linked ubiquitin and FAT10, respectively (arrow heads in lane 3 and 5). Lower panels show ubiquitin activation by UBE1 (arrow head in lane 3), but no activation of FAT10 by UBE1. One experiment out of three experiments with similar outcomes is shown. (B) Western blot showing the *in vitro* conjugation of recombinant FAT10 onto UBE1 in presence of UBA6 and/or USE1 under reducing conditions (4% 2-ME). Arrow heads indicate the UBE1-FAT10 conjugate in lane 3 and 5. One representative experiment out of four experiments with similar outcomes is shown.

For a long time, UBE1 was thought to solely activate ubiquitin [26,27] but more recently it has emerged that UBE1 additionally activates and transfers NEDD8 under specific stress conditions [28,29]. Therefore we were interested to investigate whether UBE1 would in addition serve as a second E1 activating enzyme for FAT10. To address this question the active site cysteine of UBE1 was mutated to an alanine (HA-UBE1 C632A) and the mutant UBE1 was co-expressed together with FLAG-tagged FAT10 in HEK293 cells followed by co-immunoprecipitation and western blot analysis under reducing conditions. Independent of a functional active site cysteine the UBE1-FAT10 conjugate was still formed (Fig. 1A, lane 8), showing that the formation of the UBE1-FAT10 conjugate was not dependent on the preceding transfer of FAT10 onto the active site cysteine of UBE1, as it is e.g. shown in case of auto-FAT10ylation of USE1 [18]. To corroborate that UBE1 did not act as a second E1 activating enzyme of FAT10, UBE1 was recombinantly expressed and purified from *E*. *coli*, and subjected to *in vitro* FAT10- or ubiquitin-activation assays. The respective proteins were incubated at 30°C for 60 min and western blot analysis was performed under non-reducing and reducing (4% 2-mercaptoethanol, 2-ME) conditions to monitor thioester bond formation. Recombinant FLAG-UBA6 was used as positive control for FAT10 activation. As shown in Fig. 3A, both, ubiquitin and FAT10 were activated by FLAG-UBA6 and the thioester bonds were almost completely reducible in presence of 4% 2-mercaptoethanol (+2-ME) (Fig. 3A, upper panels, arrow heads in lanes 3 and 5). In comparison to this, recombinant UBE1 and ubiquitin formed a thioester bond which was completely absent under thioester-reducing conditions (Fig. 3A lower panels, arrow head in lane 3). In contrast, no activation of recombinant FAT10 by UBE1 was detectable under non-reducing conditions (Fig. 3A lower panels, lanes 5) showing that UBE1 does not act as a second E1 activating enzyme for FAT10.

### Recombinant FAT10 becomes covalently attached to UBE1 *in vitro*

To investigate FAT10ylation of UBE1 also under *in vitro* conditions, recombinant FLAG-UBA6, 6His-tagged USE1, UBE1 and FAT10 were incubated for one hour at 30°C in the presence of ATP. Samples were subjected to western blot analysis under non-reducing and reducing conditions. As shown in Fig. 3B (arrow heads in lane 3), UBE1 was FAT10ylated by the conjugation machinery consisting of UBA6 and USE1 but without the help of an E3 ligase as it is also described for some SUMO substrates where an E3 ligase is not necessarily needed to transfer SUMO onto a substrate [32]. Similarly, FAT10ylation of UBE1 was also detected in the absence of the E2 conjugating enzyme USE1 (Fig. 3B, arrow heads in lane 5), indicating that under the *in vitro* conditions used in this setup the activation by UBA6 was sufficient to transfer FAT10 onto its substrate UBE1. The recombinant 6His-USE1 used in this assay was functional as it could be charged with recombinant FAT10 or 6His-ubiquitin by FLAG-UBA6 under non-reducing conditions (S2 Fig.) which further confirmed that the presence of the E1 activating enzyme UBA6 was sufficient for *in vitro* FAT10ylation of UBE1.

### FAT10 targets UBE1 for proteasomal degradation

**Fig 4:**
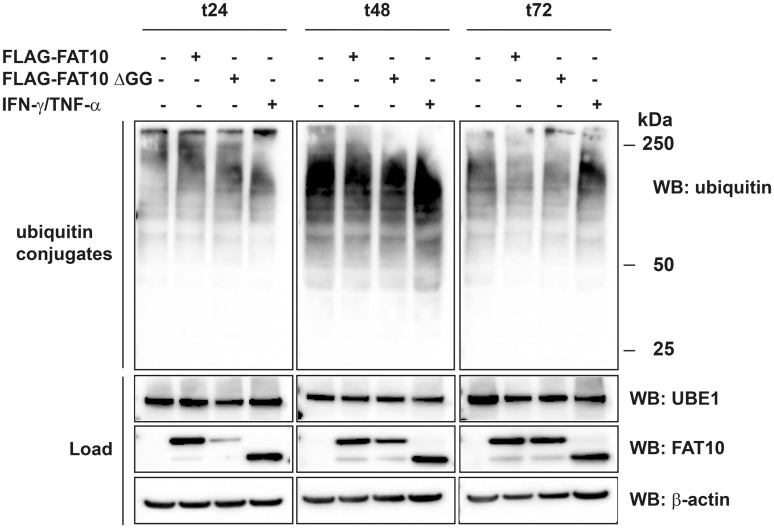
FAT10 expression does not influence the formation of bulk ubiquitin conjugates. HEK293 cells were transiently transfected with expression plasmids for His-3xFLAG-FAT10 (FLAG-FAT10) or a His-3xFLAG-FAT10 mutant which has a GG-to-AV mutation at the C terminus (FLAG-FAT10 ΔGG) which renders it unable to be conjugated to substrate proteins. Alternatively, endogenous FAT10 expression was induced by treatment with proinflammatory cytokines IFNγ and TNFα. To maintain high FAT10 levels over time, cells were re-transfected or re-stimulated after 48 h. Cells were harvested after indicated time periods of expression and total lysates were subjected to protein separation on 4–12% Bis/Tris NuPAGE gels, followed by western blot analysis with specific antibodies against FLAG, endogenous ubiquitin or UBE1. The upper panels show bulk ubiquitin conjugates, and the lower panels show the expression levels of endogenous UBE1 and overexpressed or endogenous FAT10 in the cell lysates (load). β-actin was used as loading control. One representative experiment out of four experiments with similar outcomes is shown.

**Fig 5:**
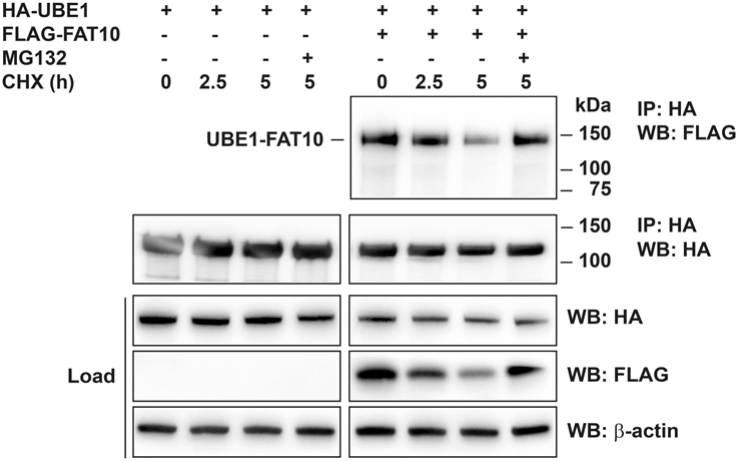
FAT10ylated UBE1 is degraded by the proteasome. HEK293 cells were transiently transfected with expression plasmids for HA-UBE1 with or without His-3xFLAG-FAT10 (FLAG-FAT10), as indicated. Cell lysates were used for immunoprecipitation with anti-HA-agarose followed by western blot analysis with antibodies reactive against HA or FLAG. Cells were additionally treated for the indicated time periods with cycloheximide (CHX) and/or the proteasome inhibitor MG132, as indicated. Proteins were separated under reducing conditions (4% 2-ME) on 4–12% NuPAGE gels. The upper panels show immunoprecipitated proteins, the lower panels show the protein expression in total cell lysates (load). β-actin was used as loading control. One representative experiment out of four experiments with similar outcomes is shown.

To obtain information about the influence of FAT10 modification on the activity of UBE1 we addressed the question whether FAT10ylation of UBE1 would have an influence on bulk ubiquitin conjugate formation in cells. To this aim, HEK293 cells were transiently transfected with an expression plasmid for FLAG-FAT10, or endogenous FAT10 expression was induced by treating the cells with IFNγ and TNFα for up to 72 hours. FLAG-FAT10 lacking the C-terminal diglycine motif (FLAG-FAT10ΔGG) served as negative control since it is not covalently conjugated to UBE1 and therefore should not have an influence on the activity of UBE1. Cells were harvested after 24, 48 and 72 hours and cell lysates were subjected to western blot analysis with a ubiquitin-specific antibody. As shown in Fig. 4, the presence of either overexpressed or induced FAT10 had no influence on the level of bulk ubiquitin conjugates. This result may be attributed to the fact that only a small amount of UBE1 becomes modified with FAT10 while the main portion of UBE1 remains unmodified. This might render it difficult to detect differences in bulk ubiquitin conjugate formation. In most cases, covalent modification of proteins with FAT10 leads to their proteasomal degradation as e.g. shown for the FAT10 substrates p62/SQSTM1 or USE1 [22,23]. Therefore it was pertinent to investigate whether FAT10ylation of UBE1 would also guide UBE1 to proteasomal degradation. To monitor the degradation of FAT10ylated UBE1, cells were treated for 2.5 or 5 hours with cycloheximide (CHX) to block *de novo* protein synthesis. As shown in Fig. 5 (upper right panel) and S3 Fig., the UBE1-FAT10 conjugate was degraded within five hours by about 40% (see quantification of ECL signals in S3 Fig.) and degradation was rescued when cells were treated at the same time with proteasome inhibitors MG132, bortezomib or lactacystin, confirming the results shown in Fig. 1A where the HA-UBE1-FLAG-FAT10 conjugate also accumulated upon MG132 treatment. At the same time, unconjugated UBE1 in the presence or absence of FAT10 remained stable and did not accumulate when the proteasome was inhibited (Fig. 5, load WB: HA and IP: HA, WB: HA). Moreover we have previously shown an accumulation of the HA-UBE1-FLAG-FAT10 conjugate upon specific knockdown of the FAT10 proteasome receptor Rpn10 [33]. Taken together, our data confirm that UBE1 is a novel substrate of FAT10 conjugation, and that a consequence of UBE1 FAT10ylation is to target UBE1 for proteasomal degradation.

## Discussion

A recently performed large scale mass spectrometry analysis has identified the ubiquitin-activating enzyme E1 (UBE1) as a novel putative FAT10-interacting protein and FAT10ylation substrate [23]. In the current study we verify the interaction between UBE1 and FAT10 and we confirm that UBE1 becomes FAT10ylated upon overexpression or induction of endogenous FAT10 expression (Figs. 1 and 2). The UBE1-FAT10 conjugate formation is dependent on the presence of the FAT10 E1 activating and E2 conjugating enzyme, UBA6 and USE1, respectively, as well as on the FAT10 di-glycine motif. Nevertheless, in some of the experiments a very faint UBE1-FAT10ΔGG conjugate was sometimes detected upon co-expression of HA-UBE1 and FLAG-FAT10ΔGG and subsequent immunoprecipitation of HA-UBE1 (Fig. 1A, lane 6). However, when the immunoprecipitation was performed in the reverse direction and FLAG-FAT10ΔGG was immunoprecipitated, no such conjugate was detectable (Fig. 1B, lane 5), pointing to an unspecific conjugate formation due to the high protein content under overexpression conditions. Interestingly, the induction of FAT10 expression with proinflammatory cytokines IFNγ and TNFα led to the formation of a double UBE1-FAT10 conjugate band. Mass spectrometry analysis revealed that the upper band contained UBE1 and FAT10. The lower band consisted most probably of non-covalently FAT10-interacting UBE1 because its amount also increased when FAT10 accumulated due to proteasome inhibition (Fig. 2). This finding was further supported by our data showing a non-covalent interaction of HA-UBE1 and FLAG-FAT10 upon immunoprecipitation of FLAG-FAT10 (Fig. 1B). Also under these conditions, both, UBE1 as well as the UBE1-FAT10 conjugate were detected upon western blot analysis using a UBE1-reactive antibody (Fig. 1B). Consistent with other FAT10 substrates, such as p53 or USE1 [18,34], no further conjugates with a higher molecular weight could be detected under overexpression conditions, excluding a poly-FAT10ylation or a multi-mono-FAT10ylation of UBE1, as it was shown for the FAT10 substrate p62 [23] or LRRFIP2 [35]. Overexpression of the lysine-less FAT10 mutant confirmed the results of a mono-FAT10ylation of UBE1 (Fig. 1A). We further investigated the question whether UBE1 could act as second E1 activating enzyme for FAT10. So far, UBA6 was known as to be the sole E1 activating enzyme for FAT10 [19,21], and UBE1 was shown to activate both, ubiquitin and NEDD8 under different stress conditions [26–29]. The overexpression of an active site cysteine mutant of UBE1 (HA-UBE1 C632A) revealed a covalent modification of UBE1 with FAT10 independent of the active site cysteine and thereby without the need of a preceding thioester formation via the active site cysteine (Fig. 1A, lane 8) as it was shown in case of auto-FAT10ylation of the E2 conjugating enzyme USE1 [18]. In case of USE1, mutation of the active site cysteine to an alanine (USE1-C188A) completely abolished USE1 auto-FAT10ylation showing that first FAT10 is transferred from UBA6 onto the active site cysteine of USE1 and then in a second step to an internal lysine residue of USE1 to form the stable conjugate. In *in vitro* experiments with recombinant proteins we showed that UBA6 activated both ubiquitin and FAT10, which is in agreement with previous findings from different groups [19–21] but that UBE1 was only able to activate ubiquitin but not FAT10 clearly showing that UBE1 does not act as second E1 activating enzyme for FAT10 (Fig. 3A). Additionally, we showed that UBE1 becomes FAT10ylated *in vitro* (Fig. 3B). Strikingly, the activation of FAT10 by UBA6 was sufficient to transfer FAT10 onto UBE1 *in vitro*. This finding was quite remarkable, because the E2 conjugating enzymes normally mediate the close proximity and specificity of modifier, E3 ligase and substrate protein [36]. In line with this, the presence of the E2 enzyme USE1—in spite of being active, as shown in loading experiments with FAT10 and ubiquitin—did not further increase the amount of the UBE1-FAT10 conjugate, maybe because of the already high protein amount in the reaction and thereby close proximity of the proteins. *In vitro* transfer experiments without the need of a specific E3 ligase were also published for the ubiquitin-like modifier SUMO, where it was also sufficient just to include the SUMO-specific E1 and E2 enzymes to obtain SUMOylated substrates [32,37]. However, we cannot exclude that under *in vivo* conditions, where the proteins are present in much lower concentrations than in our *in vitro* assays, the activity of a specific E3 ligase is indispensable to perform the FAT10 conjugation to substrate proteins.

To investigate the functional consequences of the FAT10ylation of UBE1, we looked deeper into the available crystal structures of UBE1 because we were interested to know, if a FAT10 modification could have an influence on the ubiquitin-activating function of UBE1. The overlay of mouse and yeast UBE1/Uba1 crystal structures [38,39] revealed four lysine residues around the active site cysteine of UBE1 (C632) which are conserved between the two species. Three of these lysine residues (K635, K801, K805) are localized in a cave directly located under the active site cysteine which is exposed in a well. Modification of one of these lysine residues could thereby theoretically inhibit the formation of the ubiquitin thioester linkage of ubiquitin and the UBE1 active site cysteine by changing the conformation of the area around the active site cysteine maybe by sliding into this cave. The fourth lysine residue (K745) is located next to the active site cysteine. We speculated that FAT10ylation of K745 could also block the binding of ubiquitin to C632 by steric inhibition because FAT10 could theoretically cover the cysteine residue due to its size. To test these ideas, site-directed mutagenesis of different combinations of these residues were performed and lysines were mutated to arginines. To show that mutations of lysines around such an important region of the protein do not impair ubiquitin activation by UBE1, the formation of the UBE1-ubiquitin thioester was successfully confirmed in HEK293 cells, transiently expressing the mutant HA-UBE1 variants together with FLAG-ubiquitin. However, all mutants became FAT10ylated in amounts comparable to wildtype UBE1 (data not shown). These data suggest that either the mutated lysine residues are not the major FAT10ylation sites within UBE1, or that FAT10 modification switches to another lysine upon mutation of the main lysine as it was recently shown for the auto-FAT10ylation of USE1 [22] or auto-SUMOylation of the SUMO E2 conjugating enzyme Ubc9 [40]. To further address this issue, the UBE1-FAT10 conjugate will be analyzed by mass spectrometry analysis in future experiments.

As shown for other substrates which become modified with ubiquitin-like modifiers, such as p53 by SUMO or USE1 and p62 by FAT10 [18,23,41], only a small portion of UBE1 appears to become modified with FAT10, which makes the search for functional consequences even more challenging. Since it was published for several different FAT10 substrates, such as p62 and USE1 [22,23] that FAT10ylation targets substrate proteins for proteasomal degradation, we investigated whether UBE1 is also guided to degradation by the proteasome upon conjugation with FAT10 and we show that overexpressed UBE1-FAT10 conjugate amounts increase upon inhibition of the proteasome by treatment with MG132 (Fig. 1). Additional cycloheximide chase data showed that the UBE1-FAT10 conjugate is also degraded by about 40% within a time period of five hours (Fig. 5 and S3 Fig.). Blockage of the proteasome with MG132, bortezomib or lactacystin rescued the UBE1-FAT10 conjugate from degradation (Fig. 5 and S3 Fig.). These data confirmed a previous publication of our group where we knocked down Rpn10, the docking site of FAT10 at the proteasome, and where we could show an accumulation of the UBE1-FAT10 conjugate upon this knockdown [33]. To investigate if FAT10ylation of UBE1 might have additional functional consequences besides degradation by the proteasome, we performed several additional experiments. The up-regulation of FAT10 expression by overexpression or treatment with proinflammatory cytokines did not influence the bulk UBE1 protein level in cells. We previously obtained the same results for the E2 conjugating enzyme USE1 [22]. Up-regulation of FAT10 led also here to the modification of only a small amount of USE1 but also did not alter the amount of the unconjugated USE1. We suggest that this is true in case of UBE1 as well and that this prevents the identification of functional consequences when at the same time most of the protein remains unmodified and active. Nevertheless we tested whether FAT10ylation of UBE1 has an impact on bulk ubiquitin conjugate formation, maybe due to a functional impairment of FAT10ylated UBE1 or because of slightly reduced UBE1 levels due to degradation of the UBE1-FAT10 conjugate. However, we detected no decrease of the bulk ubiquitin conjugates upon overexpression of FAT10 or upon induction of endogenous FAT10 expression with cytokines (Fig. 4). The expression of both, FAT10 and the E2 conjugating enzyme USE1 seems to be regulated during the cell cycle [42,43]. In addition, UBE1 is mainly found in the nucleus [44–46] whereas FAT10 seems to be more abundant in the cytoplasm [24]. All experiments in the current study were performed with unsynchronized cells. We therefore wondered if FAT10ylation of UBE1 might have a specific function during the cell cycle and investigated if the formation of the UBE1-FAT10 conjugate might happen specifically during a certain phase of the cell cycle such as e.g. mitosis, where due to the breakdown of the nuclear membrane both proteins would come in close proximity. However, our preliminary results provided no evidence for a cell cycle regulated formation of the UBE1-FAT10 conjugate (data not shown). Other functional consequences of UBE1 FAT10ylation might be that FAT10ylation could change the cellular localization of UBE1 by e.g. masking the nuclear localization sequence of UBE1 or that FAT10ylation induces conformational changes within UBE1. Changes in the localization or conformation of UBE1 could in turn result in a gain of function such as e.g. altering the set of UBE1 interacting E2 conjugating enzymes.

Taken all results together, our data provide evidence, that UBE1 is a novel substrate of the FAT10 conjugation machinery and that it does not serve as a second FAT10 E1 activating enzyme next to UBA6. We clearly show that the activation by UBA6 is sufficient to activate and transfer FAT10 onto UBE1 *in vitro*, and that FAT10ylation of UBE1 leads to degradation of the UBE1-FAT10 conjugate by the proteasome.

## Materials and Methods

### Primers and constructs

Plasmids used for transient transfection of HEK293 cells with TransIT-LTI Transfection Reagent (Mirus Bio LLC, Madison, WI, USA) were pcDNA3.1-His-3xFLAG-FAT10 for expression of FLAG-tagged FAT10 [19], pcDNA3.1-His-3xFLAG-FAT10ΔGG for expression of FLAG-FAT10 lacking the C-terminal diglycine motif [23], pcDNA3.1-His-3xFLAG-FAT10 K0 for expression of a FLAG-tagged lysine-less mutant of FAT10 (A. Aichem, unpublished) and pcDNA3.1-His-3xFLAG-ubiquitin for FLAG-ubiquitin expression (M. Basler, unpublished). For the expression of HA-tagged UBE1 pcDNA3.1-HA-UBE1, kindly provided by Dr. M. Scheffner (University of Konstanz, Germany), was used. For the generation of the active site cysteine to alanine mutant of HA-UBE1 (HA-UBE1-C632A), the QuikChange II Site-directed Mutagenesis Kit (Agilent Technologies, Santa Clara, CA, USA) with pcDNA3.1-HA-UBE1 as template and the following primer was used: 5’CCTGAGAAGTCCATCC CCATCGCTACCCTGAAGAACTTCCCTAATGC-3’. All plasmids were verified by sequencing (Microsynth AG, Balgach, Switzerland).

### Induction of endogenous FAT10 expression, CHX chase experiments, and immunoprecipitation

Induction of endogenous FAT10 expression by the proinflammatory cytokines IFNγ (200 U·mL^-1^) and TNFα (400 U·mL^-1^) (both from Peprotech GmbH, Hamburg, Germany) was performed as recently described [23,47]. Before harvesting, 10 μM of the proteasome inhibitor MG132 (Enzo Lifesciences, Lausen, Switzerland) was added as indicated, and the cells were incubated for additional 6 hours. Where indicated, cells were additionally treated for 5 hours with 50 μg·mL^-1^ cycloheximide (CHX) (Sigma-Aldrich, Buchs, Switzerland) for the indicated time periods in parallel with 10 μM MG132, 10 μM lactacystin (both from Enzo Lifesciences, Lausen, Switzerland) or 1 μM bortezomib (Millenium Pharmaceuticals, Boston, USA) treatment for 6 h. Cells were harvested and lysed for 30 min on ice in lysis buffer containing 20 mM Tris/HCL (pH 7.6), 50 mM NaCl, 10 mM MgCl_2_, and 1% Nonidet P-40, supplemented with 1x protease inhibitor mix (complete, mini, EDTA-free protease inhibitor cocktail; Roche, Rotkreuz, Switzerland). Cleared lysates were subjected to immunoprecipitation using a mouse mAb against FAT10 (clone 4F1) bound to protein A sepharose, as recently described [18,47], or a mouse IgG-agarose as isotype control (Sigma-Aldrich, Buchs, Switzerland). Proteins were separated on 4–12% gradient Bis/Tris NuPAGE SDS gels (Invitrogen, Lucerne, Switzerland), and subjected to western blot analysis with either a rabbit polyclonal antibody against huFAT10, which is crossreactive with moFAT10 [4], or a rabbit pAb against UBE1 (Enzo Lifesciences, Lausen, Switzerland). An antibody against β-actin (Abcam, Cambridge, UK) was used as a loading control. Immunoprecipitation of overexpressed HA-tagged proteins was performed with anti-HA-agarose conjugate HA-7 (Sigma Aldrich, Buchs, Switzerland). Proteins were detected with directly labeled peroxidase-conjugated mAb against HA-7 (Sigma Aldrich, Buchs, Switzerland).

### siRNA-mediated knockdown

As previously described [18], HEK293 cells were transfected twice with a pool of four different UBA6 specific siRNAs (Hs_UBE1L2_1, Hs_UBE1L2_2, Hs_FLJ10808_2, Hs_FLJ10808_4), a pool of four different USE1 specific siRNAs (Hs_UBE2Z_2, Hs_UBE2Z_3, Hs_UBE2Z_5, Hs_FLJ13855_4), a pool of four different human FAT10 specific siRNAs (Hs_UBD_1, Hs_UBD_2, Hs_UBD3, Hs_UBD_5), or with a control siRNA (AllStars Negative Control siRNA) by using X-tremeGENE siRNA Transfection Reagent (Roche) (all siRNAs were purchased from QIAGEN, Venlo, Netherlands). On day 1 and 2, cells were transfected with a total amount of 240 nM siRNA, respectively. On the third day, endogenous FAT10 expression was induced (see above). Cells were collected and lysed on the fourth day and used for an anti-FAT10 (4F1) immunoprecipitation, as described above. Western blot analysis was performed with a rabbit pAb against UBA6, a rabbit pAb against UBE1 (both from Enzo Lifesciences, Lausen, Switzerland), a rabbit pAb against USE1 [18], a rabbit pAb against FAT10 [4], or as a loading control with a mouse mAb against β-actin (Abcam, Cambridge, UK). In case of the specific knockdown of FAT10, cells were treated with siRNA at the same time as endogenous FAT10 expression was induced with proinflammatory cytokines as previously described [18].

### *In vitro* conjugation experiments

The *in vitro* reaction buffer contained 20 mM Tris/HCL (pH 7.6), 50 mM NaCl, 10 mM MgCl_2_, 4 mM ATP, 0.1 mM dithiothreitol, 5 U·mL^-1^ inorganic pyrophosphatase, 20 mM creatine phosphate, 4 μg·mL^-1^ creatine phosphokinase (all from Sigma, Buchs, Switzerland), supplemented with 1x protease inhibitor mix (Roche, Rotkreuz, Switzerland). Recombinant proteins (S1 Methods) were added to a final volume of 40 μl of 1x reaction buffer in the following amounts: FLAG-UBA6, 1 μg; 6His-USE1, 6 μg; FAT10, 4 μg; ubiquitin, 25 μg; UBE1, 10 μg. The reaction mixture was incubated at 30°C for 60 min with shaking and 5x gel sample buffer with or without 4% 2-mercaptoethanol (2-ME) was added, where indicated. The proteins were separated on 3–8% gradient Tris/Acetate NuPAGE SDS gels (Invitrogen, Lucerne, Switzerland), and subjected to western blot analysis with a rabbit polyclonal antibody against huFAT10 [4], a rabbit pAb against UBE1 (Enzo Lifesciences, Lausen, Switzerland), a directly peroxidase-conjugated mAb against 6-His, a HRP-conjugated mAb against FLAG M2 (both from Sigma, Buchs, Switzerland), or a pAb against ubiquitin (DakoCytomation, Hamburg, Germany).

### Detection of bulk ubiquitin conjugates

HEK293 cells were transfected with either pcDNA3.1-His-3xFLAG-FAT10 [19], or with pcDNA3.1-His-3xFLAG-FAT10 ΔGG [23], by using TransIT-LTI Transfection Reagent (Mirus), or endogenous FAT10 expression was induced as recently described [23,47]. To maintain constantly high levels of FAT10 until harvest after 72 h, cells were transfected a second time or retreated with proinflammatory cytokines after 48 h. After 24, 48, or 72 h of ectopic expression, cells were harvested, lysed, and cell lysates were subjected to separation on 4–12% gradient Bis/Tris NuPAGE SDS gels (Invitrogen). Western blot analysis was performed using a directly labeled peroxidase-conjugated mAb against HA-7 or a HRP-conjugated mAb against FLAG M2 (both Sigma), or a rabbit pAb against ubiquitin (DakoCytomation, Glostrup, Denmark). Antibody against β-actin (Abcam) was used as a loading control.

## Supporting Information

S1 Fig The UBE1-FAT10 conjugate resists denaturing conditions. (TIF)

S2 Fig *In vitro* loading of recombinant FAT10 or 6His-ubiquitin onto recombinant 6His-USE1. (TIF)

S3 Fig Rescue of UBE1-FAT10 conjugate degradation by different proteasome inhibitors. (TIF)

S1 Methods Materials and Methods for experiments shown in S1 Fig. (S1_A Methods) and Fig. 3 (S1_B Methods). (DOCX)

S1 Table Mass spectrometry analysis of the endogenous UBE1-FAT10 conjugate. (DOCX)
