# Supplementary figures and images for: Conjugation of the Ubiquitin Activating Enzyme UBE1 with the Ubiquitin-Like Modifier FAT10 Targets It for Proteasomal Degradation
Source: PLoS One. 2015 Mar 13;10(3):e0120329. doi: 10.1371/journal.pone.0120329 (PMC4359146; doi:10.1371/journal.pone.0120329)

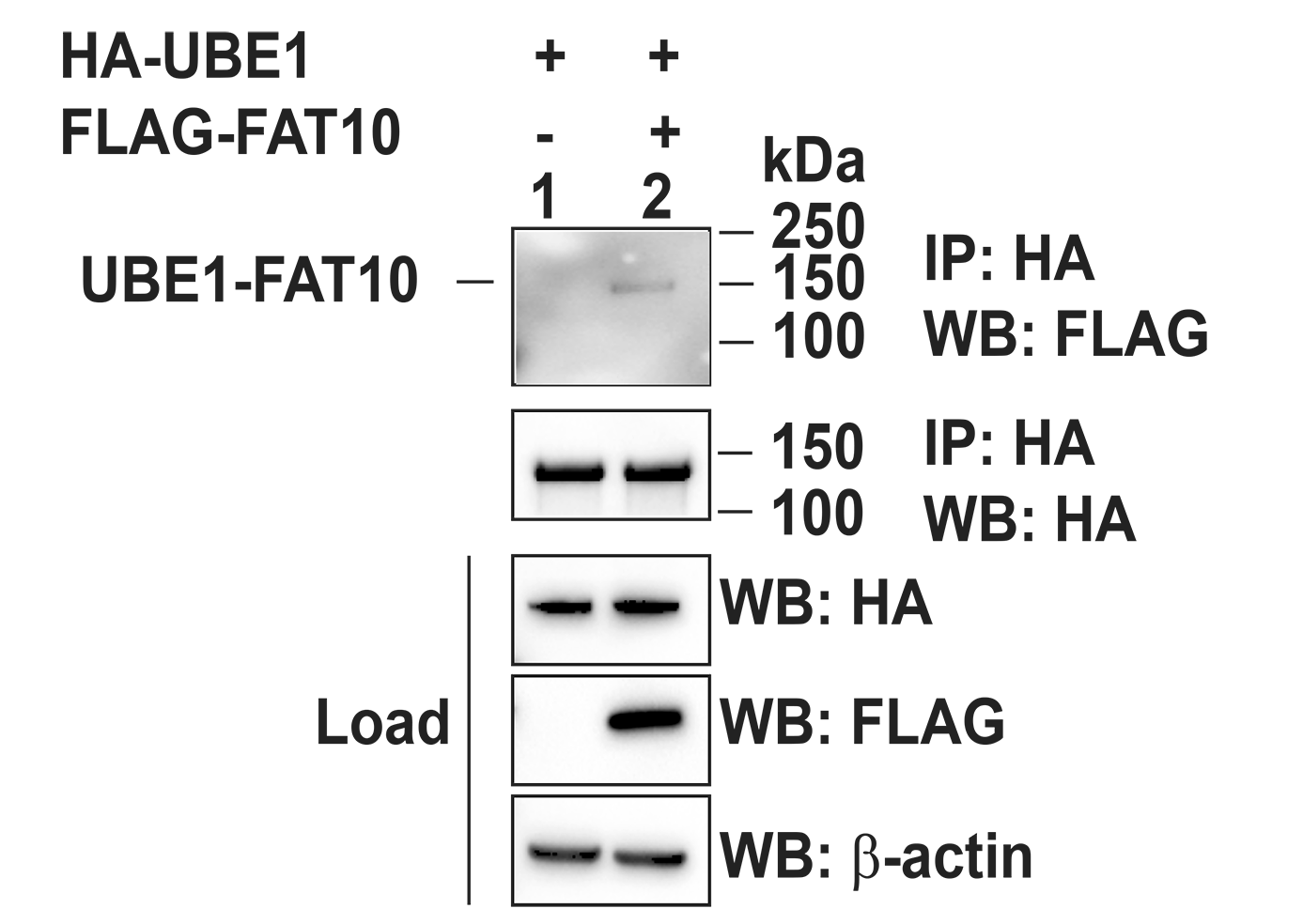

Supplement: S1 Fig — (TIF) [file pone.0120329.s001.tif]

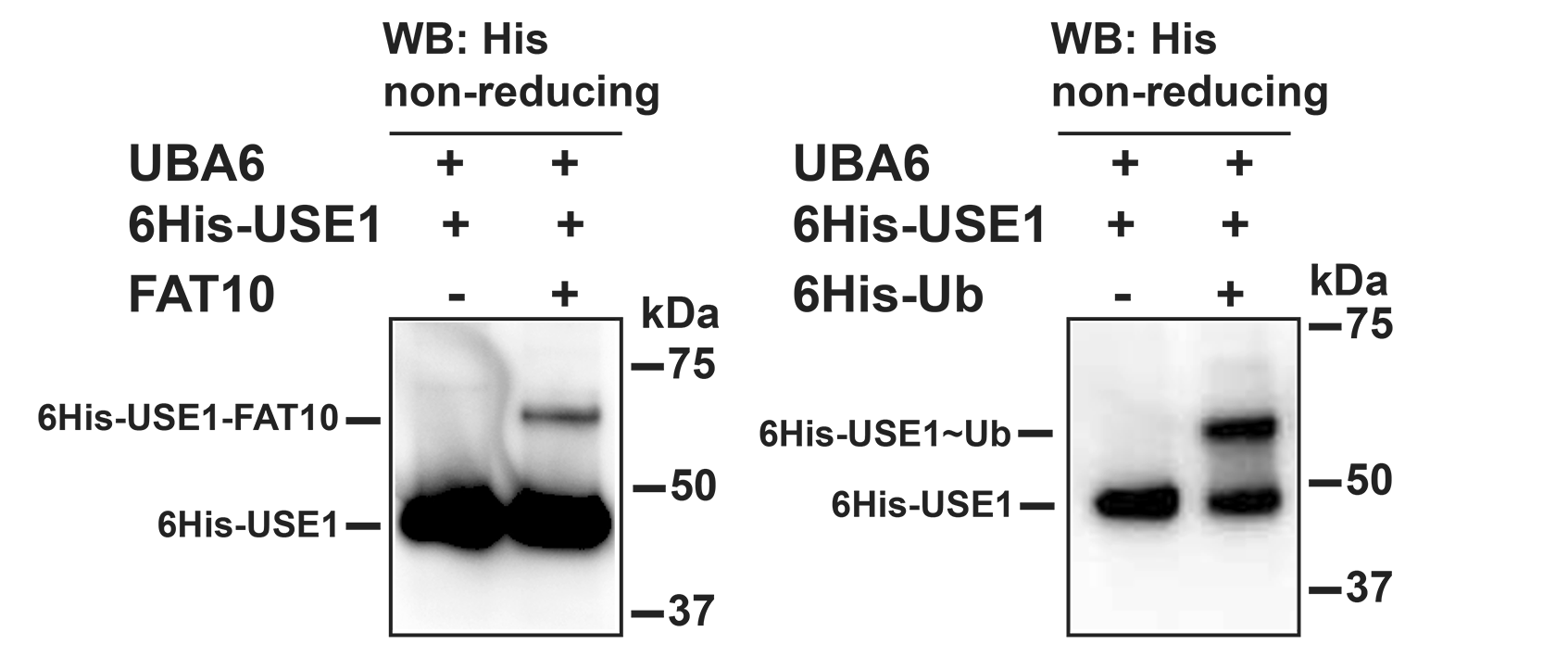

Supplement: S2 Fig — (TIF) [file pone.0120329.s002.tif]

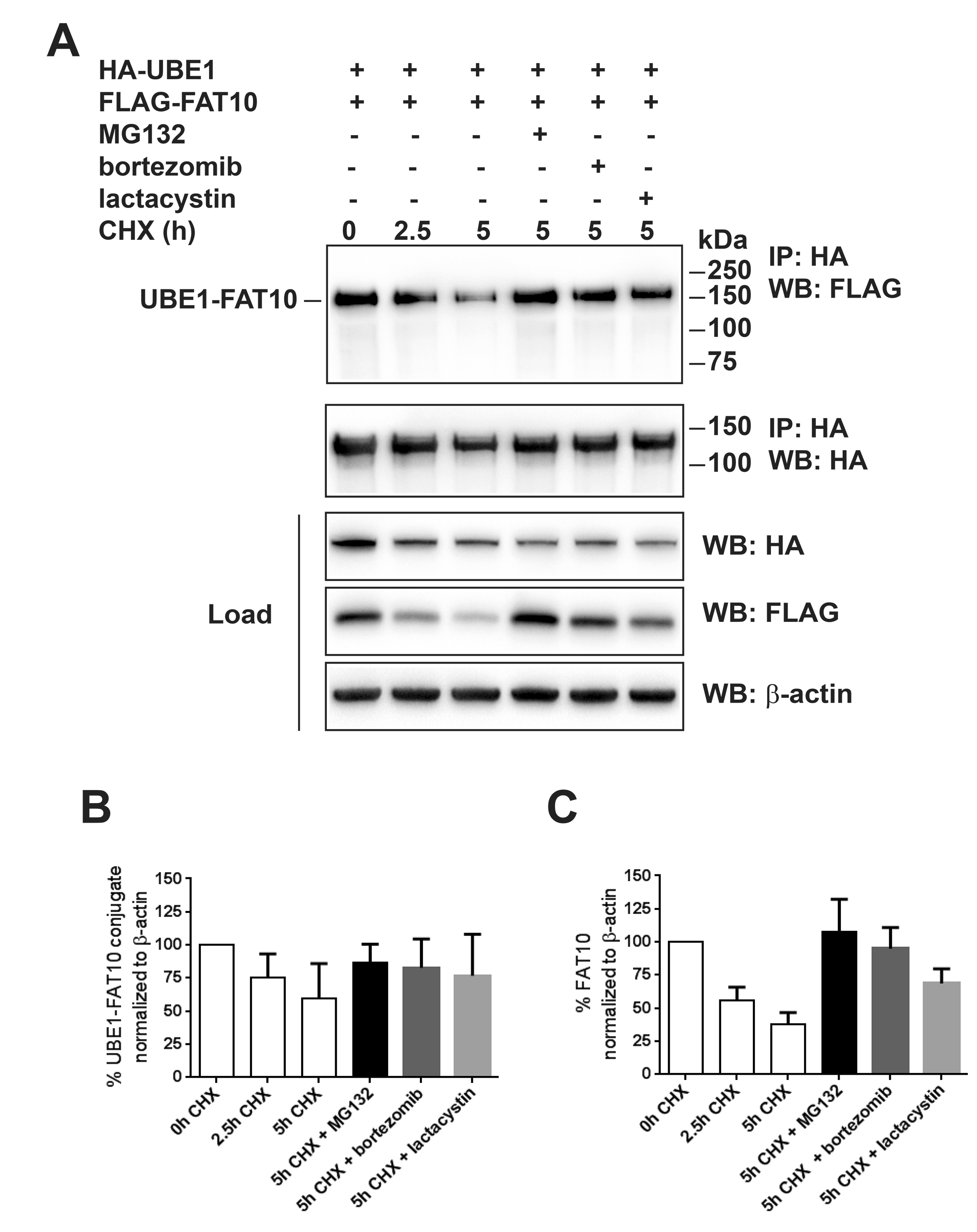

Supplement: S3 Fig — (TIF) [file pone.0120329.s003.tif]
