# Supplementary material for: Conjugation of the Ubiquitin Activating Enzyme UBE1 with the Ubiquitin-Like Modifier FAT10 Targets It for Proteasomal Degradation
Source: PLoS One. 2015 Mar 13;10(3):e0120329. doi: 10.1371/journal.pone.0120329 (PMC4359146; doi:10.1371/journal.pone.0120329)
Supplement: S1 Methods — (DOCX) [file pone.0120329.s004.docx]

**S1 Methods**

**S1_A: Denaturing immunoprecipitation of FAT10ylated UBE1**

HEK293 cells were transiently transfected with pcDNA3.1-HA-UBE1 (kindly provided by Dr. M. Scheffner, University of Konstanz, Germany), and pcDNA3.1-His-3xFLAG-FAT10 [[1](#_ENREF_1)]. After 24 hours of ectopic expression, cells were directly lysed in denaturing buffer (2% SDS, 150 mM NaCl, 10 mM Tris/HCL, 10 mM N-ethylmaleimide (NEM), 0.1 mM phenylmethylsulfonyl fluoride (PMSF), pH 7.5) and lysates were diluted by adding Nonidet P-40 buffer (50 mM Tris/HCL, 0.5% Nonidet P-40, 150 mM NaCl, pH 7.5). Samples were sonicated and clarified by centrifugation. Immunoprecipitation was performed by using anti-HA-agarose conjugate HA‑7 (Sigma), followed by 5 times wash with Nonidet P-40 buffer. Proteins were separated on 4-12% gradient Bis/Tris NuPAGE SDS gels (Invitrogen), and subjected to western blot analysis, using a directly labeled peroxidase-conjugated mAb against HA-7 or a directly HRP-conjugated mAb against FLAG M2 (both Sigma). β-actin (Abcam) was used as a loading control.

**S1_B: Recombinant proteins and purification of recombinant UBE1**

Recombinant human FLAG-UBA6 and ubiquitin were purchased from Enzo Lifesciences. 6His-USE1 was purified as described recently from *E. coli* B834(DE3)pLysS (Novagen) [[2](#_ENREF_2)] transformed with pDEST17-USE1 [[3](#_ENREF_3)] by a standard Ni^2+^-NTA purification protocol. Recombinant tagless human FAT10 was purified as described recently from *E. coli* BL21(DE3) transformed with pSUMO-FAT10 [[4](#_ENREF_4)]. For the expression and purification of recombinant human UBE1 the pcDNA3.1-HA-UBE1 plasmid, kindly provided by Dr. Martin Scheffner (University of Konstanz, Germany), was used as template for polymerase chain reaction with primers 5’-CCAGTGGGTCTCAGGTGGTTCCAGCTCGCCGCTGTCCAAGAAACGTCG C-3’ and 5’-CCCAAGCTTTCAGCGGATGGTGTATCGGACATAGGGAACC-3’, generating a UBE1 amplicon with BsaI and HindIII recognition sites at the 5’ and 3’ end, respectively. The amplicon was inserted 3’ of 6xHis-SUMO [[4](#_ENREF_4)] into the BsaI/HindIII digested pSUMO plasmid, propagated in dam^-^/dcm^-^ *E. coli* strain GM2163. The plasmid was verified by sequencing (Microsynth AG, Balgach, Switzerland). UBE1 expression was performed in *E. coli* BL21(DE3) for 5 hours at room temperature and expression was induced with 0.4 mM Isopropyl-β-D-thiogalactopyranoside (IPTG). Cell pellets were resuspended in 5 ml/g pellet of binding buffer (20 mM Na_3_HPO_4_, 150 mM NaCl, 40 mM imidazole, 5 mM 2-mercaptoethanol, pH 7.5) supplemented with 1 μM protease inhibitor PMSF (Sigma) and 1x protease inhibitor mix (Roche). Cell lysis was performed with three cycles at 2.5 kbar in a cell disrupter (Constant Cell Disrupter TS, Constant Systems Ltd.) and two subsequent filtration steps through filters with 2 μm and 0.2 μm particle pores, respectively. In a first purification step, the filtered lysate was loaded onto a pre-equilibrated 5 ml HisTrap FF column (GE Healthcare) using AektaExplorer (GE Healthcare) driven by the UNICORN software (GE Healthcare). Unspecifically bound proteins were removed by washing the column with ten column volumes of binding buffer and 5 column volumes of binding buffer containing 5% of elution buffer (20 mM Na_3_HPO_4_, 150 mM NaCl, 0.5 M imidazole, 5 mM 2-mercaptoethanol, pH 7.5). Elution was performed with five column volumes of elution buffer. To remove imidazole by size exclusion chromatography from the eluate containing 6xHis-SUMO-UBE1, the eluate was loaded on a HiPrep 26/10 Desalting column (GE Healthcare) equilibrated in binding buffer and eluted with binding buffer until baseline. For removal of the 6xHis-SUMO-tag, the total protein concentration measured by 280 nm absorbance using Spectrophotometer ND-100 (NanoDrop) and 4 μg ULP-1-6xHis/mg protein was added and incubated overnight at 4°C on a roller. Samples were filtered through a filter with 0.45 μm pore size to remove precipitated proteins before loading onto a 5 ml HisTrap FF column equilibrated in binding buffer for tag removal. Elution of untagged recombinant UBE1 was achieved by collecting the flow through while loading the sample on the column. The 6xHis-SUMO-tag and the ULP-1-6xHis were eluted with 100% elution buffer. To keep the stability of recombinant UBE1, a final buffer exchange with a HiPrep 26/10 column was performed and recombinant UBE1 was eluted in 2 column volumes of storage buffer (20 mM Na_3_HPO_4_, 150 mM NaCl, 5 mM 2-mercaptoethanol, pH 7.5). For long time storage at -80°C, glycerol was added to a final concentration of 5% and the protein was concentrated to 0.9 mg/ml using Amicon Ultra Centrifugal Filters (Millipore) with a molecular cut off of 50 kDa. Aliquots were shock frozen in liquid nitrogen and stored at - 80°C.

**References**

1. Chiu YH, Sun Q, Chen ZJ. E1-L2 activates both ubiquitin and FAT10. Mol Cell. 2007; 27: 1014-1023.

2. Aichem A, Pelzer C, Lukasiak S, Kalveram B, Sheppard PW, Rani N, et al. USE1 is a bispecific conjugating enzyme for ubiquitin and FAT10, which FAT10ylates itself *in cis*. Nat Commun. 2010; 1:13: DOI:10.1038/ncomms1012.

3. Jin J, Li X, Gygi SP, Harper JW. Dual E1 activation systems for ubiquitin differentially regulate E2 enzyme charging. Nature. 2007; 447: 1135-1138.

4. Aichem A, Catone N, Groettrup M. Investigations into the auto-FAT10ylation of the bispecific E2 conjugating enzyme UBA6-specific E2 enzyme 1. FEBS J. 2014; 281: 1848-1859.
