# Supplementary material for: Conjugation of the Ubiquitin Activating Enzyme UBE1 with the Ubiquitin-Like Modifier FAT10 Targets It for Proteasomal Degradation
Source: PLoS One. 2015 Mar 13;10(3):e0120329. doi: 10.1371/journal.pone.0120329 (PMC4359146; doi:10.1371/journal.pone.0120329)
Supplement: S1 Table — (DOCX) [file pone.0120329.s005.docx]

**S1 Table**. Mass spectrometry analysis of the endogenous UBE1-FAT10 conjugate.


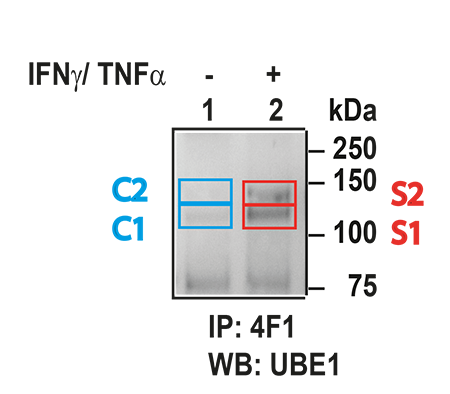


**Sample S1:**

| \| Accession \| \| --- \| \| O43795 \| \| P53396 \| \| P42285 \| \| O95071 \| \| Q9P0K7 \| \| O00159 \| \| P09874 \| \| Q9BPX3 \| \| Q15029 \| \| Q6P1N0 \| \| P78347 \| \| P58107 \| \| Q15149 \| \| Q9Y2A7 \| \| Q15393 \| \| O95373 \| \| O00203 \| \| Q8TF46 \| \| Q16531 \| \| Q14152 \| \| Q99613 \| \| Q9H9Y6 \| \| Q86XI2 \| \| P33176 \| \| Q14527 \| \| O00410 \| \| P51530 \| \| O15205 \| \| Q12768 \| \| Q92974 \| \| Q99575 \| \| P15924 \| \| P22314 \| \| P0CG48 \| \| Q9BTW9 \| \| Q9BZH6 \| \| P98175 \| \| Q96DT7 \| \| O60231 \| \| Q9BSJ8 \| \| Q16513 \| \| P52701 \| \| Q8IWX8 \| \| Q8WUM0 \| \| O75400 \| \| Q05397 \| \| Q9BXP5 \| \| Q00839 \| \| Q02241 \| \| Q9HCE1 \| \| Q86XP3 \| \| Q5H9R7 \| \| P13639 \| \| P51610 \| \| P53992 \| \| Q9C0B7 \| \| Q86YS7 \| \| Q3KQU3 \| \| Q8IUD2 \| \| Q9UPP1 \| \| Q96P70 \| \| O75122 \| \| Q86W56 \| \| Q13813 \| \| Q9Y2D5 \| \| Q86VP6 \| \| Q08211 \| \| O15042 \| \| Q8N163 \| \| Q6VY07 \| \| O60518 \| \| Q8IWC1 \| \| O94832 \| \| Q9H0A0 \| \| P08670 \| \| Q8IVF7 \| \| P28340 \| \| O94972 \| \| Q7KZF4 \| \| Q69YQ0 \| \| Q9Y5B6 \| \| Q92900 \| \| Q8IXT5 \| \| O75694 \| \| Q86W92 \| \| Q9NZC9 \| \| Q9Y6K5 \| \| Q8WVB6 \| \| Q9NQW6 \| \| P43243 \| \| Q8TEX9 \| \| Q9NX05 \| \| P49756 \| \| Q9Y2L1 \| \| Q9Y4E8 \| \| P55884 \| \| Q96QU8 \| \| Q86UV5 \| \| P57740 \| \| Q8N960 \| \| Q6PKG0 \| \| Q9BQE3 \| \| P49792 \| \| P04350 \| \| P35749 \| \| Q14562 \| \| Q9H2U1 \| \| P38935 \| \| Q01082 \| \| Q93100 \| \| P12814 \| \| Q96T76 \| \| O00267 \| \| P05023 \| \| Q8TEW0 \| \| Q14679 \| \| A0AVT1 \| \| Q15386 \| \| P05141 \| \| O94906 \| \| Q5M775 \| \| P69849 \| \| P68104 \| \| O94762 \| \| Q12955 \| \| P30876 \| \|  \| | \| Coverage \| \| --- \| \| 23.77 \| \| 21.98 \| \| 18.04 \| \| 12.43 \| \| 28.47 \| \| 24.65 \| \| 12.43 \| \| 13.60 \| \| 21.71 \| \| 16.09 \| \| 17.43 \| \| 14.46 \| \| 4.29 \| \| 15.51 \| \| 9.61 \| \| 7.32 \| \| 17.18 \| \| 11.29 \| \| 16.67 \| \| 6.87 \| \| 9.97 \| \| 11.63 \| \| 9.36 \| \| 10.90 \| \| 6.44 \| \| 7.66 \| \| 7.36 \| \| 41.82 \| \| 6.64 \| \| 7.40 \| \| 7.23 \| \| 5.33 \| \| 11.44 \| \| 52.55 \| \| 5.29 \| \| 4.74 \| \| 6.77 \| \| 9.87 \| \| 7.88 \| \| 7.34 \| \| 8.54 \| \| 4.41 \| \| 8.52 \| \| 4.93 \| \| 4.60 \| \| 3.90 \| \| 4.11 \| \| 3.27 \| \| 5.94 \| \| 1.99 \| \| 6.29 \| \| 7.10 \| \| 7.46 \| \| 5.16 \| \| 4.66 \| \| 6.58 \| \| 5.50 \| \| 2.97 \| \| 2.87 \| \| 3.21 \| \| 4.51 \| \| 2.16 \| \| 6.25 \| \| 2.02 \| \| 6.05 \| \| 3.01 \| \| 2.60 \| \| 2.53 \| \| 6.72 \| \| 3.74 \| \| 3.53 \| \| 4.91 \| \| 5.57 \| \| 3.02 \| \| 10.09 \| \| 4.38 \| \| 3.97 \| \| 2.49 \| \| 2.86 \| \| 2.86 \| \| 3.82 \| \| 4.52 \| \| 3.50 \| \| 2.80 \| \| 3.86 \| \| 2.20 \| \| 2.02 \| \| 2.67 \| \| 3.47 \| \| 4.37 \| \| 4.44 \| \| 3.56 \| \| 3.68 \| \| 3.55 \| \| 2.96 \| \| 2.70 \| \| 1.96 \| \| 3.09 \| \| 2.16 \| \| 3.04 \| \| 3.56 \| \| 4.23 \| \| 0.78 \| \| 4.50 \| \| 0.86 \| \| 1.72 \| \| 3.57 \| \| 4.73 \| \| 0.68 \| \| 3.29 \| \| 2.91 \| \| 2.43 \| \| 2.12 \| \| 2.05 \| \| 1.47 \| \| 2.17 \| \| 2.28 \| \| 1.85 \| \| 7.05 \| \| 2.02 \| \| 1.97 \| \| 2.37 \| \| 4.11 \| \| 2.32 \| \| 0.46 \| \| 1.79 \| \|  \| | \| # PSMs \| \| --- \| \| 64 \| \| 45 \| \| 42 \| \| 57 \| \| 44 \| \| 41 \| \| 28 \| \| 21 \| \| 34 \| \| 23 \| \| 25 \| \| 21 \| \| 29 \| \| 22 \| \| 18 \| \| 12 \| \| 24 \| \| 22 \| \| 26 \| \| 17 \| \| 13 \| \| 16 \| \| 15 \| \| 19 \| \| 9 \| \| 12 \| \| 12 \| \| 13 \| \| 10 \| \| 13 \| \| 12 \| \| 19 \| \| 15 \| \| 21 \| \| 9 \| \| 11 \| \| 10 \| \| 9 \| \| 14 \| \| 10 \| \| 11 \| \| 8 \| \| 9 \| \| 8 \| \| 8 \| \| 9 \| \| 5 \| \| 5 \| \| 7 \| \| 6 \| \| 8 \| \| 10 \| \| 8 \| \| 13 \| \| 5 \| \| 7 \| \| 7 \| \| 4 \| \| 4 \| \| 6 \| \| 5 \| \| 4 \| \| 10 \| \| 8 \| \| 6 \| \| 5 \| \| 5 \| \| 4 \| \| 8 \| \| 5 \| \| 10 \| \| 7 \| \| 10 \| \| 5 \| \| 7 \| \| 8 \| \| 5 \| \| 4 \| \| 4 \| \| 5 \| \| 5 \| \| 8 \| \| 5 \| \| 5 \| \| 6 \| \| 3 \| \| 4 \| \| 4 \| \| 5 \| \| 5 \| \| 5 \| \| 4 \| \| 5 \| \| 6 \| \| 6 \| \| 4 \| \| 4 \| \| 4 \| \| 4 \| \| 3 \| \| 5 \| \| 4 \| \| 2 \| \| 2 \| \| 4 \| \| 3 \| \| 5 \| \| 5 \| \| 3 \| \| 5 \| \| 2 \| \| 3 \| \| 3 \| \| 3 \| \| 3 \| \| 2 \| \| 3 \| \| 3 \| \| 2 \| \| 2 \| \| 3 \| \| 2 \| \| 2 \| \| 2 \| \| 2 \| \| 2 \| \|  \| | \| # Peptides \| \| --- \| \| 23 \| \| 18 \| \| 15 \| \| 25 \| \| 21 \| \| 20 \| \| 10 \| \| 10 \| \| 16 \| \| 11 \| \| 13 \| \| 8 \| \| 16 \| \| 13 \| \| 9 \| \| 6 \| \| 15 \| \| 10 \| \| 14 \| \| 8 \| \| 7 \| \| 10 \| \| 9 \| \| 9 \| \| 5 \| \| 6 \| \| 7 \| \| 5 \| \| 6 \| \| 6 \| \| 6 \| \| 13 \| \| 8 \| \| 4 \| \| 5 \| \| 5 \| \| 5 \| \| 6 \| \| 7 \| \| 6 \| \| 6 \| \| 5 \| \| 6 \| \| 4 \| \| 3 \| \| 4 \| \| 3 \| \| 2 \| \| 4 \| \| 2 \| \| 5 \| \| 5 \| \| 5 \| \| 7 \| \| 3 \| \| 5 \| \| 4 \| \| 2 \| \| 3 \| \| 3 \| \| 4 \| \| 2 \| \| 5 \| \| 4 \| \| 3 \| \| 3 \| \| 3 \| \| 2 \| \| 5 \| \| 3 \| \| 3 \| \| 4 \| \| 5 \| \| 3 \| \| 4 \| \| 4 \| \| 4 \| \| 2 \| \| 2 \| \| 3 \| \| 3 \| \| 5 \| \| 3 \| \| 3 \| \| 3 \| \| 2 \| \| 2 \| \| 2 \| \| 3 \| \| 3 \| \| 4 \| \| 3 \| \| 3 \| \| 3 \| \| 3 \| \| 2 \| \| 2 \| \| 3 \| \| 2 \| \| 3 \| \| 3 \| \| 2 \| \| 2 \| \| 2 \| \| 2 \| \| 2 \| \| 3 \| \| 4 \| \| 2 \| \| 3 \| \| 2 \| \| 2 \| \| 2 \| \| 2 \| \| 2 \| \| 2 \| \| 2 \| \| 2 \| \| 2 \| \| 2 \| \| 2 \| \| 2 \| \| 2 \| \| 2 \| \| 2 \| \| 2 \| \|  \| | \| # AAs \| \| --- \| \| 1136 \| \| 1101 \| \| 1042 \| \| 2799 \| \| 980 \| \| 1063 \| \| 1014 \| \| 1015 \| \| 972 \| \| 951 \| \| 998 \| \| 5090 \| \| 4684 \| \| 1128 \| \| 1217 \| \| 1038 \| \| 1094 \| \| 1054 \| \| 1140 \| \| 1382 \| \| 913 \| \| 1135 \| \| 1143 \| \| 963 \| \| 1009 \| \| 1097 \| \| 1060 \| \| 165 \| \| 1159 \| \| 986 \| \| 1024 \| \| 2871 \| \| 1058 \| \| 685 \| \| 1192 \| \| 1224 \| \| 930 \| \| 871 \| \| 1041 \| \| 1104 \| \| 984 \| \| 1360 \| \| 916 \| \| 1156 \| \| 957 \| \| 1052 \| \| 876 \| \| 825 \| \| 960 \| \| 1003 \| \| 938 \| \| 873 \| \| 858 \| \| 2035 \| \| 1094 \| \| 1094 \| \| 1000 \| \| 841 \| \| 1116 \| \| 1060 \| \| 1041 \| \| 1294 \| \| 976 \| \| 2472 \| \| 859 \| \| 1230 \| \| 1270 \| \| 1029 \| \| 923 \| \| 963 \| \| 1105 \| \| 876 \| \| 1006 \| \| 1025 \| \| 466 \| \| 1028 \| \| 1107 \| \| 964 \| \| 910 \| \| 1117 \| \| 917 \| \| 1129 \| \| 1001 \| \| 1391 \| \| 1011 \| \| 954 \| \| 1087 \| \| 975 \| \| 1124 \| \| 847 \| \| 1081 \| \| 1096 \| \| 843 \| \| 958 \| \| 981 \| \| 814 \| \| 1125 \| \| 1035 \| \| 925 \| \| 986 \| \| 1096 \| \| 449 \| \| 3224 \| \| 444 \| \| 1972 \| \| 1220 \| \| 1008 \| \| 993 \| \| 2364 \| \| 1093 \| \| 892 \| \| 1030 \| \| 1087 \| \| 1023 \| \| 1356 \| \| 1199 \| \| 1052 \| \| 1083 \| \| 298 \| \| 941 \| \| 1068 \| \| 1222 \| \| 462 \| \| 991 \| \| 4377 \| \| 1174 \| \|  \| | \| MW [kDa] \| \| --- \| \| 131.9 \| \| 120.8 \| \| 117.7 \| \| 309.2 \| \| 110.0 \| \| 121.6 \| \| 113.0 \| \| 114.3 \| \| 109.4 \| \| 104.0 \| \| 112.3 \| \| 555.3 \| \| 531.5 \| \| 128.7 \| \| 135.5 \| \| 119.4 \| \| 121.2 \| \| 120.7 \| \| 126.9 \| \| 166.5 \| \| 105.3 \| \| 128.1 \| \| 130.9 \| \| 109.6 \| \| 113.9 \| \| 123.5 \| \| 120.3 \| \| 18.5 \| \| 134.2 \| \| 111.5 \| \| 114.6 \| \| 331.6 \| \| 117.8 \| \| 77.0 \| \| 132.5 \| \| 136.6 \| \| 103.5 \| \| 94.8 \| \| 119.2 \| \| 122.8 \| \| 112.0 \| \| 152.7 \| \| 103.6 \| \| 128.9 \| \| 108.7 \| \| 119.2 \| \| 100.6 \| \| 90.5 \| \| 110.0 \| \| 113.6 \| \| 102.9 \| \| 97.6 \| \| 95.3 \| \| 208.6 \| \| 118.2 \| \| 120.7 \| \| 110.4 \| \| 92.8 \| \| 128.0 \| \| 117.8 \| \| 115.9 \| \| 141.0 \| \| 111.0 \| \| 284.4 \| \| 94.6 \| \| 136.3 \| \| 140.9 \| \| 118.2 \| \| 102.8 \| \| 104.8 \| \| 124.6 \| \| 98.4 \| \| 116.1 \| \| 115.7 \| \| 53.6 \| \| 117.1 \| \| 123.6 \| \| 107.8 \| \| 101.9 \| \| 124.5 \| \| 104.7 \| \| 124.3 \| \| 118.0 \| \| 155.1 \| \| 114.0 \| \| 105.9 \| \| 121.1 \| \| 107.3 \| \| 124.1 \| \| 94.6 \| \| 118.6 \| \| 120.5 \| \| 100.1 \| \| 108.9 \| \| 112.3 \| \| 92.4 \| \| 128.8 \| \| 119.0 \| \| 106.3 \| \| 112.6 \| \| 123.4 \| \| 49.9 \| \| 358.0 \| \| 49.6 \| \| 227.2 \| \| 139.2 \| \| 114.7 \| \| 109.1 \| \| 274.4 \| \| 124.8 \| \| 103.0 \| \| 113.2 \| \| 120.9 \| \| 112.8 \| \| 151.3 \| \| 133.3 \| \| 117.9 \| \| 123.8 \| \| 32.8 \| \| 106.9 \| \| 118.5 \| \| 134.0 \| \| 50.1 \| \| 108.8 \| \| 480.1 \| \| 133.8 \| \|  \| | \| calc. pI \| \| --- \| \| 9.38 \| \| 7.33 \| \| 6.52 \| \| 5.85 \| \| 6.21 \| \| 9.41 \| \| 8.88 \| \| 5.59 \| \| 5.00 \| \| 8.09 \| \| 6.39 \| \| 5.60 \| \| 5.96 \| \| 6.62 \| \| 5.26 \| \| 4.82 \| \| 6.04 \| \| 6.54 \| \| 5.26 \| \| 6.79 \| \| 5.68 \| \| 7.83 \| \| 6.87 \| \| 6.51 \| \| 8.60 \| \| 4.94 \| \| 7.74 \| \| 8.90 \| \| 6.98 \| \| 7.27 \| \| 9.22 \| \| 6.81 \| \| 5.76 \| \| 7.66 \| \| 6.19 \| \| 6.92 \| \| 5.97 \| \| 5.16 \| \| 6.80 \| \| 5.83 \| \| 6.30 \| \| 6.90 \| \| 9.04 \| \| 5.10 \| \| 7.56 \| \| 6.62 \| \| 5.96 \| \| 6.00 \| \| 8.51 \| \| 8.82 \| \| 7.02 \| \| 4.60 \| \| 6.83 \| \| 7.46 \| \| 7.06 \| \| 6.11 \| \| 5.69 \| \| 10.11 \| \| 5.97 \| \| 8.72 \| \| 4.81 \| \| 8.47 \| \| 6.43 \| \| 5.35 \| \| 5.11 \| \| 5.78 \| \| 6.84 \| \| 8.47 \| \| 5.22 \| \| 7.74 \| \| 5.01 \| \| 9.32 \| \| 9.39 \| \| 8.27 \| \| 5.12 \| \| 6.65 \| \| 7.03 \| \| 5.15 \| \| 7.17 \| \| 5.72 \| \| 5.68 \| \| 6.61 \| \| 6.81 \| \| 6.16 \| \| 5.55 \| \| 9.06 \| \| 8.40 \| \| 7.21 \| \| 8.07 \| \| 6.25 \| \| 4.96 \| \| 9.03 \| \| 6.32 \| \| 7.14 \| \| 5.22 \| \| 5.00 \| \| 6.35 \| \| 6.05 \| \| 5.43 \| \| 6.25 \| \| 8.82 \| \| 5.10 \| \| 6.20 \| \| 4.88 \| \| 5.50 \| \| 8.32 \| \| 7.68 \| \| 8.97 \| \| 5.57 \| \| 6.95 \| \| 5.41 \| \| 6.35 \| \| 5.06 \| \| 5.49 \| \| 7.68 \| \| 8.85 \| \| 6.14 \| \| 6.71 \| \| 9.69 \| \| 8.25 \| \| 6.70 \| \| 5.67 \| \| 9.01 \| \| 8.56 \| \| 6.49 \| \| 6.87 \| \|  \| | \| Score \| \| --- \| \| 1286.82 \| \| 1138.11 \| \| 1026.28 \| \| 981.85 \| \| 851.59 \| \| 811.84 \| \| 770.05 \| \| 729.45 \| \| 673.96 \| \| 557.29 \| \| 501.96 \| \| 477.45 \| \| 456.70 \| \| 451.38 \| \| 434.35 \| \| 413.31 \| \| 402.54 \| \| 358.89 \| \| 352.58 \| \| 342.85 \| \| 338.75 \| \| 338.44 \| \| 314.83 \| \| 309.35 \| \| 277.86 \| \| 273.31 \| \| 271.22 \| \| 268.86 \| \| 256.31 \| \| 242.54 \| \| 239.03 \| \| 224.13 \| \| 223.40 \| \| 214.14 \| \| 206.69 \| \| 192.42 \| \| 185.98 \| \| 182.57 \| \| 179.36 \| \| 178.81 \| \| 176.58 \| \| 175.35 \| \| 175.18 \| \| 171.68 \| \| 166.69 \| \| 154.86 \| \| 153.80 \| \| 151.30 \| \| 151.26 \| \| 138.74 \| \| 138.67 \| \| 136.02 \| \| 135.62 \| \| 134.97 \| \| 132.44 \| \| 131.03 \| \| 128.77 \| \| 127.92 \| \| 127.11 \| \| 126.26 \| \| 121.58 \| \| 121.00 \| \| 120.18 \| \| 117.81 \| \| 115.64 \| \| 115.57 \| \| 113.61 \| \| 112.79 \| \| 111.00 \| \| 106.84 \| \| 105.82 \| \| 105.17 \| \| 104.74 \| \| 103.08 \| \| 101.49 \| \| 100.96 \| \| 100.73 \| \| 100.21 \| \| 98.85 \| \| 96.07 \| \| 95.81 \| \| 92.31 \| \| 92.01 \| \| 91.55 \| \| 89.01 \| \| 82.81 \| \| 80.58 \| \| 77.71 \| \| 76.64 \| \| 75.53 \| \| 69.74 \| \| 68.32 \| \| 66.97 \| \| 66.94 \| \| 66.81 \| \| 66.07 \| \| 63.27 \| \| 60.63 \| \| 60.53 \| \| 57.24 \| \| 54.69 \| \| 54.32 \| \| 53.39 \| \| 51.48 \| \| 50.79 \| \| 49.71 \| \| 49.37 \| \| 46.28 \| \| 44.63 \| \| 43.43 \| \| 42.62 \| \| 39.60 \| \| 38.36 \| \| 37.55 \| \| 37.20 \| \| 36.72 \| \| 36.18 \| \| 35.58 \| \| 34.05 \| \| 32.73 \| \| 28.91 \| \| 28.07 \| \| 26.28 \| \| 25.25 \| \| 21.89 \| \| 19.68 \| \|  \| | \| Description \| \| --- \| \| Unconventional myosin-Ib OS=Homo sapiens GN=MYO1B PE=2 SV=3 - [MYO1B_HUMAN] \| \| ATP-citrate synthase OS=Homo sapiens GN=ACLY PE=1 SV=3 - [ACLY_HUMAN] \| \| Superkiller viralicidic activity 2-like 2 OS=Homo sapiens GN=SKIV2L2 PE=1 SV=3 - [SK2L2_HUMAN] \| \| E3 ubiquitin-protein ligase UBR5 OS=Homo sapiens GN=UBR5 PE=1 SV=2 - [UBR5_HUMAN] \| \| Ankycorbin OS=Homo sapiens GN=RAI14 PE=1 SV=2 - [RAI14_HUMAN] \| \| Unconventional myosin-Ic OS=Homo sapiens GN=MYO1C PE=1 SV=4 - [MYO1C_HUMAN] \| \| Poly [ADP-ribose] polymerase 1 OS=Homo sapiens GN=PARP1 PE=1 SV=4 - [PARP1_HUMAN] \| \| Condensin complex subunit 3 OS=Homo sapiens GN=NCAPG PE=1 SV=1 - [CND3_HUMAN] \| \| 116 kDa U5 small nuclear ribonucleoprotein component OS=Homo sapiens GN=EFTUD2 PE=1 SV=1 - [U5S1_HUMAN] \| \| Coiled-coil and C2 domain-containing protein 1A OS=Homo sapiens GN=CC2D1A PE=1 SV=1 - [C2D1A_HUMAN] \| \| General transcription factor II-I OS=Homo sapiens GN=GTF2I PE=1 SV=2 - [GTF2I_HUMAN] \| \| Epiplakin OS=Homo sapiens GN=EPPK1 PE=1 SV=2 - [EPIPL_HUMAN] \| \| Plectin OS=Homo sapiens GN=PLEC PE=1 SV=3 - [PLEC_HUMAN] \| \| Nck-associated protein 1 OS=Homo sapiens GN=NCKAP1 PE=1 SV=1 - [NCKP1_HUMAN] \| \| Splicing factor 3B subunit 3 OS=Homo sapiens GN=SF3B3 PE=1 SV=4 - [SF3B3_HUMAN] \| \| Importin-7 OS=Homo sapiens GN=IPO7 PE=1 SV=1 - [IPO7_HUMAN] \| \| AP-3 complex subunit beta-1 OS=Homo sapiens GN=AP3B1 PE=1 SV=3 - [AP3B1_HUMAN] \| \| DIS3-like exonuclease 1 OS=Homo sapiens GN=DIS3L PE=1 SV=2 - [DI3L1_HUMAN] \| \| DNA damage-binding protein 1 OS=Homo sapiens GN=DDB1 PE=1 SV=1 - [DDB1_HUMAN] \| \| Eukaryotic translation initiation factor 3 subunit A OS=Homo sapiens GN=EIF3A PE=1 SV=1 - [EIF3A_HUMAN] \| \| Eukaryotic translation initiation factor 3 subunit C OS=Homo sapiens GN=EIF3C PE=1 SV=1 - [EIF3C_HUMAN] \| \| DNA-directed RNA polymerase I subunit RPA2 OS=Homo sapiens GN=POLR1B PE=1 SV=2 - [RPA2_HUMAN] \| \| Condensin-2 complex subunit G2 OS=Homo sapiens GN=NCAPG2 PE=1 SV=1 - [CNDG2_HUMAN] \| \| Kinesin-1 heavy chain OS=Homo sapiens GN=KIF5B PE=1 SV=1 - [KINH_HUMAN] \| \| Helicase-like transcription factor OS=Homo sapiens GN=HLTF PE=1 SV=2 - [HLTF_HUMAN] \| \| Importin-5 OS=Homo sapiens GN=IPO5 PE=1 SV=4 - [IPO5_HUMAN] \| \| DNA2-like helicase OS=Homo sapiens GN=DNA2 PE=1 SV=3 - [DNA2L_HUMAN] \| \| Ubiquitin D OS=Homo sapiens GN=UBD PE=1 SV=2 - [UBD_HUMAN] \| \| WASH complex subunit strumpellin OS=Homo sapiens GN=KIAA0196 PE=1 SV=1 - [STRUM_HUMAN] \| \| Rho guanine nucleotide exchange factor 2 OS=Homo sapiens GN=ARHGEF2 PE=1 SV=4 - [ARHG2_HUMAN] \| \| Ribonucleases P/MRP protein subunit POP1 OS=Homo sapiens GN=POP1 PE=1 SV=2 - [POP1_HUMAN] \| \| Desmoplakin OS=Homo sapiens GN=DSP PE=1 SV=3 - [DESP_HUMAN] \| \| Ubiquitin-like modifier-activating enzyme 1 OS=Homo sapiens GN=UBA1 PE=1 SV=3 - [UBA1_HUMAN] \| \| Polyubiquitin-C OS=Homo sapiens GN=UBC PE=1 SV=3 - [UBC_HUMAN] \| \| Tubulin-specific chaperone D OS=Homo sapiens GN=TBCD PE=1 SV=2 - [TBCD_HUMAN] \| \| WD repeat-containing protein 11 OS=Homo sapiens GN=WDR11 PE=1 SV=1 - [WDR11_HUMAN] \| \| RNA-binding protein 10 OS=Homo sapiens GN=RBM10 PE=1 SV=3 - [RBM10_HUMAN] \| \| Zinc finger and BTB domain-containing protein 10 OS=Homo sapiens GN=ZBTB10 PE=1 SV=2 - [ZBT10_HUMAN] \| \| Putative pre-mRNA-splicing factor ATP-dependent RNA helicase DHX16 OS=Homo sapiens GN=DHX16 PE=1 SV=2 - [DHX16_HUMAN] \| \| Extended synaptotagmin-1 OS=Homo sapiens GN=ESYT1 PE=1 SV=1 - [ESYT1_HUMAN] \| \| Serine/threonine-protein kinase N2 OS=Homo sapiens GN=PKN2 PE=1 SV=1 - [PKN2_HUMAN] \| \| DNA mismatch repair protein Msh6 OS=Homo sapiens GN=MSH6 PE=1 SV=2 - [MSH6_HUMAN] \| \| Calcium homeostasis endoplasmic reticulum protein OS=Homo sapiens GN=CHERP PE=1 SV=3 - [CHERP_HUMAN] \| \| Nuclear pore complex protein Nup133 OS=Homo sapiens GN=NUP133 PE=1 SV=2 - [NU133_HUMAN] \| \| Pre-mRNA-processing factor 40 homolog A OS=Homo sapiens GN=PRPF40A PE=1 SV=2 - [PR40A_HUMAN] \| \| Focal adhesion kinase 1 OS=Homo sapiens GN=PTK2 PE=1 SV=2 - [FAK1_HUMAN] \| \| Serrate RNA effector molecule homolog OS=Homo sapiens GN=SRRT PE=1 SV=1 - [SRRT_HUMAN] \| \| Heterogeneous nuclear ribonucleoprotein U OS=Homo sapiens GN=HNRNPU PE=1 SV=6 - [HNRPU_HUMAN] \| \| Kinesin-like protein KIF23 OS=Homo sapiens GN=KIF23 PE=1 SV=3 - [KIF23_HUMAN] \| \| Putative helicase MOV-10 OS=Homo sapiens GN=MOV10 PE=1 SV=2 - [MOV10_HUMAN] \| \| ATP-dependent RNA helicase DDX42 OS=Homo sapiens GN=DDX42 PE=1 SV=1 - [DDX42_HUMAN] \| \| Serine/threonine-protein phosphatase 6 regulatory subunit 3 OS=Homo sapiens GN=PPP6R3 PE=1 SV=2 - [PP6R3_HUMAN] \| \| Elongation factor 2 OS=Homo sapiens GN=EEF2 PE=1 SV=4 - [EF2_HUMAN] \| \| Host cell factor 1 OS=Homo sapiens GN=HCFC1 PE=1 SV=2 - [HCFC1_HUMAN] \| \| Protein transport protein Sec24C OS=Homo sapiens GN=SEC24C PE=1 SV=3 - [SC24C_HUMAN] \| \| Transmembrane and coiled-coil domain-containing protein 7 OS=Homo sapiens GN=TMCO7 PE=2 SV=2 - [TMCO7_HUMAN] \| \| Uncharacterized protein KIAA0528 OS=Homo sapiens GN=KIAA0528 PE=1 SV=1 - [K0528_HUMAN] \| \| MAP7 domain-containing protein 1 OS=Homo sapiens GN=MAP7D1 PE=1 SV=1 - [MA7D1_HUMAN] \| \| ELKS/Rab6-interacting/CAST family member 1 OS=Homo sapiens GN=ERC1 PE=1 SV=1 - [RB6I2_HUMAN] \| \| Histone lysine demethylase PHF8 OS=Homo sapiens GN=PHF8 PE=1 SV=3 - [PHF8_HUMAN] \| \| Importin-9 OS=Homo sapiens GN=IPO9 PE=1 SV=3 - [IPO9_HUMAN] \| \| CLIP-associating protein 2 OS=Homo sapiens GN=CLASP2 PE=1 SV=2 - [CLAP2_HUMAN] \| \| Poly(ADP-ribose) glycohydrolase OS=Homo sapiens GN=PARG PE=1 SV=1 - [PARG_HUMAN] \| \| Spectrin alpha chain, brain OS=Homo sapiens GN=SPTAN1 PE=1 SV=3 - [SPTA2_HUMAN] \| \| A-kinase anchor protein 2 OS=Homo sapiens GN=AKAP2 PE=1 SV=3 - [AKAP2_HUMAN] \| \| Cullin-associated NEDD8-dissociated protein 1 OS=Homo sapiens GN=CAND1 PE=1 SV=2 - [CAND1_HUMAN] \| \| ATP-dependent RNA helicase A OS=Homo sapiens GN=DHX9 PE=1 SV=4 - [DHX9_HUMAN] \| \| U2 snRNP-associated SURP motif-containing protein OS=Homo sapiens GN=U2SURP PE=1 SV=2 - [SR140_HUMAN] \| \| DBIRD complex subunit KIAA1967 OS=Homo sapiens GN=KIAA1967 PE=1 SV=2 - [K1967_HUMAN] \| \| Phosphofurin acidic cluster sorting protein 1 OS=Homo sapiens GN=PACS1 PE=1 SV=2 - [PACS1_HUMAN] \| \| Ran-binding protein 6 OS=Homo sapiens GN=RANBP6 PE=1 SV=2 - [RNBP6_HUMAN] \| \| MAP7 domain-containing protein 3 OS=Homo sapiens GN=MAP7D3 PE=1 SV=2 - [MA7D3_HUMAN] \| \| Unconventional myosin-Id OS=Homo sapiens GN=MYO1D PE=1 SV=2 - [MYO1D_HUMAN] \| \| N-acetyltransferase 10 OS=Homo sapiens GN=NAT10 PE=1 SV=2 - [NAT10_HUMAN] \| \| Vimentin OS=Homo sapiens GN=VIM PE=1 SV=4 - [VIME_HUMAN] \| \| Formin-like protein 3 OS=Homo sapiens GN=FMNL3 PE=1 SV=3 - [FMNL3_HUMAN] \| \| DNA polymerase delta catalytic subunit OS=Homo sapiens GN=POLD1 PE=1 SV=2 - [DPOD1_HUMAN] \| \| E3 ubiquitin-protein ligase TRIM37 OS=Homo sapiens GN=TRIM37 PE=1 SV=2 - [TRI37_HUMAN] \| \| Staphylococcal nuclease domain-containing protein 1 OS=Homo sapiens GN=SND1 PE=1 SV=1 - [SND1_HUMAN] \| \| Cytospin-A OS=Homo sapiens GN=SPECC1L PE=1 SV=2 - [CYTSA_HUMAN] \| \| GC-rich sequence DNA-binding factor 1 OS=Homo sapiens GN=GCFC1 PE=1 SV=2 - [GCFC1_HUMAN] \| \| Regulator of nonsense transcripts 1 OS=Homo sapiens GN=UPF1 PE=1 SV=2 - [RENT1_HUMAN] \| \| RNA-binding protein 12B OS=Homo sapiens GN=RBM12B PE=1 SV=2 - [RB12B_HUMAN] \| \| Nuclear pore complex protein Nup155 OS=Homo sapiens GN=NUP155 PE=1 SV=1 - [NU155_HUMAN] \| \| Liprin-beta-1 OS=Homo sapiens GN=PPFIBP1 PE=1 SV=2 - [LIPB1_HUMAN] \| \| SWI/SNF-related matrix-associated actin-dependent regulator of chromatin subfamily A-like protein 1 OS=Homo sapiens GN=SMARCAL1 PE=1 SV=1 - [SMAL1_HUMAN] \| \| 2'-5'-oligoadenylate synthase 3 OS=Homo sapiens GN=OAS3 PE=1 SV=3 - [OAS3_HUMAN] \| \| Chromosome transmission fidelity protein 18 homolog OS=Homo sapiens GN=CHTF18 PE=1 SV=1 - [CTF18_HUMAN] \| \| Actin-binding protein anillin OS=Homo sapiens GN=ANLN PE=1 SV=2 - [ANLN_HUMAN] \| \| Matrin-3 OS=Homo sapiens GN=MATR3 PE=1 SV=2 - [MATR3_HUMAN] \| \| Importin-4 OS=Homo sapiens GN=IPO4 PE=1 SV=2 - [IPO4_HUMAN] \| \| Constitutive coactivator of PPAR-gamma-like protein 2 OS=Homo sapiens GN=FAM120C PE=2 SV=3 - [F120C_HUMAN] \| \| RNA-binding protein 25 OS=Homo sapiens GN=RBM25 PE=1 SV=3 - [RBM25_HUMAN] \| \| Exosome complex exonuclease RRP44 OS=Homo sapiens GN=DIS3 PE=1 SV=2 - [RRP44_HUMAN] \| \| Ubiquitin carboxyl-terminal hydrolase 15 OS=Homo sapiens GN=USP15 PE=1 SV=3 - [UBP15_HUMAN] \| \| Eukaryotic translation initiation factor 3 subunit B OS=Homo sapiens GN=EIF3B PE=1 SV=3 - [EIF3B_HUMAN] \| \| Exportin-6 OS=Homo sapiens GN=XPO6 PE=1 SV=1 - [XPO6_HUMAN] \| \| Ubiquitin carboxyl-terminal hydrolase 48 OS=Homo sapiens GN=USP48 PE=1 SV=1 - [UBP48_HUMAN] \| \| Nuclear pore complex protein Nup107 OS=Homo sapiens GN=NUP107 PE=1 SV=1 - [NU107_HUMAN] \| \| Centrosomal protein of 120 kDa OS=Homo sapiens GN=CEP120 PE=2 SV=2 - [CE120_HUMAN] \| \| La-related protein 1 OS=Homo sapiens GN=LARP1 PE=1 SV=2 - [LARP1_HUMAN] \| \| Tubulin alpha-1C chain OS=Homo sapiens GN=TUBA1C PE=1 SV=1 - [TBA1C_HUMAN] \| \| E3 SUMO-protein ligase RanBP2 OS=Homo sapiens GN=RANBP2 PE=1 SV=2 - [RBP2_HUMAN] \| \| Tubulin beta-4A chain OS=Homo sapiens GN=TUBB4A PE=1 SV=2 - [TBB4A_HUMAN] \| \| Myosin-11 OS=Homo sapiens GN=MYH11 PE=1 SV=3 - [MYH11_HUMAN] \| \| ATP-dependent RNA helicase DHX8 OS=Homo sapiens GN=DHX8 PE=1 SV=1 - [DHX8_HUMAN] \| \| Probable ATP-dependent RNA helicase DHX36 OS=Homo sapiens GN=DHX36 PE=1 SV=2 - [DHX36_HUMAN] \| \| DNA-binding protein SMUBP-2 OS=Homo sapiens GN=IGHMBP2 PE=1 SV=3 - [SMBP2_HUMAN] \| \| Spectrin beta chain, brain 1 OS=Homo sapiens GN=SPTBN1 PE=1 SV=2 - [SPTB2_HUMAN] \| \| Phosphorylase b kinase regulatory subunit beta OS=Homo sapiens GN=PHKB PE=1 SV=3 - [KPBB_HUMAN] \| \| Alpha-actinin-1 OS=Homo sapiens GN=ACTN1 PE=1 SV=2 - [ACTN1_HUMAN] \| \| MMS19 nucleotide excision repair protein homolog OS=Homo sapiens GN=MMS19 PE=1 SV=2 - [MMS19_HUMAN] \| \| Transcription elongation factor SPT5 OS=Homo sapiens GN=SUPT5H PE=1 SV=1 - [SPT5H_HUMAN] \| \| Sodium/potassium-transporting ATPase subunit alpha-1 OS=Homo sapiens GN=ATP1A1 PE=1 SV=1 - [AT1A1_HUMAN] \| \| Partitioning defective 3 homolog OS=Homo sapiens GN=PARD3 PE=1 SV=2 - [PARD3_HUMAN] \| \| Tubulin polyglutamylase TTLL4 OS=Homo sapiens GN=TTLL4 PE=1 SV=2 - [TTLL4_HUMAN] \| \| Ubiquitin-like modifier-activating enzyme 6 OS=Homo sapiens GN=UBA6 PE=1 SV=1 - [UBA6_HUMAN] \| \| Ubiquitin-protein ligase E3C OS=Homo sapiens GN=UBE3C PE=1 SV=3 - [UBE3C_HUMAN] \| \| ADP/ATP translocase 2 OS=Homo sapiens GN=SLC25A5 PE=1 SV=7 - [ADT2_HUMAN] \| \| Pre-mRNA-processing factor 6 OS=Homo sapiens GN=PRPF6 PE=1 SV=1 - [PRP6_HUMAN] \| \| Cytospin-B OS=Homo sapiens GN=SPECC1 PE=1 SV=1 - [CYTSB_HUMAN] \| \| Nodal modulator 3 OS=Homo sapiens GN=NOMO3 PE=2 SV=2 - [NOMO3_HUMAN] \| \| Elongation factor 1-alpha 1 OS=Homo sapiens GN=EEF1A1 PE=1 SV=1 - [EF1A1_HUMAN] \| \| ATP-dependent DNA helicase Q5 OS=Homo sapiens GN=RECQL5 PE=1 SV=2 - [RECQ5_HUMAN] \| \| Ankyrin-3 OS=Homo sapiens GN=ANK3 PE=1 SV=3 - [ANK3_HUMAN] \| \| DNA-directed RNA polymerase II subunit RPB2 OS=Homo sapiens GN=POLR2B PE=1 SV=1 - [RPB2_HUMAN] \| \|  \| |
| --- | --- | --- | --- | --- | --- | --- | --- | --- | --- | --- | --- | --- | --- | --- | --- | --- | --- | --- | --- | --- | --- | --- | --- | --- | --- | --- | --- | --- | --- | --- | --- | --- | --- | --- | --- | --- | --- | --- | --- | --- | --- | --- | --- | --- | --- | --- | --- | --- | --- | --- | --- | --- | --- | --- | --- | --- | --- | --- | --- | --- | --- | --- | --- | --- | --- | --- | --- | --- | --- | --- | --- | --- | --- | --- | --- | --- | --- | --- | --- | --- | --- | --- | --- | --- | --- | --- | --- | --- | --- | --- | --- | --- | --- | --- | --- | --- | --- | --- | --- | --- | --- | --- | --- | --- | --- | --- | --- | --- | --- | --- | --- | --- | --- | --- | --- | --- | --- | --- | --- | --- | --- | --- | --- | --- | --- | --- | --- | --- | --- | --- | --- | --- | --- | --- | --- | --- | --- | --- | --- | --- | --- | --- | --- | --- | --- | --- | --- | --- | --- | --- | --- | --- | --- | --- | --- | --- | --- | --- | --- | --- | --- | --- | --- | --- | --- | --- | --- | --- | --- | --- | --- | --- | --- | --- | --- | --- | --- | --- | --- | --- | --- | --- | --- | --- | --- | --- | --- | --- | --- | --- | --- | --- | --- | --- | --- | --- | --- | --- | --- | --- | --- | --- | --- | --- | --- | --- | --- | --- | --- | --- | --- | --- | --- | --- | --- | --- | --- | --- | --- | --- | --- | --- | --- | --- | --- | --- | --- | --- | --- | --- | --- | --- | --- | --- | --- | --- | --- | --- | --- | --- | --- | --- | --- | --- | --- | --- | --- | --- | --- | --- | --- | --- | --- | --- | --- | --- | --- | --- | --- | --- | --- | --- | --- | --- | --- | --- | --- | --- | --- | --- | --- | --- | --- | --- | --- | --- | --- | --- | --- | --- | --- | --- | --- | --- | --- | --- | --- | --- | --- | --- | --- | --- | --- | --- | --- | --- | --- | --- | --- | --- | --- | --- | --- | --- | --- | --- | --- | --- | --- | --- | --- | --- | --- | --- | --- | --- | --- | --- | --- | --- | --- | --- | --- | --- | --- | --- | --- | --- | --- | --- | --- | --- | --- | --- | --- | --- | --- | --- | --- | --- | --- | --- | --- | --- | --- | --- | --- | --- | --- | --- | --- | --- | --- | --- | --- | --- | --- | --- | --- | --- | --- | --- | --- | --- | --- | --- | --- | --- | --- | --- | --- | --- | --- | --- | --- | --- | --- | --- | --- | --- | --- | --- | --- | --- | --- | --- | --- | --- | --- | --- | --- | --- | --- | --- | --- | --- | --- | --- | --- | --- | --- | --- | --- | --- | --- | --- | --- | --- | --- | --- | --- | --- | --- | --- | --- | --- | --- | --- | --- | --- | --- | --- | --- | --- | --- | --- | --- | --- | --- | --- | --- | --- | --- | --- | --- | --- | --- | --- | --- | --- | --- | --- | --- | --- | --- | --- | --- | --- | --- | --- | --- | --- | --- | --- | --- | --- | --- | --- | --- | --- | --- | --- | --- | --- | --- | --- | --- | --- | --- | --- | --- | --- | --- | --- | --- | --- | --- | --- | --- | --- | --- | --- | --- | --- | --- | --- | --- | --- | --- | --- | --- | --- | --- | --- | --- | --- | --- | --- | --- | --- | --- | --- | --- | --- | --- | --- | --- | --- | --- | --- | --- | --- | --- | --- | --- | --- | --- | --- | --- | --- | --- | --- | --- | --- | --- | --- | --- | --- | --- | --- | --- | --- | --- | --- | --- | --- | --- | --- | --- | --- | --- | --- | --- | --- | --- | --- | --- | --- | --- | --- | --- | --- | --- | --- | --- | --- | --- | --- | --- | --- | --- | --- | --- | --- | --- | --- | --- | --- | --- | --- | --- | --- | --- | --- | --- | --- | --- | --- | --- | --- | --- | --- | --- | --- | --- | --- | --- | --- | --- | --- | --- | --- | --- | --- | --- | --- | --- | --- | --- | --- | --- | --- | --- | --- | --- | --- | --- | --- | --- | --- | --- | --- | --- | --- | --- | --- | --- | --- | --- | --- | --- | --- | --- | --- | --- | --- | --- | --- | --- | --- | --- | --- | --- | --- | --- | --- | --- | --- | --- | --- | --- | --- | --- | --- | --- | --- | --- | --- | --- | --- | --- | --- | --- | --- | --- | --- | --- | --- | --- | --- | --- | --- | --- | --- | --- | --- | --- | --- | --- | --- | --- | --- | --- | --- | --- | --- | --- | --- | --- | --- | --- | --- | --- | --- | --- | --- | --- | --- | --- | --- | --- | --- | --- | --- | --- | --- | --- | --- | --- | --- | --- | --- | --- | --- | --- | --- | --- | --- | --- | --- | --- | --- | --- | --- | --- | --- | --- | --- | --- | --- | --- | --- | --- | --- | --- | --- | --- | --- | --- | --- | --- | --- | --- | --- | --- | --- | --- | --- | --- | --- | --- | --- | --- | --- | --- | --- | --- | --- | --- | --- | --- | --- | --- | --- | --- | --- | --- | --- | --- | --- | --- | --- | --- | --- | --- | --- | --- | --- | --- | --- | --- | --- | --- | --- | --- | --- | --- | --- | --- | --- | --- | --- | --- | --- | --- | --- | --- | --- | --- | --- | --- | --- | --- | --- | --- | --- | --- | --- | --- | --- | --- | --- | --- | --- | --- | --- | --- | --- | --- | --- | --- | --- | --- | --- | --- | --- | --- | --- | --- | --- | --- | --- | --- | --- | --- | --- | --- | --- | --- | --- | --- | --- | --- | --- | --- | --- | --- | --- | --- | --- | --- | --- | --- | --- | --- | --- | --- | --- | --- | --- | --- | --- | --- | --- | --- | --- | --- | --- | --- | --- | --- | --- | --- | --- | --- | --- | --- | --- | --- | --- | --- | --- | --- | --- | --- | --- | --- | --- | --- | --- | --- | --- | --- | --- | --- | --- | --- | --- | --- | --- | --- | --- | --- | --- | --- | --- | --- | --- | --- | --- | --- | --- | --- | --- | --- | --- | --- | --- | --- | --- | --- | --- | --- | --- | --- | --- | --- | --- | --- | --- | --- | --- | --- | --- | --- | --- | --- | --- | --- | --- | --- | --- | --- | --- | --- | --- | --- | --- | --- | --- | --- | --- | --- | --- | --- | --- | --- | --- | --- | --- | --- | --- | --- | --- | --- | --- | --- | --- | --- | --- | --- | --- | --- | --- | --- | --- | --- | --- | --- | --- | --- | --- | --- | --- | --- | --- | --- | --- | --- | --- | --- | --- | --- | --- | --- | --- | --- | --- | --- | --- | --- | --- | --- | --- | --- | --- | --- | --- | --- | --- | --- | --- | --- | --- | --- | --- | --- | --- | --- | --- | --- | --- | --- | --- | --- | --- | --- | --- | --- | --- | --- | --- | --- | --- | --- | --- | --- | --- | --- | --- | --- | --- | --- | --- | --- | --- | --- | --- | --- | --- | --- | --- | --- | --- | --- | --- | --- | --- | --- | --- | --- | --- | --- | --- | --- | --- | --- | --- | --- | --- | --- | --- | --- | --- | --- | --- | --- | --- | --- | --- | --- | --- | --- | --- | --- | --- | --- | --- | --- | --- | --- | --- | --- | --- | --- | --- | --- | --- | --- | --- | --- | --- | --- | --- | --- | --- | --- | --- | --- | --- | --- | --- | --- | --- | --- | --- | --- | --- | --- | --- | --- | --- | --- | --- | --- | --- | --- | --- | --- | --- | --- | --- | --- | --- | --- | --- | --- | --- | --- | --- | --- | --- | --- | --- | --- | --- | --- | --- | --- | --- | --- | --- | --- | --- | --- | --- | --- | --- | --- | --- | --- | --- | --- | --- | --- | --- | --- | --- | --- | --- |

**Sample S2:**

| \| Accession \| \| --- \| \| O43795 \| \| O95071 \| \| Q86VP6 \| \| P28340 \| \| Q92900 \| \| Q9BZH6 \| \| Q7L2E3 \| \| Q9P2J5 \| \| Q8WUM0 \| \| P30876 \| \| O15205 \| \| P09874 \| \| P0CG48 \| \| Q08211 \| \| P58107 \| \| Q7L014 \| \| Q9HAV4 \| \| Q96KR1 \| \| Q15393 \| \| O14974 \| \| Q14152 \| \| P15924 \| \| P52701 \| \| P78347 \| \| O60264 \| \| Q8IUD2 \| \| Q96SB8 \| \| O00203 \| \| Q9NZB2 \| \| Q9UPP1 \| \| O15042 \| \| P42285 \| \| Q86XI2 \| \| Q6WCQ1 \| \| P29597 \| \| Q15149 \| \| O00159 \| \| O00411 \| \| O15083 \| \| P57737 \| \| P52732 \| \| Q93009 \| \| Q9P0K7 \| \| Q8TEW0 \| \| O60231 \| \| Q08379 \| \| Q9NW08 \| \| O75122 \| \| Q8IWC1 \| \| P51610 \| \| O75694 \| \| Q96N67 \| \| Q8WVS4 \| \| Q96Q05 \| \| P22314 \| \| Q3KQU3 \| \| P53396 \| \| O14980 \| \| Q96F07 \| \| O75150 \| \| Q8IY18 \| \| Q14157 \| \| Q14147 \| \| Q7Z460 \| \| P20585 \| \| Q7L576 \| \| Q16531 \| \| Q13523 \| \| P53621 \| \| P46379 \| \| Q86W56 \| \| Q9UM54 \| \| Q659C4 \| \| Q69YQ0 \| \| P78527 \| \| Q2M389 \| \| P69849 \| \| Q8N3U4 \| \| Q70EL4 \| \| Q10570 \| \| Q13813 \| \| Q8IXT5 \| \| O75533 \| \| Q9Y6X9 \| \| P23458 \| \| Q8IVF7 \| \| Q12965 \| \| Q15424 \| \| Q99569 \| \| O95163 \| \| Q9NQW6 \| \| Q504Q3 \| \| Q6P1N0 \| \| Q5M775 \| \| Q13751 \| \| P35580 \| \| O60879 \| \| Q14527 \| \| Q99613 \| \| Q8TF46 \| \| Q14562 \| \| O75044 \| \| Q9H6S0 \| \| Q9BSJ8 \| \| Q99575 \| \| Q14145 \| \| O75717 \| \| Q86V48 \| \| Q5SVZ6 \| \| Q99707 \| \| Q14151 \| \| P18206 \| \| Q8IX01 \| \| O60282 \| \| Q9NSV4 \| \| Q6ZRQ5 \| \| Q9BQE3 \| \| P53992 \| \| P57740 \| \| Q12769 \| \| Q92540 \| \| P41252 \| \| Q9HCE1 \| \| P05141 \| \|  \| | \| Coverage \| \| --- \| \| 23.94 \| \| 11.00 \| \| 15.69 \| \| 14.81 \| \| 16.21 \| \| 12.99 \| \| 13.48 \| \| 12.93 \| \| 9.78 \| \| 15.84 \| \| 29.09 \| \| 4.04 \| \| 52.55 \| \| 8.19 \| \| 14.93 \| \| 11.25 \| \| 11.21 \| \| 9.12 \| \| 7.72 \| \| 7.57 \| \| 7.38 \| \| 4.70 \| \| 7.50 \| \| 10.22 \| \| 3.99 \| \| 5.65 \| \| 9.07 \| \| 6.49 \| \| 9.21 \| \| 5.75 \| \| 6.03 \| \| 7.77 \| \| 4.90 \| \| 9.37 \| \| 5.39 \| \| 3.16 \| \| 7.43 \| \| 5.61 \| \| 4.08 \| \| 3.03 \| \| 6.25 \| \| 8.71 \| \| 5.71 \| \| 1.55 \| \| 7.97 \| \| 3.39 \| \| 4.15 \| \| 3.55 \| \| 3.20 \| \| 2.51 \| \| 3.81 \| \| 2.71 \| \| 4.97 \| \| 5.57 \| \| 5.29 \| \| 2.97 \| \| 5.81 \| \| 3.83 \| \| 6.42 \| \| 5.00 \| \| 5.72 \| \| 3.04 \| \| 2.97 \| \| 3.51 \| \| 3.34 \| \| 6.94 \| \| 5.00 \| \| 3.77 \| \| 4.25 \| \| 4.15 \| \| 4.20 \| \| 2.70 \| \| 2.41 \| \| 3.76 \| \| 0.94 \| \| 3.24 \| \| 3.19 \| \| 2.27 \| \| 4.36 \| \| 2.15 \| \| 1.42 \| \| 2.30 \| \| 1.61 \| \| 1.94 \| \| 1.99 \| \| 2.43 \| \| 2.08 \| \| 2.40 \| \| 2.77 \| \| 1.58 \| \| 2.67 \| \| 2.16 \| \| 2.21 \| \| 2.43 \| \| 1.96 \| \| 1.42 \| \| 2.09 \| \| 4.16 \| \| 2.08 \| \| 2.09 \| \| 1.72 \| \| 1.77 \| \| 3.15 \| \| 3.80 \| \| 2.25 \| \| 4.97 \| \| 1.77 \| \| 3.35 \| \| 1.75 \| \| 1.98 \| \| 2.31 \| \| 2.73 \| \| 2.03 \| \| 2.19 \| \| 1.84 \| \| 2.41 \| \| 4.23 \| \| 1.46 \| \| 2.16 \| \| 1.39 \| \| 1.93 \| \| 1.43 \| \| 1.99 \| \| 5.70 \| \|  \| | \| # PSMs \| \| --- \| \| 64 \| \| 39 \| \| 27 \| \| 31 \| \| 33 \| \| 28 \| \| 28 \| \| 21 \| \| 17 \| \| 28 \| \| 16 \| \| 8 \| \| 21 \| \| 16 \| \| 18 \| \| 17 \| \| 19 \| \| 15 \| \| 14 \| \| 12 \| \| 16 \| \| 17 \| \| 12 \| \| 17 \| \| 13 \| \| 10 \| \| 12 \| \| 10 \| \| 13 \| \| 9 \| \| 7 \| \| 14 \| \| 9 \| \| 12 \| \| 8 \| \| 17 \| \| 12 \| \| 8 \| \| 7 \| \| 4 \| \| 9 \| \| 11 \| \| 9 \| \| 5 \| \| 12 \| \| 7 \| \| 7 \| \| 7 \| \| 5 \| \| 7 \| \| 7 \| \| 9 \| \| 8 \| \| 7 \| \| 7 \| \| 3 \| \| 10 \| \| 5 \| \| 13 \| \| 8 \| \| 8 \| \| 4 \| \| 7 \| \| 8 \| \| 4 \| \| 11 \| \| 5 \| \| 5 \| \| 8 \| \| 8 \| \| 5 \| \| 5 \| \| 3 \| \| 6 \| \| 5 \| \| 5 \| \| 4 \| \| 3 \| \| 6 \| \| 5 \| \| 5 \| \| 3 \| \| 5 \| \| 3 \| \| 3 \| \| 4 \| \| 4 \| \| 3 \| \| 5 \| \| 3 \| \| 3 \| \| 5 \| \| 5 \| \| 4 \| \| 4 \| \| 6 \| \| 3 \| \| 4 \| \| 3 \| \| 3 \| \| 4 \| \| 3 \| \| 4 \| \| 4 \| \| 4 \| \| 5 \| \| 3 \| \| 3 \| \| 2 \| \| 2 \| \| 2 \| \| 7 \| \| 3 \| \| 3 \| \| 2 \| \| 3 \| \| 2 \| \| 3 \| \| 3 \| \| 3 \| \| 2 \| \| 2 \| \| 2 \| \| 2 \| \|  \| | \| # Peptides \| \| --- \| \| 22 \| \| 22 \| \| 15 \| \| 14 \| \| 16 \| \| 12 \| \| 14 \| \| 11 \| \| 9 \| \| 16 \| \| 4 \| \| 3 \| \| 4 \| \| 8 \| \| 9 \| \| 10 \| \| 10 \| \| 7 \| \| 7 \| \| 6 \| \| 9 \| \| 11 \| \| 9 \| \| 10 \| \| 5 \| \| 6 \| \| 8 \| \| 7 \| \| 8 \| \| 5 \| \| 5 \| \| 7 \| \| 5 \| \| 7 \| \| 5 \| \| 14 \| \| 6 \| \| 5 \| \| 4 \| \| 2 \| \| 5 \| \| 7 \| \| 5 \| \| 2 \| \| 7 \| \| 3 \| \| 4 \| \| 4 \| \| 3 \| \| 4 \| \| 4 \| \| 5 \| \| 4 \| \| 5 \| \| 4 \| \| 2 \| \| 5 \| \| 3 \| \| 8 \| \| 4 \| \| 6 \| \| 2 \| \| 3 \| \| 5 \| \| 3 \| \| 8 \| \| 4 \| \| 3 \| \| 5 \| \| 4 \| \| 3 \| \| 3 \| \| 2 \| \| 4 \| \| 3 \| \| 3 \| \| 3 \| \| 2 \| \| 4 \| \| 3 \| \| 3 \| \| 2 \| \| 2 \| \| 2 \| \| 2 \| \| 2 \| \| 2 \| \| 2 \| \| 3 \| \| 2 \| \| 2 \| \| 3 \| \| 2 \| \| 2 \| \| 2 \| \| 3 \| \| 2 \| \| 3 \| \| 2 \| \| 2 \| \| 2 \| \| 2 \| \| 4 \| \| 3 \| \| 2 \| \| 3 \| \| 2 \| \| 3 \| \| 2 \| \| 2 \| \| 2 \| \| 3 \| \| 2 \| \| 2 \| \| 2 \| \| 3 \| \| 2 \| \| 2 \| \| 2 \| \| 2 \| \| 2 \| \| 2 \| \| 2 \| \| 2 \| \|  \| | \| # AAs \| \| --- \| \| 1136 \| \| 2799 \| \| 1230 \| \| 1107 \| \| 1129 \| \| 1224 \| \| 1194 \| \| 1176 \| \| 1156 \| \| 1174 \| \| 165 \| \| 1014 \| \| 685 \| \| 1270 \| \| 5090 \| \| 1031 \| \| 1204 \| \| 1074 \| \| 1217 \| \| 1030 \| \| 1382 \| \| 2871 \| \| 1360 \| \| 998 \| \| 1052 \| \| 1116 \| \| 1091 \| \| 1094 \| \| 1118 \| \| 1060 \| \| 1029 \| \| 1042 \| \| 1143 \| \| 1025 \| \| 1187 \| \| 4684 \| \| 1063 \| \| 1230 \| \| 957 \| \| 925 \| \| 1056 \| \| 1102 \| \| 980 \| \| 1356 \| \| 1041 \| \| 1002 \| \| 1133 \| \| 1294 \| \| 876 \| \| 2035 \| \| 1391 \| \| 2140 \| \| 1066 \| \| 1148 \| \| 1058 \| \| 841 \| \| 1101 \| \| 1071 \| \| 1278 \| \| 1001 \| \| 1101 \| \| 1087 \| \| 1143 \| \| 1538 \| \| 1137 \| \| 1253 \| \| 1140 \| \| 1007 \| \| 1224 \| \| 1132 \| \| 976 \| \| 1294 \| \| 914 \| \| 1117 \| \| 4128 \| \| 1173 \| \| 1222 \| \| 1231 \| \| 1123 \| \| 1443 \| \| 2472 \| \| 1001 \| \| 1304 \| \| 1032 \| \| 1154 \| \| 1028 \| \| 1108 \| \| 915 \| \| 1192 \| \| 1332 \| \| 1124 \| \| 1202 \| \| 951 \| \| 1068 \| \| 1172 \| \| 1976 \| \| 1101 \| \| 1009 \| \| 913 \| \| 1054 \| \| 1220 \| \| 1071 \| \| 1430 \| \| 1104 \| \| 1024 \| \| 624 \| \| 1129 \| \| 1076 \| \| 1142 \| \| 1265 \| \| 953 \| \| 1134 \| \| 1082 \| \| 957 \| \| 1193 \| \| 1243 \| \| 449 \| \| 1094 \| \| 925 \| \| 1436 \| \| 1137 \| \| 1262 \| \| 1003 \| \| 298 \| \|  \| | \| MW [kDa] \| \| --- \| \| 131.9 \| \| 309.2 \| \| 136.3 \| \| 123.6 \| \| 124.3 \| \| 136.6 \| \| 133.9 \| \| 134.4 \| \| 128.9 \| \| 133.8 \| \| 18.5 \| \| 113.0 \| \| 77.0 \| \| 140.9 \| \| 555.3 \| \| 117.3 \| \| 136.2 \| \| 116.9 \| \| 135.5 \| \| 115.2 \| \| 166.5 \| \| 331.6 \| \| 152.7 \| \| 112.3 \| \| 121.8 \| \| 128.0 \| \| 126.2 \| \| 121.2 \| \| 121.8 \| \| 117.8 \| \| 118.2 \| \| 117.7 \| \| 130.9 \| \| 116.5 \| \| 133.6 \| \| 531.5 \| \| 121.6 \| \| 138.5 \| \| 110.5 \| \| 100.5 \| \| 119.1 \| \| 128.2 \| \| 110.0 \| \| 151.3 \| \| 119.2 \| \| 113.0 \| \| 127.7 \| \| 141.0 \| \| 98.4 \| \| 208.6 \| \| 155.1 \| \| 242.4 \| \| 122.5 \| \| 128.4 \| \| 117.8 \| \| 92.8 \| \| 120.8 \| \| 123.3 \| \| 148.3 \| \| 113.6 \| \| 128.7 \| \| 114.5 \| \| 128.0 \| \| 169.3 \| \| 127.3 \| \| 145.1 \| \| 126.9 \| \| 116.9 \| \| 138.3 \| \| 119.3 \| \| 111.0 \| \| 149.6 \| \| 105.3 \| \| 124.5 \| \| 468.8 \| \| 136.3 \| \| 134.0 \| \| 141.2 \| \| 122.7 \| \| 160.8 \| \| 284.4 \| \| 118.0 \| \| 145.7 \| \| 117.7 \| \| 133.2 \| \| 117.1 \| \| 127.0 \| \| 102.6 \| \| 131.8 \| \| 150.2 \| \| 124.1 \| \| 135.3 \| \| 104.0 \| \| 118.5 \| \| 129.5 \| \| 228.9 \| \| 125.5 \| \| 113.9 \| \| 105.3 \| \| 120.7 \| \| 139.2 \| \| 120.8 \| \| 160.1 \| \| 122.8 \| \| 114.6 \| \| 69.6 \| \| 125.9 \| \| 120.2 \| \| 128.6 \| \| 140.4 \| \| 107.4 \| \| 123.7 \| \| 120.1 \| \| 109.4 \| \| 136.8 \| \| 142.2 \| \| 49.9 \| \| 118.2 \| \| 106.3 \| \| 162.0 \| \| 127.2 \| \| 144.4 \| \| 113.6 \| \| 32.8 \| \|  \| | \| calc. pI \| \| --- \| \| 9.38 \| \| 5.85 \| \| 5.78 \| \| 7.03 \| \| 6.61 \| \| 6.92 \| \| 8.78 \| \| 7.30 \| \| 5.10 \| \| 6.87 \| \| 8.90 \| \| 8.88 \| \| 7.66 \| \| 6.84 \| \| 5.60 \| \| 9.29 \| \| 5.80 \| \| 9.04 \| \| 5.26 \| \| 5.40 \| \| 6.79 \| \| 6.81 \| \| 6.90 \| \| 6.39 \| \| 8.09 \| \| 5.97 \| \| 6.99 \| \| 6.04 \| \| 8.88 \| \| 8.72 \| \| 8.47 \| \| 6.52 \| \| 6.87 \| \| 6.21 \| \| 7.15 \| \| 5.96 \| \| 9.41 \| \| 8.98 \| \| 6.99 \| \| 5.80 \| \| 5.64 \| \| 5.55 \| \| 6.21 \| \| 7.68 \| \| 6.80 \| \| 5.02 \| \| 8.50 \| \| 8.47 \| \| 9.32 \| \| 7.46 \| \| 6.16 \| \| 6.80 \| \| 7.31 \| \| 6.62 \| \| 5.76 \| \| 10.11 \| \| 7.33 \| \| 6.06 \| \| 7.31 \| \| 6.23 \| \| 8.38 \| \| 7.11 \| \| 7.56 \| \| 9.03 \| \| 8.02 \| \| 6.90 \| \| 5.26 \| \| 10.26 \| \| 7.66 \| \| 5.60 \| \| 6.43 \| \| 8.53 \| \| 7.61 \| \| 5.72 \| \| 7.12 \| \| 7.44 \| \| 5.67 \| \| 5.43 \| \| 9.19 \| \| 6.40 \| \| 5.35 \| \| 6.81 \| \| 7.09 \| \| 8.38 \| \| 7.55 \| \| 6.65 \| \| 8.92 \| \| 5.47 \| \| 8.94 \| \| 5.94 \| \| 8.07 \| \| 5.99 \| \| 8.09 \| \| 6.70 \| \| 7.21 \| \| 5.54 \| \| 6.58 \| \| 8.60 \| \| 5.68 \| \| 6.54 \| \| 8.32 \| \| 6.70 \| \| 8.40 \| \| 5.83 \| \| 9.22 \| \| 6.44 \| \| 5.62 \| \| 8.50 \| \| 7.44 \| \| 5.58 \| \| 6.16 \| \| 5.66 \| \| 7.28 \| \| 6.19 \| \| 7.03 \| \| 7.12 \| \| 5.10 \| \| 7.06 \| \| 5.43 \| \| 5.50 \| \| 8.72 \| \| 6.15 \| \| 8.82 \| \| 9.69 \| \|  \| | \| Score \| \| --- \| \| 1322.86 \| \| 711.68 \| \| 677.49 \| \| 608.15 \| \| 560.24 \| \| 526.92 \| \| 446.32 \| \| 423.29 \| \| 409.83 \| \| 392.36 \| \| 388.82 \| \| 365.52 \| \| 348.98 \| \| 341.13 \| \| 334.25 \| \| 329.28 \| \| 313.48 \| \| 306.37 \| \| 279.98 \| \| 267.31 \| \| 235.30 \| \| 230.25 \| \| 229.56 \| \| 225.95 \| \| 222.38 \| \| 221.49 \| \| 200.69 \| \| 195.35 \| \| 179.40 \| \| 177.70 \| \| 173.52 \| \| 173.42 \| \| 169.27 \| \| 168.83 \| \| 163.96 \| \| 157.22 \| \| 156.76 \| \| 156.32 \| \| 154.46 \| \| 141.55 \| \| 139.80 \| \| 136.97 \| \| 135.53 \| \| 132.63 \| \| 132.24 \| \| 131.85 \| \| 129.86 \| \| 126.18 \| \| 123.60 \| \| 117.78 \| \| 117.67 \| \| 116.51 \| \| 113.95 \| \| 113.81 \| \| 112.68 \| \| 109.48 \| \| 108.69 \| \| 108.66 \| \| 104.91 \| \| 103.27 \| \| 102.48 \| \| 100.97 \| \| 100.94 \| \| 99.08 \| \| 96.95 \| \| 96.62 \| \| 96.56 \| \| 95.91 \| \| 95.11 \| \| 92.41 \| \| 91.78 \| \| 91.19 \| \| 90.91 \| \| 81.76 \| \| 81.05 \| \| 79.99 \| \| 78.38 \| \| 74.36 \| \| 74.11 \| \| 72.57 \| \| 72.52 \| \| 71.38 \| \| 71.05 \| \| 70.65 \| \| 70.19 \| \| 67.31 \| \| 66.98 \| \| 66.50 \| \| 61.90 \| \| 61.45 \| \| 55.15 \| \| 54.51 \| \| 53.76 \| \| 53.14 \| \| 52.44 \| \| 51.22 \| \| 50.87 \| \| 49.51 \| \| 48.93 \| \| 48.79 \| \| 48.20 \| \| 48.04 \| \| 47.43 \| \| 44.59 \| \| 44.43 \| \| 41.24 \| \| 40.76 \| \| 40.73 \| \| 40.00 \| \| 39.02 \| \| 38.94 \| \| 38.32 \| \| 36.24 \| \| 35.40 \| \| 34.88 \| \| 33.84 \| \| 30.13 \| \| 30.02 \| \| 29.11 \| \| 28.23 \| \| 27.46 \| \| 26.59 \| \| 24.34 \| \| 23.02 \| \|  \| | \| Description \| \| --- \| \| Unconventional myosin-Ib OS=Homo sapiens GN=MYO1B PE=2 SV=3 - [MYO1B_HUMAN] \| \| E3 ubiquitin-protein ligase UBR5 OS=Homo sapiens GN=UBR5 PE=1 SV=2 - [UBR5_HUMAN] \| \| Cullin-associated NEDD8-dissociated protein 1 OS=Homo sapiens GN=CAND1 PE=1 SV=2 - [CAND1_HUMAN] \| \| DNA polymerase delta catalytic subunit OS=Homo sapiens GN=POLD1 PE=1 SV=2 - [DPOD1_HUMAN] \| \| Regulator of nonsense transcripts 1 OS=Homo sapiens GN=UPF1 PE=1 SV=2 - [RENT1_HUMAN] \| \| WD repeat-containing protein 11 OS=Homo sapiens GN=WDR11 PE=1 SV=1 - [WDR11_HUMAN] \| \| Putative ATP-dependent RNA helicase DHX30 OS=Homo sapiens GN=DHX30 PE=1 SV=1 - [DHX30_HUMAN] \| \| Leucine--tRNA ligase, cytoplasmic OS=Homo sapiens GN=LARS PE=1 SV=2 - [SYLC_HUMAN] \| \| Nuclear pore complex protein Nup133 OS=Homo sapiens GN=NUP133 PE=1 SV=2 - [NU133_HUMAN] \| \| DNA-directed RNA polymerase II subunit RPB2 OS=Homo sapiens GN=POLR2B PE=1 SV=1 - [RPB2_HUMAN] \| \| Ubiquitin D OS=Homo sapiens GN=UBD PE=1 SV=2 - [UBD_HUMAN] \| \| Poly [ADP-ribose] polymerase 1 OS=Homo sapiens GN=PARP1 PE=1 SV=4 - [PARP1_HUMAN] \| \| Polyubiquitin-C OS=Homo sapiens GN=UBC PE=1 SV=3 - [UBC_HUMAN] \| \| ATP-dependent RNA helicase A OS=Homo sapiens GN=DHX9 PE=1 SV=4 - [DHX9_HUMAN] \| \| Epiplakin OS=Homo sapiens GN=EPPK1 PE=1 SV=2 - [EPIPL_HUMAN] \| \| Probable ATP-dependent RNA helicase DDX46 OS=Homo sapiens GN=DDX46 PE=1 SV=2 - [DDX46_HUMAN] \| \| Exportin-5 OS=Homo sapiens GN=XPO5 PE=1 SV=1 - [XPO5_HUMAN] \| \| Zinc finger RNA-binding protein OS=Homo sapiens GN=ZFR PE=1 SV=2 - [ZFR_HUMAN] \| \| Splicing factor 3B subunit 3 OS=Homo sapiens GN=SF3B3 PE=1 SV=4 - [SF3B3_HUMAN] \| \| Protein phosphatase 1 regulatory subunit 12A OS=Homo sapiens GN=PPP1R12A PE=1 SV=1 - [MYPT1_HUMAN] \| \| Eukaryotic translation initiation factor 3 subunit A OS=Homo sapiens GN=EIF3A PE=1 SV=1 - [EIF3A_HUMAN] \| \| Desmoplakin OS=Homo sapiens GN=DSP PE=1 SV=3 - [DESP_HUMAN] \| \| DNA mismatch repair protein Msh6 OS=Homo sapiens GN=MSH6 PE=1 SV=2 - [MSH6_HUMAN] \| \| General transcription factor II-I OS=Homo sapiens GN=GTF2I PE=1 SV=2 - [GTF2I_HUMAN] \| \| SWI/SNF-related matrix-associated actin-dependent regulator of chromatin subfamily A member 5 OS=Homo sapiens GN=SMARCA5 PE=1 SV=1 - [SMCA5_HUMAN] \| \| ELKS/Rab6-interacting/CAST family member 1 OS=Homo sapiens GN=ERC1 PE=1 SV=1 - [RB6I2_HUMAN] \| \| Structural maintenance of chromosomes protein 6 OS=Homo sapiens GN=SMC6 PE=1 SV=2 - [SMC6_HUMAN] \| \| AP-3 complex subunit beta-1 OS=Homo sapiens GN=AP3B1 PE=1 SV=3 - [AP3B1_HUMAN] \| \| Constitutive coactivator of PPAR-gamma-like protein 1 OS=Homo sapiens GN=FAM120A PE=1 SV=2 - [F120A_HUMAN] \| \| Histone lysine demethylase PHF8 OS=Homo sapiens GN=PHF8 PE=1 SV=3 - [PHF8_HUMAN] \| \| U2 snRNP-associated SURP motif-containing protein OS=Homo sapiens GN=U2SURP PE=1 SV=2 - [SR140_HUMAN] \| \| Superkiller viralicidic activity 2-like 2 OS=Homo sapiens GN=SKIV2L2 PE=1 SV=3 - [SK2L2_HUMAN] \| \| Condensin-2 complex subunit G2 OS=Homo sapiens GN=NCAPG2 PE=1 SV=1 - [CNDG2_HUMAN] \| \| Myosin phosphatase Rho-interacting protein OS=Homo sapiens GN=MPRIP PE=1 SV=3 - [MPRIP_HUMAN] \| \| Non-receptor tyrosine-protein kinase TYK2 OS=Homo sapiens GN=TYK2 PE=1 SV=3 - [TYK2_HUMAN] \| \| Plectin OS=Homo sapiens GN=PLEC PE=1 SV=3 - [PLEC_HUMAN] \| \| Unconventional myosin-Ic OS=Homo sapiens GN=MYO1C PE=1 SV=4 - [MYO1C_HUMAN] \| \| DNA-directed RNA polymerase, mitochondrial OS=Homo sapiens GN=POLRMT PE=1 SV=2 - [RPOM_HUMAN] \| \| ERC protein 2 OS=Homo sapiens GN=ERC2 PE=1 SV=3 - [ERC2_HUMAN] \| \| Coronin-7 OS=Homo sapiens GN=CORO7 PE=1 SV=2 - [CORO7_HUMAN] \| \| Kinesin-like protein KIF11 OS=Homo sapiens GN=KIF11 PE=1 SV=2 - [KIF11_HUMAN] \| \| Ubiquitin carboxyl-terminal hydrolase 7 OS=Homo sapiens GN=USP7 PE=1 SV=2 - [UBP7_HUMAN] \| \| Ankycorbin OS=Homo sapiens GN=RAI14 PE=1 SV=2 - [RAI14_HUMAN] \| \| Partitioning defective 3 homolog OS=Homo sapiens GN=PARD3 PE=1 SV=2 - [PARD3_HUMAN] \| \| Putative pre-mRNA-splicing factor ATP-dependent RNA helicase DHX16 OS=Homo sapiens GN=DHX16 PE=1 SV=2 - [DHX16_HUMAN] \| \| Golgin subfamily A member 2 OS=Homo sapiens GN=GOLGA2 PE=1 SV=3 - [GOGA2_HUMAN] \| \| DNA-directed RNA polymerase III subunit RPC2 OS=Homo sapiens GN=POLR3B PE=1 SV=2 - [RPC2_HUMAN] \| \| CLIP-associating protein 2 OS=Homo sapiens GN=CLASP2 PE=1 SV=2 - [CLAP2_HUMAN] \| \| MAP7 domain-containing protein 3 OS=Homo sapiens GN=MAP7D3 PE=1 SV=2 - [MA7D3_HUMAN] \| \| Host cell factor 1 OS=Homo sapiens GN=HCFC1 PE=1 SV=2 - [HCFC1_HUMAN] \| \| Nuclear pore complex protein Nup155 OS=Homo sapiens GN=NUP155 PE=1 SV=1 - [NU155_HUMAN] \| \| Dedicator of cytokinesis protein 7 OS=Homo sapiens GN=DOCK7 PE=1 SV=4 - [DOCK7_HUMAN] \| \| WD repeat-containing protein 60 OS=Homo sapiens GN=WDR60 PE=1 SV=3 - [WDR60_HUMAN] \| \| Trafficking protein particle complex subunit 9 OS=Homo sapiens GN=TRAPPC9 PE=1 SV=2 - [TPPC9_HUMAN] \| \| Ubiquitin-like modifier-activating enzyme 1 OS=Homo sapiens GN=UBA1 PE=1 SV=3 - [UBA1_HUMAN] \| \| MAP7 domain-containing protein 1 OS=Homo sapiens GN=MAP7D1 PE=1 SV=1 - [MA7D1_HUMAN] \| \| ATP-citrate synthase OS=Homo sapiens GN=ACLY PE=1 SV=3 - [ACLY_HUMAN] \| \| Exportin-1 OS=Homo sapiens GN=XPO1 PE=1 SV=1 - [XPO1_HUMAN] \| \| Cytoplasmic FMR1-interacting protein 2 OS=Homo sapiens GN=CYFIP2 PE=1 SV=2 - [CYFP2_HUMAN] \| \| E3 ubiquitin-protein ligase BRE1B OS=Homo sapiens GN=RNF40 PE=1 SV=4 - [BRE1B_HUMAN] \| \| Structural maintenance of chromosomes protein 5 OS=Homo sapiens GN=SMC5 PE=1 SV=2 - [SMC5_HUMAN] \| \| Ubiquitin-associated protein 2-like OS=Homo sapiens GN=UBAP2L PE=1 SV=2 - [UBP2L_HUMAN] \| \| Probable ATP-dependent RNA helicase DHX34 OS=Homo sapiens GN=DHX34 PE=2 SV=2 - [DHX34_HUMAN] \| \| CLIP-associating protein 1 OS=Homo sapiens GN=CLASP1 PE=1 SV=1 - [CLAP1_HUMAN] \| \| DNA mismatch repair protein Msh3 OS=Homo sapiens GN=MSH3 PE=1 SV=4 - [MSH3_HUMAN] \| \| Cytoplasmic FMR1-interacting protein 1 OS=Homo sapiens GN=CYFIP1 PE=1 SV=1 - [CYFP1_HUMAN] \| \| DNA damage-binding protein 1 OS=Homo sapiens GN=DDB1 PE=1 SV=1 - [DDB1_HUMAN] \| \| Serine/threonine-protein kinase PRP4 homolog OS=Homo sapiens GN=PRPF4B PE=1 SV=3 - [PRP4B_HUMAN] \| \| Coatomer subunit alpha OS=Homo sapiens GN=COPA PE=1 SV=2 - [COPA_HUMAN] \| \| Large proline-rich protein BAG6 OS=Homo sapiens GN=BAG6 PE=1 SV=2 - [BAG6_HUMAN] \| \| Poly(ADP-ribose) glycohydrolase OS=Homo sapiens GN=PARG PE=1 SV=1 - [PARG_HUMAN] \| \| Unconventional myosin-VI OS=Homo sapiens GN=MYO6 PE=1 SV=4 - [MYO6_HUMAN] \| \| La-related protein 1B OS=Homo sapiens GN=LARP1B PE=1 SV=2 - [LAR1B_HUMAN] \| \| Cytospin-A OS=Homo sapiens GN=SPECC1L PE=1 SV=2 - [CYTSA_HUMAN] \| \| DNA-dependent protein kinase catalytic subunit OS=Homo sapiens GN=PRKDC PE=1 SV=3 - [PRKDC_HUMAN] \| \| WASH complex subunit 7 OS=Homo sapiens GN=KIAA1033 PE=1 SV=2 - [WASH7_HUMAN] \| \| Nodal modulator 3 OS=Homo sapiens GN=NOMO3 PE=2 SV=2 - [NOMO3_HUMAN] \| \| Cohesin subunit SA-2 OS=Homo sapiens GN=STAG2 PE=1 SV=3 - [STAG2_HUMAN] \| \| Ubiquitin carboxyl-terminal hydrolase 43 OS=Homo sapiens GN=USP43 PE=1 SV=2 - [UBP43_HUMAN] \| \| Cleavage and polyadenylation specificity factor subunit 1 OS=Homo sapiens GN=CPSF1 PE=1 SV=2 - [CPSF1_HUMAN] \| \| Spectrin alpha chain, brain OS=Homo sapiens GN=SPTAN1 PE=1 SV=3 - [SPTA2_HUMAN] \| \| RNA-binding protein 12B OS=Homo sapiens GN=RBM12B PE=1 SV=2 - [RB12B_HUMAN] \| \| Splicing factor 3B subunit 1 OS=Homo sapiens GN=SF3B1 PE=1 SV=3 - [SF3B1_HUMAN] \| \| MORC family CW-type zinc finger protein 2 OS=Homo sapiens GN=MORC2 PE=1 SV=2 - [MORC2_HUMAN] \| \| Tyrosine-protein kinase JAK1 OS=Homo sapiens GN=JAK1 PE=1 SV=2 - [JAK1_HUMAN] \| \| Formin-like protein 3 OS=Homo sapiens GN=FMNL3 PE=1 SV=3 - [FMNL3_HUMAN] \| \| Unconventional myosin-Ie OS=Homo sapiens GN=MYO1E PE=1 SV=2 - [MYO1E_HUMAN] \| \| Scaffold attachment factor B1 OS=Homo sapiens GN=SAFB PE=1 SV=4 - [SAFB1_HUMAN] \| \| Plakophilin-4 OS=Homo sapiens GN=PKP4 PE=1 SV=2 - [PKP4_HUMAN] \| \| Elongator complex protein 1 OS=Homo sapiens GN=IKBKAP PE=1 SV=3 - [ELP1_HUMAN] \| \| Actin-binding protein anillin OS=Homo sapiens GN=ANLN PE=1 SV=2 - [ANLN_HUMAN] \| \| PAB-dependent poly(A)-specific ribonuclease subunit 2 OS=Homo sapiens GN=PAN2 PE=1 SV=3 - [PAN2_HUMAN] \| \| Coiled-coil and C2 domain-containing protein 1A OS=Homo sapiens GN=CC2D1A PE=1 SV=1 - [C2D1A_HUMAN] \| \| Cytospin-B OS=Homo sapiens GN=SPECC1 PE=1 SV=1 - [CYTSB_HUMAN] \| \| Laminin subunit beta-3 OS=Homo sapiens GN=LAMB3 PE=1 SV=1 - [LAMB3_HUMAN] \| \| Myosin-10 OS=Homo sapiens GN=MYH10 PE=1 SV=3 - [MYH10_HUMAN] \| \| Protein diaphanous homolog 2 OS=Homo sapiens GN=DIAPH2 PE=1 SV=1 - [DIAP2_HUMAN] \| \| Helicase-like transcription factor OS=Homo sapiens GN=HLTF PE=1 SV=2 - [HLTF_HUMAN] \| \| Eukaryotic translation initiation factor 3 subunit C OS=Homo sapiens GN=EIF3C PE=1 SV=1 - [EIF3C_HUMAN] \| \| DIS3-like exonuclease 1 OS=Homo sapiens GN=DIS3L PE=1 SV=2 - [DI3L1_HUMAN] \| \| ATP-dependent RNA helicase DHX8 OS=Homo sapiens GN=DHX8 PE=1 SV=1 - [DHX8_HUMAN] \| \| SLIT-ROBO Rho GTPase-activating protein 2 OS=Homo sapiens GN=SRGAP2 PE=1 SV=2 - [FNBP2_HUMAN] \| \| Probable ATP-dependent RNA helicase YTHDC2 OS=Homo sapiens GN=YTHDC2 PE=1 SV=2 - [YTDC2_HUMAN] \| \| Extended synaptotagmin-1 OS=Homo sapiens GN=ESYT1 PE=1 SV=1 - [ESYT1_HUMAN] \| \| Ribonucleases P/MRP protein subunit POP1 OS=Homo sapiens GN=POP1 PE=1 SV=2 - [POP1_HUMAN] \| \| Kelch-like ECH-associated protein 1 OS=Homo sapiens GN=KEAP1 PE=1 SV=2 - [KEAP1_HUMAN] \| \| WD repeat and HMG-box DNA-binding protein 1 OS=Homo sapiens GN=WDHD1 PE=1 SV=1 - [WDHD1_HUMAN] \| \| Leucine zipper protein 1 OS=Homo sapiens GN=LUZP1 PE=1 SV=2 - [LUZP1_HUMAN] \| \| Zinc finger MYM-type protein 1 OS=Homo sapiens GN=ZMYM1 PE=1 SV=1 - [ZMYM1_HUMAN] \| \| Methionine synthase OS=Homo sapiens GN=MTR PE=1 SV=2 - [METH_HUMAN] \| \| Scaffold attachment factor B2 OS=Homo sapiens GN=SAFB2 PE=1 SV=1 - [SAFB2_HUMAN] \| \| Vinculin OS=Homo sapiens GN=VCL PE=1 SV=4 - [VINC_HUMAN] \| \| SURP and G-patch domain-containing protein 2 OS=Homo sapiens GN=SUGP2 PE=1 SV=2 - [SUGP2_HUMAN] \| \| Kinesin heavy chain isoform 5C OS=Homo sapiens GN=KIF5C PE=1 SV=1 - [KIF5C_HUMAN] \| \| Protein diaphanous homolog 3 OS=Homo sapiens GN=DIAPH3 PE=1 SV=4 - [DIAP3_HUMAN] \| \| Protein MMS22-like OS=Homo sapiens GN=MMS22L PE=1 SV=3 - [MMS22_HUMAN] \| \| Tubulin alpha-1C chain OS=Homo sapiens GN=TUBA1C PE=1 SV=1 - [TBA1C_HUMAN] \| \| Protein transport protein Sec24C OS=Homo sapiens GN=SEC24C PE=1 SV=3 - [SC24C_HUMAN] \| \| Nuclear pore complex protein Nup107 OS=Homo sapiens GN=NUP107 PE=1 SV=1 - [NU107_HUMAN] \| \| Nuclear pore complex protein Nup160 OS=Homo sapiens GN=NUP160 PE=1 SV=3 - [NU160_HUMAN] \| \| Protein SMG7 OS=Homo sapiens GN=SMG7 PE=1 SV=2 - [SMG7_HUMAN] \| \| Isoleucine--tRNA ligase, cytoplasmic OS=Homo sapiens GN=IARS PE=1 SV=2 - [SYIC_HUMAN] \| \| Putative helicase MOV-10 OS=Homo sapiens GN=MOV10 PE=1 SV=2 - [MOV10_HUMAN] \| \| ADP/ATP translocase 2 OS=Homo sapiens GN=SLC25A5 PE=1 SV=7 - [ADT2_HUMAN] \| \|  \| |
| --- | --- | --- | --- | --- | --- | --- | --- | --- | --- | --- | --- | --- | --- | --- | --- | --- | --- | --- | --- | --- | --- | --- | --- | --- | --- | --- | --- | --- | --- | --- | --- | --- | --- | --- | --- | --- | --- | --- | --- | --- | --- | --- | --- | --- | --- | --- | --- | --- | --- | --- | --- | --- | --- | --- | --- | --- | --- | --- | --- | --- | --- | --- | --- | --- | --- | --- | --- | --- | --- | --- | --- | --- | --- | --- | --- | --- | --- | --- | --- | --- | --- | --- | --- | --- | --- | --- | --- | --- | --- | --- | --- | --- | --- | --- | --- | --- | --- | --- | --- | --- | --- | --- | --- | --- | --- | --- | --- | --- | --- | --- | --- | --- | --- | --- | --- | --- | --- | --- | --- | --- | --- | --- | --- | --- | --- | --- | --- | --- | --- | --- | --- | --- | --- | --- | --- | --- | --- | --- | --- | --- | --- | --- | --- | --- | --- | --- | --- | --- | --- | --- | --- | --- | --- | --- | --- | --- | --- | --- | --- | --- | --- | --- | --- | --- | --- | --- | --- | --- | --- | --- | --- | --- | --- | --- | --- | --- | --- | --- | --- | --- | --- | --- | --- | --- | --- | --- | --- | --- | --- | --- | --- | --- | --- | --- | --- | --- | --- | --- | --- | --- | --- | --- | --- | --- | --- | --- | --- | --- | --- | --- | --- | --- | --- | --- | --- | --- | --- | --- | --- | --- | --- | --- | --- | --- | --- | --- | --- | --- | --- | --- | --- | --- | --- | --- | --- | --- | --- | --- | --- | --- | --- | --- | --- | --- | --- | --- | --- | --- | --- | --- | --- | --- | --- | --- | --- | --- | --- | --- | --- | --- | --- | --- | --- | --- | --- | --- | --- | --- | --- | --- | --- | --- | --- | --- | --- | --- | --- | --- | --- | --- | --- | --- | --- | --- | --- | --- | --- | --- | --- | --- | --- | --- | --- | --- | --- | --- | --- | --- | --- | --- | --- | --- | --- | --- | --- | --- | --- | --- | --- | --- | --- | --- | --- | --- | --- | --- | --- | --- | --- | --- | --- | --- | --- | --- | --- | --- | --- | --- | --- | --- | --- | --- | --- | --- | --- | --- | --- | --- | --- | --- | --- | --- | --- | --- | --- | --- | --- | --- | --- | --- | --- | --- | --- | --- | --- | --- | --- | --- | --- | --- | --- | --- | --- | --- | --- | --- | --- | --- | --- | --- | --- | --- | --- | --- | --- | --- | --- | --- | --- | --- | --- | --- | --- | --- | --- | --- | --- | --- | --- | --- | --- | --- | --- | --- | --- | --- | --- | --- | --- | --- | --- | --- | --- | --- | --- | --- | --- | --- | --- | --- | --- | --- | --- | --- | --- | --- | --- | --- | --- | --- | --- | --- | --- | --- | --- | --- | --- | --- | --- | --- | --- | --- | --- | --- | --- | --- | --- | --- | --- | --- | --- | --- | --- | --- | --- | --- | --- | --- | --- | --- | --- | --- | --- | --- | --- | --- | --- | --- | --- | --- | --- | --- | --- | --- | --- | --- | --- | --- | --- | --- | --- | --- | --- | --- | --- | --- | --- | --- | --- | --- | --- | --- | --- | --- | --- | --- | --- | --- | --- | --- | --- | --- | --- | --- | --- | --- | --- | --- | --- | --- | --- | --- | --- | --- | --- | --- | --- | --- | --- | --- | --- | --- | --- | --- | --- | --- | --- | --- | --- | --- | --- | --- | --- | --- | --- | --- | --- | --- | --- | --- | --- | --- | --- | --- | --- | --- | --- | --- | --- | --- | --- | --- | --- | --- | --- | --- | --- | --- | --- | --- | --- | --- | --- | --- | --- | --- | --- | --- | --- | --- | --- | --- | --- | --- | --- | --- | --- | --- | --- | --- | --- | --- | --- | --- | --- | --- | --- | --- | --- | --- | --- | --- | --- | --- | --- | --- | --- | --- | --- | --- | --- | --- | --- | --- | --- | --- | --- | --- | --- | --- | --- | --- | --- | --- | --- | --- | --- | --- | --- | --- | --- | --- | --- | --- | --- | --- | --- | --- | --- | --- | --- | --- | --- | --- | --- | --- | --- | --- | --- | --- | --- | --- | --- | --- | --- | --- | --- | --- | --- | --- | --- | --- | --- | --- | --- | --- | --- | --- | --- | --- | --- | --- | --- | --- | --- | --- | --- | --- | --- | --- | --- | --- | --- | --- | --- | --- | --- | --- | --- | --- | --- | --- | --- | --- | --- | --- | --- | --- | --- | --- | --- | --- | --- | --- | --- | --- | --- | --- | --- | --- | --- | --- | --- | --- | --- | --- | --- | --- | --- | --- | --- | --- | --- | --- | --- | --- | --- | --- | --- | --- | --- | --- | --- | --- | --- | --- | --- | --- | --- | --- | --- | --- | --- | --- | --- | --- | --- | --- | --- | --- | --- | --- | --- | --- | --- | --- | --- | --- | --- | --- | --- | --- | --- | --- | --- | --- | --- | --- | --- | --- | --- | --- | --- | --- | --- | --- | --- | --- | --- | --- | --- | --- | --- | --- | --- | --- | --- | --- | --- | --- | --- | --- | --- | --- | --- | --- | --- | --- | --- | --- | --- | --- | --- | --- | --- | --- | --- | --- | --- | --- | --- | --- | --- | --- | --- | --- | --- | --- | --- | --- | --- | --- | --- | --- | --- | --- | --- | --- | --- | --- | --- | --- | --- | --- | --- | --- | --- | --- | --- | --- | --- | --- | --- | --- | --- | --- | --- | --- | --- | --- | --- | --- | --- | --- | --- | --- | --- | --- | --- | --- | --- | --- | --- | --- | --- | --- | --- | --- | --- | --- | --- | --- | --- | --- | --- | --- | --- | --- | --- | --- | --- | --- | --- | --- | --- | --- | --- | --- | --- | --- | --- | --- | --- | --- | --- | --- | --- | --- | --- | --- | --- | --- | --- | --- | --- | --- | --- | --- | --- | --- | --- | --- | --- | --- | --- | --- | --- | --- | --- | --- | --- | --- | --- | --- | --- | --- | --- | --- | --- | --- | --- | --- | --- | --- | --- | --- | --- | --- | --- | --- | --- | --- | --- | --- | --- | --- | --- | --- | --- | --- | --- | --- | --- | --- | --- | --- | --- | --- | --- | --- | --- | --- | --- | --- | --- | --- | --- | --- | --- | --- | --- | --- | --- | --- | --- | --- | --- | --- | --- | --- | --- | --- | --- | --- | --- | --- | --- | --- | --- | --- | --- | --- | --- | --- | --- | --- | --- | --- | --- | --- | --- | --- | --- | --- | --- | --- | --- | --- | --- | --- | --- | --- | --- | --- | --- | --- | --- | --- | --- | --- | --- | --- | --- | --- | --- | --- | --- | --- | --- | --- | --- | --- | --- | --- | --- | --- | --- | --- | --- | --- | --- | --- | --- | --- | --- | --- | --- | --- | --- | --- | --- | --- | --- | --- | --- | --- | --- | --- | --- | --- | --- | --- | --- | --- | --- | --- | --- | --- | --- | --- | --- | --- | --- | --- | --- | --- | --- | --- | --- | --- | --- | --- | --- | --- | --- | --- | --- | --- | --- | --- | --- | --- | --- | --- | --- | --- | --- | --- | --- | --- | --- | --- | --- | --- | --- | --- | --- | --- | --- | --- | --- | --- | --- | --- | --- | --- | --- | --- | --- | --- | --- | --- | --- | --- | --- | --- | --- | --- | --- | --- | --- | --- | --- | --- | --- | --- | --- | --- | --- | --- | --- | --- | --- | --- | --- | --- | --- | --- | --- | --- | --- | --- | --- | --- | --- | --- | --- | --- | --- | --- | --- | --- |

**Sample C1:**

| \| Accession \| \| --- \| \| O95071 \| \| P09874 \| \| P42285 \| \| Q15029 \| \| Q9P0K7 \| \| O00159 \| \| Q99613 \| \| Q9Y2A7 \| \| P78347 \| \| P58107 \| \| Q14152 \| \| Q15149 \| \| Q9BPX3 \| \| P22314 \| \| P15924 \| \| Q15393 \| \| Q6P1N0 \| \| O43795 \| \| Q9H9Y6 \| \| P53396 \| \| Q92974 \| \| O95373 \| \| Q86XP3 \| \| Q86YS7 \| \| Q7KZF4 \| \| Q14527 \| \| Q99575 \| \| Q16531 \| \| Q9Y5B6 \| \| P33176 \| \| P51530 \| \| Q9H0A0 \| \| A6QL63 \| \| Q8TF46 \| \| Q9BXP5 \| \| Q86XI2 \| \| Q8IWX8 \| \| O00410 \| \| Q86W56 \| \| O00203 \| \| P12270 \| \| P08670 \| \| Q9Y4E8 \| \| O75122 \| \| Q9NX05 \| \| O75400 \| \| P51610 \| \| Q92900 \| \| P38935 \| \| P98175 \| \| Q05397 \| \| Q00839 \| \| P52701 \| \| Q8IUD2 \| \| Q16513 \| \| Q8N163 \| \| Q9UPP1 \| \| Q8WVB6 \| \| P55884 \| \| Q8N5C6 \| \| Q9NZC9 \| \| Q13813 \| \| O60231 \| \| P35580 \| \| O15042 \| \| Q96QC0 \| \| Q8IWC1 \| \| Q9HCE1 \| \| Q9H2U1 \| \| Q9Y2L1 \| \| Q02241 \| \| Q8N9B5 \| \| Q5H9R7 \| \| Q12768 \| \| Q8IVF7 \| \| O94972 \| \| Q96DT7 \| \| Q6PKG0 \| \| Q08211 \| \| Q9HBG6 \| \| Q7Z2W4 \| \| Q3KQU3 \| \| O00267 \| \| Q96P70 \| \| O94762 \| \| Q8WUM0 \| \| P49756 \| \| Q7L2E3 \| \| P53992 \| \| Q9BZH6 \| \| P49736 \| \| Q9C0B7 \| \| Q8NE71 \| \| O94832 \| \| O60518 \| \| O75694 \| \| Q9BSJ8 \| \| Q69YQ0 \| \| Q15020 \| \| Q96RT8 \| \| Q9NQW6 \| \| Q93100 \| \| Q9NZB2 \| \| Q9BTW9 \| \| Q8N960 \| \| O75044 \| \| O94906 \| \| Q96KR1 \| \| P43243 \| \| Q86W92 \| \| Q7Z6B7 \| \|  \| | \| Coverage \| \| --- \| \| 19.65 \| \| 18.05 \| \| 20.15 \| \| 32.30 \| \| 19.69 \| \| 20.88 \| \| 12.60 \| \| 24.20 \| \| 13.93 \| \| 19.41 \| \| 10.13 \| \| 4.78 \| \| 13.60 \| \| 14.37 \| \| 5.96 \| \| 12.41 \| \| 16.19 \| \| 11.71 \| \| 10.13 \| \| 9.81 \| \| 10.34 \| \| 6.07 \| \| 11.41 \| \| 9.90 \| \| 6.48 \| \| 5.55 \| \| 10.35 \| \| 16.05 \| \| 11.56 \| \| 8.10 \| \| 6.60 \| \| 6.63 \| \| 4.80 \| \| 7.50 \| \| 9.02 \| \| 6.91 \| \| 7.21 \| \| 8.75 \| \| 6.25 \| \| 8.68 \| \| 2.75 \| \| 11.16 \| \| 5.20 \| \| 4.33 \| \| 5.20 \| \| 5.12 \| \| 1.18 \| \| 5.31 \| \| 5.74 \| \| 6.77 \| \| 6.18 \| \| 3.27 \| \| 6.62 \| \| 2.69 \| \| 11.48 \| \| 6.50 \| \| 2.55 \| \| 7.18 \| \| 4.30 \| \| 5.23 \| \| 4.40 \| \| 1.54 \| \| 7.01 \| \| 1.52 \| \| 2.53 \| \| 6.17 \| \| 3.08 \| \| 1.99 \| \| 5.06 \| \| 2.40 \| \| 4.48 \| \| 4.96 \| \| 5.96 \| \| 3.45 \| \| 3.31 \| \| 3.42 \| \| 4.59 \| \| 3.56 \| \| 3.23 \| \| 3.71 \| \| 3.22 \| \| 2.97 \| \| 4.78 \| \| 3.46 \| \| 4.74 \| \| 3.55 \| \| 3.68 \| \| 1.59 \| \| 4.66 \| \| 3.10 \| \| 3.98 \| \| 2.56 \| \| 2.72 \| \| 5.27 \| \| 3.53 \| \| 1.51 \| \| 2.17 \| \| 1.88 \| \| 2.18 \| \| 1.86 \| \| 2.67 \| \| 3.39 \| \| 2.24 \| \| 1.93 \| \| 2.13 \| \| 1.77 \| \| 3.08 \| \| 3.35 \| \| 8.26 \| \| 3.56 \| \| 1.75 \| \|  \| | \| # PSMs \| \| --- \| \| 118 \| \| 43 \| \| 50 \| \| 49 \| \| 32 \| \| 40 \| \| 30 \| \| 39 \| \| 26 \| \| 32 \| \| 18 \| \| 32 \| \| 21 \| \| 24 \| \| 29 \| \| 24 \| \| 23 \| \| 18 \| \| 16 \| \| 16 \| \| 16 \| \| 13 \| \| 14 \| \| 17 \| \| 11 \| \| 8 \| \| 13 \| \| 20 \| \| 17 \| \| 14 \| \| 12 \| \| 10 \| \| 8 \| \| 15 \| \| 12 \| \| 10 \| \| 13 \| \| 11 \| \| 10 \| \| 11 \| \| 11 \| \| 7 \| \| 8 \| \| 8 \| \| 7 \| \| 5 \| \| 5 \| \| 9 \| \| 10 \| \| 9 \| \| 11 \| \| 4 \| \| 14 \| \| 6 \| \| 13 \| \| 9 \| \| 4 \| \| 8 \| \| 5 \| \| 9 \| \| 6 \| \| 4 \| \| 9 \| \| 4 \| \| 4 \| \| 5 \| \| 3 \| \| 5 \| \| 7 \| \| 6 \| \| 4 \| \| 4 \| \| 7 \| \| 6 \| \| 5 \| \| 3 \| \| 4 \| \| 5 \| \| 7 \| \| 6 \| \| 3 \| \| 3 \| \| 6 \| \| 5 \| \| 5 \| \| 5 \| \| 5 \| \| 3 \| \| 4 \| \| 6 \| \| 5 \| \| 5 \| \| 3 \| \| 8 \| \| 8 \| \| 3 \| \| 4 \| \| 4 \| \| 3 \| \| 3 \| \| 3 \| \| 5 \| \| 2 \| \| 2 \| \| 2 \| \| 4 \| \| 4 \| \| 3 \| \| 5 \| \| 3 \| \| 2 \| \|  \| | \| # Peptides \| \| --- \| \| 39 \| \| 12 \| \| 16 \| \| 24 \| \| 15 \| \| 16 \| \| 11 \| \| 20 \| \| 11 \| \| 13 \| \| 11 \| \| 19 \| \| 10 \| \| 10 \| \| 14 \| \| 11 \| \| 11 \| \| 11 \| \| 9 \| \| 8 \| \| 8 \| \| 5 \| \| 8 \| \| 8 \| \| 5 \| \| 4 \| \| 8 \| \| 13 \| \| 8 \| \| 7 \| \| 6 \| \| 5 \| \| 5 \| \| 7 \| \| 6 \| \| 7 \| \| 6 \| \| 7 \| \| 5 \| \| 8 \| \| 6 \| \| 4 \| \| 4 \| \| 4 \| \| 4 \| \| 3 \| \| 2 \| \| 5 \| \| 5 \| \| 5 \| \| 6 \| \| 2 \| \| 8 \| \| 3 \| \| 9 \| \| 5 \| \| 2 \| \| 4 \| \| 3 \| \| 5 \| \| 4 \| \| 3 \| \| 6 \| \| 2 \| \| 2 \| \| 4 \| \| 2 \| \| 2 \| \| 4 \| \| 2 \| \| 3 \| \| 3 \| \| 4 \| \| 3 \| \| 3 \| \| 2 \| \| 3 \| \| 3 \| \| 4 \| \| 4 \| \| 2 \| \| 2 \| \| 4 \| \| 3 \| \| 3 \| \| 3 \| \| 3 \| \| 2 \| \| 3 \| \| 3 \| \| 3 \| \| 2 \| \| 2 \| \| 5 \| \| 3 \| \| 2 \| \| 2 \| \| 2 \| \| 2 \| \| 2 \| \| 2 \| \| 3 \| \| 2 \| \| 2 \| \| 2 \| \| 2 \| \| 3 \| \| 2 \| \| 4 \| \| 3 \| \| 2 \| \|  \| | \| # AAs \| \| --- \| \| 2799 \| \| 1014 \| \| 1042 \| \| 972 \| \| 980 \| \| 1063 \| \| 913 \| \| 1128 \| \| 998 \| \| 5090 \| \| 1382 \| \| 4684 \| \| 1015 \| \| 1058 \| \| 2871 \| \| 1217 \| \| 951 \| \| 1136 \| \| 1135 \| \| 1101 \| \| 986 \| \| 1038 \| \| 938 \| \| 1000 \| \| 910 \| \| 1009 \| \| 1024 \| \| 1140 \| \| 917 \| \| 963 \| \| 1060 \| \| 1025 \| \| 1104 \| \| 1054 \| \| 876 \| \| 1143 \| \| 916 \| \| 1097 \| \| 976 \| \| 1094 \| \| 2363 \| \| 466 \| \| 981 \| \| 1294 \| \| 1096 \| \| 957 \| \| 2035 \| \| 1129 \| \| 993 \| \| 930 \| \| 1052 \| \| 825 \| \| 1360 \| \| 1116 \| \| 984 \| \| 923 \| \| 1060 \| \| 975 \| \| 814 \| \| 995 \| \| 954 \| \| 2472 \| \| 1041 \| \| 1976 \| \| 1029 \| \| 940 \| \| 876 \| \| 1003 \| \| 1008 \| \| 958 \| \| 960 \| \| 988 \| \| 873 \| \| 1159 \| \| 1028 \| \| 964 \| \| 871 \| \| 1096 \| \| 1270 \| \| 1241 \| \| 902 \| \| 841 \| \| 1087 \| \| 1041 \| \| 991 \| \| 1156 \| \| 843 \| \| 1194 \| \| 1094 \| \| 1224 \| \| 904 \| \| 1094 \| \| 845 \| \| 1006 \| \| 1105 \| \| 1391 \| \| 1104 \| \| 1117 \| \| 963 \| \| 1024 \| \| 1124 \| \| 1093 \| \| 1118 \| \| 1192 \| \| 986 \| \| 1071 \| \| 941 \| \| 1074 \| \| 847 \| \| 1011 \| \| 1085 \| \|  \| | \| MW [kDa] \| \| --- \| \| 309.2 \| \| 113.0 \| \| 117.7 \| \| 109.4 \| \| 110.0 \| \| 121.6 \| \| 105.3 \| \| 128.7 \| \| 112.3 \| \| 555.3 \| \| 166.5 \| \| 531.5 \| \| 114.3 \| \| 117.8 \| \| 331.6 \| \| 135.5 \| \| 104.0 \| \| 131.9 \| \| 128.1 \| \| 120.8 \| \| 111.5 \| \| 119.4 \| \| 102.9 \| \| 110.4 \| \| 101.9 \| \| 113.9 \| \| 114.6 \| \| 126.9 \| \| 104.7 \| \| 109.6 \| \| 120.3 \| \| 115.7 \| \| 120.8 \| \| 120.7 \| \| 100.6 \| \| 130.9 \| \| 103.6 \| \| 123.5 \| \| 111.0 \| \| 121.2 \| \| 267.1 \| \| 53.6 \| \| 112.3 \| \| 141.0 \| \| 120.5 \| \| 108.7 \| \| 208.6 \| \| 124.3 \| \| 109.1 \| \| 103.5 \| \| 119.2 \| \| 90.5 \| \| 152.7 \| \| 128.0 \| \| 112.0 \| \| 102.8 \| \| 117.8 \| \| 107.3 \| \| 92.4 \| \| 111.7 \| \| 105.9 \| \| 284.4 \| \| 119.2 \| \| 228.9 \| \| 118.2 \| \| 99.0 \| \| 98.4 \| \| 113.6 \| \| 114.7 \| \| 108.9 \| \| 110.0 \| \| 111.4 \| \| 97.6 \| \| 134.2 \| \| 117.1 \| \| 107.8 \| \| 94.8 \| \| 123.4 \| \| 140.9 \| \| 141.7 \| \| 101.4 \| \| 92.8 \| \| 120.9 \| \| 115.9 \| \| 108.8 \| \| 128.9 \| \| 100.1 \| \| 133.9 \| \| 118.2 \| \| 136.6 \| \| 101.8 \| \| 120.7 \| \| 95.9 \| \| 116.1 \| \| 124.6 \| \| 155.1 \| \| 122.8 \| \| 124.5 \| \| 109.9 \| \| 118.2 \| \| 124.1 \| \| 124.8 \| \| 121.8 \| \| 132.5 \| \| 112.6 \| \| 120.8 \| \| 106.9 \| \| 116.9 \| \| 94.6 \| \| 114.0 \| \| 124.2 \| \|  \| | \| calc. pI \| \| --- \| \| 5.85 \| \| 8.88 \| \| 6.52 \| \| 5.00 \| \| 6.21 \| \| 9.41 \| \| 5.68 \| \| 6.62 \| \| 6.39 \| \| 5.60 \| \| 6.79 \| \| 5.96 \| \| 5.59 \| \| 5.76 \| \| 6.81 \| \| 5.26 \| \| 8.09 \| \| 9.38 \| \| 7.83 \| \| 7.33 \| \| 7.27 \| \| 4.82 \| \| 7.02 \| \| 5.69 \| \| 7.17 \| \| 8.60 \| \| 9.22 \| \| 5.26 \| \| 5.68 \| \| 6.51 \| \| 7.74 \| \| 8.27 \| \| 6.83 \| \| 6.54 \| \| 5.96 \| \| 6.87 \| \| 9.04 \| \| 4.94 \| \| 6.43 \| \| 6.04 \| \| 5.02 \| \| 5.12 \| \| 5.22 \| \| 8.47 \| \| 9.03 \| \| 7.56 \| \| 7.46 \| \| 6.61 \| \| 8.97 \| \| 5.97 \| \| 6.62 \| \| 6.00 \| \| 6.90 \| \| 5.97 \| \| 6.30 \| \| 5.22 \| \| 8.72 \| \| 7.21 \| \| 5.00 \| \| 8.72 \| \| 9.06 \| \| 5.35 \| \| 6.80 \| \| 5.54 \| \| 8.47 \| \| 9.17 \| \| 9.32 \| \| 8.82 \| \| 7.68 \| \| 7.14 \| \| 8.51 \| \| 6.18 \| \| 4.60 \| \| 6.98 \| \| 6.65 \| \| 5.15 \| \| 5.16 \| \| 8.82 \| \| 6.84 \| \| 6.49 \| \| 8.40 \| \| 10.11 \| \| 5.06 \| \| 4.81 \| \| 8.56 \| \| 5.10 \| \| 6.32 \| \| 8.78 \| \| 7.06 \| \| 6.92 \| \| 5.52 \| \| 6.11 \| \| 6.80 \| \| 9.39 \| \| 5.01 \| \| 6.16 \| \| 5.83 \| \| 5.72 \| \| 5.57 \| \| 5.90 \| \| 8.07 \| \| 6.95 \| \| 8.88 \| \| 6.19 \| \| 6.25 \| \| 6.70 \| \| 8.25 \| \| 9.04 \| \| 6.25 \| \| 5.55 \| \| 6.83 \| \|  \| | \| Score \| \| --- \| \| 2027.08 \| \| 1059.12 \| \| 938.61 \| \| 916.35 \| \| 811.42 \| \| 759.28 \| \| 641.49 \| \| 616.60 \| \| 555.07 \| \| 540.38 \| \| 498.26 \| \| 472.40 \| \| 466.21 \| \| 462.31 \| \| 433.83 \| \| 429.99 \| \| 424.96 \| \| 408.11 \| \| 351.43 \| \| 345.91 \| \| 327.48 \| \| 315.28 \| \| 301.17 \| \| 300.11 \| \| 286.14 \| \| 285.20 \| \| 284.17 \| \| 264.27 \| \| 254.03 \| \| 240.05 \| \| 225.78 \| \| 220.39 \| \| 207.27 \| \| 206.68 \| \| 197.61 \| \| 196.35 \| \| 194.96 \| \| 192.02 \| \| 182.75 \| \| 179.27 \| \| 178.59 \| \| 175.00 \| \| 170.87 \| \| 170.20 \| \| 163.45 \| \| 162.10 \| \| 161.37 \| \| 160.49 \| \| 154.69 \| \| 153.90 \| \| 149.44 \| \| 142.73 \| \| 141.70 \| \| 141.39 \| \| 141.23 \| \| 139.40 \| \| 136.27 \| \| 126.24 \| \| 120.32 \| \| 116.56 \| \| 116.27 \| \| 115.97 \| \| 115.58 \| \| 114.74 \| \| 114.63 \| \| 113.42 \| \| 113.00 \| \| 112.96 \| \| 110.47 \| \| 105.22 \| \| 103.77 \| \| 100.38 \| \| 98.83 \| \| 97.94 \| \| 97.20 \| \| 94.11 \| \| 93.51 \| \| 92.24 \| \| 90.28 \| \| 85.86 \| \| 85.30 \| \| 83.58 \| \| 82.64 \| \| 77.54 \| \| 77.10 \| \| 76.85 \| \| 68.60 \| \| 68.45 \| \| 68.31 \| \| 64.71 \| \| 61.64 \| \| 59.62 \| \| 57.95 \| \| 57.35 \| \| 55.47 \| \| 54.89 \| \| 54.28 \| \| 53.47 \| \| 51.91 \| \| 51.30 \| \| 50.98 \| \| 47.83 \| \| 46.88 \| \| 43.28 \| \| 40.48 \| \| 40.42 \| \| 39.90 \| \| 31.68 \| \| 31.33 \| \| 30.55 \| \| 19.84 \| \|  \| | \| Description \| \| --- \| \| E3 ubiquitin-protein ligase UBR5 OS=Homo sapiens GN=UBR5 PE=1 SV=2 - [UBR5_HUMAN] \| \| Poly [ADP-ribose] polymerase 1 OS=Homo sapiens GN=PARP1 PE=1 SV=4 - [PARP1_HUMAN] \| \| Superkiller viralicidic activity 2-like 2 OS=Homo sapiens GN=SKIV2L2 PE=1 SV=3 - [SK2L2_HUMAN] \| \| 116 kDa U5 small nuclear ribonucleoprotein component OS=Homo sapiens GN=EFTUD2 PE=1 SV=1 - [U5S1_HUMAN] \| \| Ankycorbin OS=Homo sapiens GN=RAI14 PE=1 SV=2 - [RAI14_HUMAN] \| \| Unconventional myosin-Ic OS=Homo sapiens GN=MYO1C PE=1 SV=4 - [MYO1C_HUMAN] \| \| Eukaryotic translation initiation factor 3 subunit C OS=Homo sapiens GN=EIF3C PE=1 SV=1 - [EIF3C_HUMAN] \| \| Nck-associated protein 1 OS=Homo sapiens GN=NCKAP1 PE=1 SV=1 - [NCKP1_HUMAN] \| \| General transcription factor II-I OS=Homo sapiens GN=GTF2I PE=1 SV=2 - [GTF2I_HUMAN] \| \| Epiplakin OS=Homo sapiens GN=EPPK1 PE=1 SV=2 - [EPIPL_HUMAN] \| \| Eukaryotic translation initiation factor 3 subunit A OS=Homo sapiens GN=EIF3A PE=1 SV=1 - [EIF3A_HUMAN] \| \| Plectin OS=Homo sapiens GN=PLEC PE=1 SV=3 - [PLEC_HUMAN] \| \| Condensin complex subunit 3 OS=Homo sapiens GN=NCAPG PE=1 SV=1 - [CND3_HUMAN] \| \| Ubiquitin-like modifier-activating enzyme 1 OS=Homo sapiens GN=UBA1 PE=1 SV=3 - [UBA1_HUMAN] \| \| Desmoplakin OS=Homo sapiens GN=DSP PE=1 SV=3 - [DESP_HUMAN] \| \| Splicing factor 3B subunit 3 OS=Homo sapiens GN=SF3B3 PE=1 SV=4 - [SF3B3_HUMAN] \| \| Coiled-coil and C2 domain-containing protein 1A OS=Homo sapiens GN=CC2D1A PE=1 SV=1 - [C2D1A_HUMAN] \| \| Unconventional myosin-Ib OS=Homo sapiens GN=MYO1B PE=2 SV=3 - [MYO1B_HUMAN] \| \| DNA-directed RNA polymerase I subunit RPA2 OS=Homo sapiens GN=POLR1B PE=1 SV=2 - [RPA2_HUMAN] \| \| ATP-citrate synthase OS=Homo sapiens GN=ACLY PE=1 SV=3 - [ACLY_HUMAN] \| \| Rho guanine nucleotide exchange factor 2 OS=Homo sapiens GN=ARHGEF2 PE=1 SV=4 - [ARHG2_HUMAN] \| \| Importin-7 OS=Homo sapiens GN=IPO7 PE=1 SV=1 - [IPO7_HUMAN] \| \| ATP-dependent RNA helicase DDX42 OS=Homo sapiens GN=DDX42 PE=1 SV=1 - [DDX42_HUMAN] \| \| Uncharacterized protein KIAA0528 OS=Homo sapiens GN=KIAA0528 PE=1 SV=1 - [K0528_HUMAN] \| \| Staphylococcal nuclease domain-containing protein 1 OS=Homo sapiens GN=SND1 PE=1 SV=1 - [SND1_HUMAN] \| \| Helicase-like transcription factor OS=Homo sapiens GN=HLTF PE=1 SV=2 - [HLTF_HUMAN] \| \| Ribonucleases P/MRP protein subunit POP1 OS=Homo sapiens GN=POP1 PE=1 SV=2 - [POP1_HUMAN] \| \| DNA damage-binding protein 1 OS=Homo sapiens GN=DDB1 PE=1 SV=1 - [DDB1_HUMAN] \| \| GC-rich sequence DNA-binding factor 1 OS=Homo sapiens GN=GCFC1 PE=1 SV=2 - [GCFC1_HUMAN] \| \| Kinesin-1 heavy chain OS=Homo sapiens GN=KIF5B PE=1 SV=1 - [KINH_HUMAN] \| \| DNA2-like helicase OS=Homo sapiens GN=DNA2 PE=1 SV=3 - [DNA2L_HUMAN] \| \| N-acetyltransferase 10 OS=Homo sapiens GN=NAT10 PE=1 SV=2 - [NAT10_HUMAN] \| \| Ankyrin repeat and BTB/POZ domain-containing protein BTBD11 OS=Homo sapiens GN=BTBD11 PE=2 SV=3 - [BTBDB_HUMAN] \| \| DIS3-like exonuclease 1 OS=Homo sapiens GN=DIS3L PE=1 SV=2 - [DI3L1_HUMAN] \| \| Serrate RNA effector molecule homolog OS=Homo sapiens GN=SRRT PE=1 SV=1 - [SRRT_HUMAN] \| \| Condensin-2 complex subunit G2 OS=Homo sapiens GN=NCAPG2 PE=1 SV=1 - [CNDG2_HUMAN] \| \| Calcium homeostasis endoplasmic reticulum protein OS=Homo sapiens GN=CHERP PE=1 SV=3 - [CHERP_HUMAN] \| \| Importin-5 OS=Homo sapiens GN=IPO5 PE=1 SV=4 - [IPO5_HUMAN] \| \| Poly(ADP-ribose) glycohydrolase OS=Homo sapiens GN=PARG PE=1 SV=1 - [PARG_HUMAN] \| \| AP-3 complex subunit beta-1 OS=Homo sapiens GN=AP3B1 PE=1 SV=3 - [AP3B1_HUMAN] \| \| Nucleoprotein TPR OS=Homo sapiens GN=TPR PE=1 SV=3 - [TPR_HUMAN] \| \| Vimentin OS=Homo sapiens GN=VIM PE=1 SV=4 - [VIME_HUMAN] \| \| Ubiquitin carboxyl-terminal hydrolase 15 OS=Homo sapiens GN=USP15 PE=1 SV=3 - [UBP15_HUMAN] \| \| CLIP-associating protein 2 OS=Homo sapiens GN=CLASP2 PE=1 SV=2 - [CLAP2_HUMAN] \| \| Constitutive coactivator of PPAR-gamma-like protein 2 OS=Homo sapiens GN=FAM120C PE=2 SV=3 - [F120C_HUMAN] \| \| Pre-mRNA-processing factor 40 homolog A OS=Homo sapiens GN=PRPF40A PE=1 SV=2 - [PR40A_HUMAN] \| \| Host cell factor 1 OS=Homo sapiens GN=HCFC1 PE=1 SV=2 - [HCFC1_HUMAN] \| \| Regulator of nonsense transcripts 1 OS=Homo sapiens GN=UPF1 PE=1 SV=2 - [RENT1_HUMAN] \| \| DNA-binding protein SMUBP-2 OS=Homo sapiens GN=IGHMBP2 PE=1 SV=3 - [SMBP2_HUMAN] \| \| RNA-binding protein 10 OS=Homo sapiens GN=RBM10 PE=1 SV=3 - [RBM10_HUMAN] \| \| Focal adhesion kinase 1 OS=Homo sapiens GN=PTK2 PE=1 SV=2 - [FAK1_HUMAN] \| \| Heterogeneous nuclear ribonucleoprotein U OS=Homo sapiens GN=HNRNPU PE=1 SV=6 - [HNRPU_HUMAN] \| \| DNA mismatch repair protein Msh6 OS=Homo sapiens GN=MSH6 PE=1 SV=2 - [MSH6_HUMAN] \| \| ELKS/Rab6-interacting/CAST family member 1 OS=Homo sapiens GN=ERC1 PE=1 SV=1 - [RB6I2_HUMAN] \| \| Serine/threonine-protein kinase N2 OS=Homo sapiens GN=PKN2 PE=1 SV=1 - [PKN2_HUMAN] \| \| DBIRD complex subunit KIAA1967 OS=Homo sapiens GN=KIAA1967 PE=1 SV=2 - [K1967_HUMAN] \| \| Histone lysine demethylase PHF8 OS=Homo sapiens GN=PHF8 PE=1 SV=3 - [PHF8_HUMAN] \| \| Chromosome transmission fidelity protein 18 homolog OS=Homo sapiens GN=CHTF18 PE=1 SV=1 - [CTF18_HUMAN] \| \| Eukaryotic translation initiation factor 3 subunit B OS=Homo sapiens GN=EIF3B PE=1 SV=3 - [EIF3B_HUMAN] \| \| S1 RNA-binding domain-containing protein 1 OS=Homo sapiens GN=SRBD1 PE=1 SV=2 - [SRBD1_HUMAN] \| \| SWI/SNF-related matrix-associated actin-dependent regulator of chromatin subfamily A-like protein 1 OS=Homo sapiens GN=SMARCAL1 PE=1 SV=1 - [SMAL1_HUMAN] \| \| Spectrin alpha chain, brain OS=Homo sapiens GN=SPTAN1 PE=1 SV=3 - [SPTA2_HUMAN] \| \| Putative pre-mRNA-splicing factor ATP-dependent RNA helicase DHX16 OS=Homo sapiens GN=DHX16 PE=1 SV=2 - [DHX16_HUMAN] \| \| Myosin-10 OS=Homo sapiens GN=MYH10 PE=1 SV=3 - [MYH10_HUMAN] \| \| U2 snRNP-associated SURP motif-containing protein OS=Homo sapiens GN=U2SURP PE=1 SV=2 - [SR140_HUMAN] \| \| Serine/threonine-protein phosphatase 1 regulatory subunit 10 OS=Homo sapiens GN=PPP1R10 PE=1 SV=1 - [PP1RA_HUMAN] \| \| MAP7 domain-containing protein 3 OS=Homo sapiens GN=MAP7D3 PE=1 SV=2 - [MA7D3_HUMAN] \| \| Putative helicase MOV-10 OS=Homo sapiens GN=MOV10 PE=1 SV=2 - [MOV10_HUMAN] \| \| Probable ATP-dependent RNA helicase DHX36 OS=Homo sapiens GN=DHX36 PE=1 SV=2 - [DHX36_HUMAN] \| \| Exosome complex exonuclease RRP44 OS=Homo sapiens GN=DIS3 PE=1 SV=2 - [RRP44_HUMAN] \| \| Kinesin-like protein KIF23 OS=Homo sapiens GN=KIF23 PE=1 SV=3 - [KIF23_HUMAN] \| \| Junction-mediating and -regulatory protein OS=Homo sapiens GN=JMY PE=1 SV=2 - [JMY_HUMAN] \| \| Serine/threonine-protein phosphatase 6 regulatory subunit 3 OS=Homo sapiens GN=PPP6R3 PE=1 SV=2 - [PP6R3_HUMAN] \| \| WASH complex subunit strumpellin OS=Homo sapiens GN=KIAA0196 PE=1 SV=1 - [STRUM_HUMAN] \| \| Formin-like protein 3 OS=Homo sapiens GN=FMNL3 PE=1 SV=3 - [FMNL3_HUMAN] \| \| E3 ubiquitin-protein ligase TRIM37 OS=Homo sapiens GN=TRIM37 PE=1 SV=2 - [TRI37_HUMAN] \| \| Zinc finger and BTB domain-containing protein 10 OS=Homo sapiens GN=ZBTB10 PE=1 SV=2 - [ZBT10_HUMAN] \| \| La-related protein 1 OS=Homo sapiens GN=LARP1 PE=1 SV=2 - [LARP1_HUMAN] \| \| ATP-dependent RNA helicase A OS=Homo sapiens GN=DHX9 PE=1 SV=4 - [DHX9_HUMAN] \| \| Intraflagellar transport protein 122 homolog OS=Homo sapiens GN=IFT122 PE=1 SV=2 - [IF122_HUMAN] \| \| Zinc finger CCCH-type antiviral protein 1 OS=Homo sapiens GN=ZC3HAV1 PE=1 SV=3 - [ZCCHV_HUMAN] \| \| MAP7 domain-containing protein 1 OS=Homo sapiens GN=MAP7D1 PE=1 SV=1 - [MA7D1_HUMAN] \| \| Transcription elongation factor SPT5 OS=Homo sapiens GN=SUPT5H PE=1 SV=1 - [SPT5H_HUMAN] \| \| Importin-9 OS=Homo sapiens GN=IPO9 PE=1 SV=3 - [IPO9_HUMAN] \| \| ATP-dependent DNA helicase Q5 OS=Homo sapiens GN=RECQL5 PE=1 SV=2 - [RECQ5_HUMAN] \| \| Nuclear pore complex protein Nup133 OS=Homo sapiens GN=NUP133 PE=1 SV=2 - [NU133_HUMAN] \| \| RNA-binding protein 25 OS=Homo sapiens GN=RBM25 PE=1 SV=3 - [RBM25_HUMAN] \| \| Putative ATP-dependent RNA helicase DHX30 OS=Homo sapiens GN=DHX30 PE=1 SV=1 - [DHX30_HUMAN] \| \| Protein transport protein Sec24C OS=Homo sapiens GN=SEC24C PE=1 SV=3 - [SC24C_HUMAN] \| \| WD repeat-containing protein 11 OS=Homo sapiens GN=WDR11 PE=1 SV=1 - [WDR11_HUMAN] \| \| DNA replication licensing factor MCM2 OS=Homo sapiens GN=MCM2 PE=1 SV=4 - [MCM2_HUMAN] \| \| Transmembrane and coiled-coil domain-containing protein 7 OS=Homo sapiens GN=TMCO7 PE=2 SV=2 - [TMCO7_HUMAN] \| \| ATP-binding cassette sub-family F member 1 OS=Homo sapiens GN=ABCF1 PE=1 SV=2 - [ABCF1_HUMAN] \| \| Unconventional myosin-Id OS=Homo sapiens GN=MYO1D PE=1 SV=2 - [MYO1D_HUMAN] \| \| Ran-binding protein 6 OS=Homo sapiens GN=RANBP6 PE=1 SV=2 - [RNBP6_HUMAN] \| \| Nuclear pore complex protein Nup155 OS=Homo sapiens GN=NUP155 PE=1 SV=1 - [NU155_HUMAN] \| \| Extended synaptotagmin-1 OS=Homo sapiens GN=ESYT1 PE=1 SV=1 - [ESYT1_HUMAN] \| \| Cytospin-A OS=Homo sapiens GN=SPECC1L PE=1 SV=2 - [CYTSA_HUMAN] \| \| Squamous cell carcinoma antigen recognized by T-cells 3 OS=Homo sapiens GN=SART3 PE=1 SV=1 - [SART3_HUMAN] \| \| Gamma-tubulin complex component 5 OS=Homo sapiens GN=TUBGCP5 PE=1 SV=1 - [GCP5_HUMAN] \| \| Actin-binding protein anillin OS=Homo sapiens GN=ANLN PE=1 SV=2 - [ANLN_HUMAN] \| \| Phosphorylase b kinase regulatory subunit beta OS=Homo sapiens GN=PHKB PE=1 SV=3 - [KPBB_HUMAN] \| \| Constitutive coactivator of PPAR-gamma-like protein 1 OS=Homo sapiens GN=FAM120A PE=1 SV=2 - [F120A_HUMAN] \| \| Tubulin-specific chaperone D OS=Homo sapiens GN=TBCD PE=1 SV=2 - [TBCD_HUMAN] \| \| Centrosomal protein of 120 kDa OS=Homo sapiens GN=CEP120 PE=2 SV=2 - [CE120_HUMAN] \| \| SLIT-ROBO Rho GTPase-activating protein 2 OS=Homo sapiens GN=SRGAP2 PE=1 SV=2 - [FNBP2_HUMAN] \| \| Pre-mRNA-processing factor 6 OS=Homo sapiens GN=PRPF6 PE=1 SV=1 - [PRP6_HUMAN] \| \| Zinc finger RNA-binding protein OS=Homo sapiens GN=ZFR PE=1 SV=2 - [ZFR_HUMAN] \| \| Matrin-3 OS=Homo sapiens GN=MATR3 PE=1 SV=2 - [MATR3_HUMAN] \| \| Liprin-beta-1 OS=Homo sapiens GN=PPFIBP1 PE=1 SV=2 - [LIPB1_HUMAN] \| \| SLIT-ROBO Rho GTPase-activating protein 1 OS=Homo sapiens GN=SRGAP1 PE=1 SV=1 - [SRGP1_HUMAN] \| \|  \| |
| --- | --- | --- | --- | --- | --- | --- | --- | --- | --- | --- | --- | --- | --- | --- | --- | --- | --- | --- | --- | --- | --- | --- | --- | --- | --- | --- | --- | --- | --- | --- | --- | --- | --- | --- | --- | --- | --- | --- | --- | --- | --- | --- | --- | --- | --- | --- | --- | --- | --- | --- | --- | --- | --- | --- | --- | --- | --- | --- | --- | --- | --- | --- | --- | --- | --- | --- | --- | --- | --- | --- | --- | --- | --- | --- | --- | --- | --- | --- | --- | --- | --- | --- | --- | --- | --- | --- | --- | --- | --- | --- | --- | --- | --- | --- | --- | --- | --- | --- | --- | --- | --- | --- | --- | --- | --- | --- | --- | --- | --- | --- | --- | --- | --- | --- | --- | --- | --- | --- | --- | --- | --- | --- | --- | --- | --- | --- | --- | --- | --- | --- | --- | --- | --- | --- | --- | --- | --- | --- | --- | --- | --- | --- | --- | --- | --- | --- | --- | --- | --- | --- | --- | --- | --- | --- | --- | --- | --- | --- | --- | --- | --- | --- | --- | --- | --- | --- | --- | --- | --- | --- | --- | --- | --- | --- | --- | --- | --- | --- | --- | --- | --- | --- | --- | --- | --- | --- | --- | --- | --- | --- | --- | --- | --- | --- | --- | --- | --- | --- | --- | --- | --- | --- | --- | --- | --- | --- | --- | --- | --- | --- | --- | --- | --- | --- | --- | --- | --- | --- | --- | --- | --- | --- | --- | --- | --- | --- | --- | --- | --- | --- | --- | --- | --- | --- | --- | --- | --- | --- | --- | --- | --- | --- | --- | --- | --- | --- | --- | --- | --- | --- | --- | --- | --- | --- | --- | --- | --- | --- | --- | --- | --- | --- | --- | --- | --- | --- | --- | --- | --- | --- | --- | --- | --- | --- | --- | --- | --- | --- | --- | --- | --- | --- | --- | --- | --- | --- | --- | --- | --- | --- | --- | --- | --- | --- | --- | --- | --- | --- | --- | --- | --- | --- | --- | --- | --- | --- | --- | --- | --- | --- | --- | --- | --- | --- | --- | --- | --- | --- | --- | --- | --- | --- | --- | --- | --- | --- | --- | --- | --- | --- | --- | --- | --- | --- | --- | --- | --- | --- | --- | --- | --- | --- | --- | --- | --- | --- | --- | --- | --- | --- | --- | --- | --- | --- | --- | --- | --- | --- | --- | --- | --- | --- | --- | --- | --- | --- | --- | --- | --- | --- | --- | --- | --- | --- | --- | --- | --- | --- | --- | --- | --- | --- | --- | --- | --- | --- | --- | --- | --- | --- | --- | --- | --- | --- | --- | --- | --- | --- | --- | --- | --- | --- | --- | --- | --- | --- | --- | --- | --- | --- | --- | --- | --- | --- | --- | --- | --- | --- | --- | --- | --- | --- | --- | --- | --- | --- | --- | --- | --- | --- | --- | --- | --- | --- | --- | --- | --- | --- | --- | --- | --- | --- | --- | --- | --- | --- | --- | --- | --- | --- | --- | --- | --- | --- | --- | --- | --- | --- | --- | --- | --- | --- | --- | --- | --- | --- | --- | --- | --- | --- | --- | --- | --- | --- | --- | --- | --- | --- | --- | --- | --- | --- | --- | --- | --- | --- | --- | --- | --- | --- | --- | --- | --- | --- | --- | --- | --- | --- | --- | --- | --- | --- | --- | --- | --- | --- | --- | --- | --- | --- | --- | --- | --- | --- | --- | --- | --- | --- | --- | --- | --- | --- | --- | --- | --- | --- | --- | --- | --- | --- | --- | --- | --- | --- | --- | --- | --- | --- | --- | --- | --- | --- | --- | --- | --- | --- | --- | --- | --- | --- | --- | --- | --- | --- | --- | --- | --- | --- | --- | --- | --- | --- | --- | --- | --- | --- | --- | --- | --- | --- | --- | --- | --- | --- | --- | --- | --- | --- | --- | --- | --- | --- | --- | --- | --- | --- | --- | --- | --- | --- | --- | --- | --- | --- | --- | --- | --- | --- | --- | --- | --- | --- | --- | --- | --- | --- | --- | --- | --- | --- | --- | --- | --- | --- | --- | --- | --- | --- | --- | --- | --- | --- | --- | --- | --- | --- | --- | --- | --- | --- | --- | --- | --- | --- | --- | --- | --- | --- | --- | --- | --- | --- | --- | --- | --- | --- | --- | --- | --- | --- | --- | --- | --- | --- | --- | --- | --- | --- | --- | --- | --- | --- | --- | --- | --- | --- | --- | --- | --- | --- | --- | --- | --- | --- | --- | --- | --- | --- | --- | --- | --- | --- | --- | --- | --- | --- | --- | --- | --- | --- | --- | --- | --- | --- | --- | --- | --- | --- | --- | --- | --- | --- | --- | --- | --- | --- | --- | --- | --- | --- | --- | --- | --- | --- | --- | --- | --- | --- | --- | --- | --- | --- | --- | --- | --- | --- | --- | --- | --- | --- | --- | --- | --- | --- | --- | --- | --- | --- | --- | --- | --- | --- | --- | --- | --- | --- | --- | --- | --- | --- | --- | --- | --- | --- | --- | --- | --- | --- | --- | --- | --- | --- | --- | --- | --- | --- | --- | --- | --- | --- | --- | --- | --- | --- | --- | --- | --- | --- | --- | --- | --- | --- | --- | --- | --- | --- | --- | --- | --- | --- | --- | --- | --- | --- | --- | --- | --- | --- | --- | --- | --- | --- | --- | --- | --- | --- | --- | --- | --- | --- | --- | --- | --- | --- | --- | --- | --- | --- | --- | --- | --- | --- | --- | --- | --- | --- | --- | --- | --- | --- | --- | --- | --- | --- | --- | --- | --- | --- | --- | --- | --- | --- | --- | --- | --- | --- | --- | --- | --- | --- | --- | --- | --- | --- | --- | --- | --- | --- | --- | --- | --- | --- | --- | --- | --- | --- | --- | --- | --- | --- | --- | --- | --- | --- | --- | --- | --- | --- | --- | --- | --- | --- | --- | --- | --- | --- | --- | --- | --- | --- | --- | --- | --- | --- | --- | --- | --- | --- | --- | --- | --- | --- | --- | --- | --- | --- | --- | --- | --- | --- | --- | --- | --- | --- | --- | --- | --- | --- | --- | --- | --- | --- | --- | --- | --- | --- | --- | --- | --- | --- | --- | --- | --- | --- | --- | --- | --- | --- | --- | --- | --- | --- | --- | --- | --- | --- | --- | --- | --- | --- | --- | --- | --- | --- | --- | --- | --- | --- | --- | --- | --- | --- | --- | --- | --- | --- | --- | --- | --- | --- | --- | --- | --- | --- | --- | --- | --- | --- | --- | --- | --- | --- | --- | --- | --- | --- | --- | --- | --- | --- | --- | --- | --- | --- | --- | --- | --- | --- | --- | --- | --- | --- | --- | --- | --- | --- | --- | --- | --- | --- | --- | --- | --- | --- | --- | --- | --- | --- | --- | --- | --- | --- | --- | --- | --- |

**Sample C2:**

| \| Accession \| \| --- \| \| O95071 \| \| Q9BZH6 \| \| O43795 \| \| Q7L2E3 \| \| P28340 \| \| Q08211 \| \| P58107 \| \| Q92900 \| \| Q15149 \| \| Q86VP6 \| \| P30876 \| \| Q14152 \| \| O14974 \| \| Q96KR1 \| \| Q7L014 \| \| P78347 \| \| Q9NZB2 \| \| Q9P2J5 \| \| O00411 \| \| P15924 \| \| Q6WCQ1 \| \| O60264 \| \| Q7L576 \| \| Q8WUM0 \| \| P0CG48 \| \| Q96SB8 \| \| O60231 \| \| Q8IUD2 \| \| Q13523 \| \| Q15393 \| \| Q96F07 \| \| Q93009 \| \| P42285 \| \| Q9UPP1 \| \| Q08379 \| \| P09874 \| \| P53621 \| \| O15042 \| \| P12270 \| \| P52701 \| \| Q8N9B5 \| \| P28370 \| \| P29597 \| \| P20585 \| \| Q9H6S0 \| \| Q86W56 \| \| Q8TEW0 \| \| O75044 \| \| Q14145 \| \| Q9UPN4 \| \| Q7Z6B7 \| \| O15083 \| \| Q9UHP3 \| \| Q8N3U4 \| \| Q15424 \| \| Q13435 \| \| Q9Y6X9 \| \| Q9NQW6 \| \| Q8WVS4 \| \| O75533 \| \| Q8IVF7 \| \| O75150 \| \| Q8IY18 \| \| O75122 \| \| Q7Z460 \| \| Q69YQ0 \| \| Q9P0K7 \| \| Q10570 \| \| P51610 \| \| Q8IXT5 \| \| Q14008 \| \| Q86XI2 \| \| Q8IX01 \| \| Q5T8P6 \| \| Q504Q3 \| \| Q8WWM7 \| \| Q96Q05 \| \| P49792 \| \| Q96N67 \| \| Q3KQU3 \| \| Q9NW08 \| \| P23458 \| \| Q15020 \| \| Q6P1X5 \| \| Q6PKG0 \| \| Q2M389 \| \| Q9HAV4 \| \| Q14157 \| \| Q14562 \| \| O60566 \| \| Q99569 \| \| Q9BSJ8 \| \| O00203 \| \| P52732 \| \| Q14147 \| \| O95163 \| \| Q86T82 \| \| O75694 \| \| A6QL63 \| \| Q86YS7 \| \| Q5M775 \| \| Q9UM54 \| \| Q9C0H5 \| \| O75717 \| \| Q9H7Z3 \| \| Q99613 \| \| P53992 \| \| Q6ZRV2 \| \| P35580 \| \| Q9H0E3 \| \| O75400 \| \| Q6P1N0 \| \| Q66K74 \| \| O00267 \| \| Q01082 \| \| Q8TF46 \| \| P69849 \| \| P18206 \| \| Q86V48 \| \| P78527 \| \|  \| | \| Coverage \| \| --- \| \| 20.72 \| \| 18.22 \| \| 12.76 \| \| 18.17 \| \| 18.88 \| \| 14.96 \| \| 16.82 \| \| 15.32 \| \| 4.04 \| \| 12.20 \| \| 15.67 \| \| 10.13 \| \| 10.97 \| \| 11.64 \| \| 16.10 \| \| 9.12 \| \| 15.03 \| \| 7.23 \| \| 9.84 \| \| 5.26 \| \| 10.93 \| \| 7.22 \| \| 10.53 \| \| 14.53 \| \| 32.85 \| \| 9.90 \| \| 11.24 \| \| 8.96 \| \| 4.97 \| \| 9.45 \| \| 12.75 \| \| 15.06 \| \| 9.88 \| \| 4.34 \| \| 3.39 \| \| 4.04 \| \| 5.72 \| \| 5.93 \| \| 3.81 \| \| 6.10 \| \| 8.20 \| \| 3.32 \| \| 3.37 \| \| 3.34 \| \| 3.85 \| \| 4.20 \| \| 2.51 \| \| 4.39 \| \| 8.33 \| \| 3.23 \| \| 3.69 \| \| 4.08 \| \| 3.70 \| \| 3.98 \| \| 4.04 \| \| 6.26 \| \| 3.39 \| \| 2.67 \| \| 3.85 \| \| 3.53 \| \| 2.43 \| \| 5.00 \| \| 4.36 \| \| 3.01 \| \| 2.47 \| \| 2.78 \| \| 4.39 \| \| 2.15 \| \| 1.67 \| \| 3.30 \| \| 2.26 \| \| 2.19 \| \| 3.14 \| \| 5.56 \| \| 2.16 \| \| 2.42 \| \| 5.14 \| \| 0.81 \| \| 2.99 \| \| 2.97 \| \| 2.12 \| \| 1.99 \| \| 5.09 \| \| 1.67 \| \| 3.19 \| \| 4.26 \| \| 1.66 \| \| 3.04 \| \| 1.72 \| \| 5.14 \| \| 4.95 \| \| 3.62 \| \| 5.12 \| \| 3.60 \| \| 3.67 \| \| 1.50 \| \| 4.90 \| \| 2.80 \| \| 1.72 \| \| 2.20 \| \| 3.75 \| \| 2.01 \| \| 2.40 \| \| 1.77 \| \| 2.84 \| \| 2.74 \| \| 3.84 \| \| 3.90 \| \| 3.14 \| \| 2.00 \| \| 2.51 \| \| 4.21 \| \| 2.36 \| \| 3.04 \| \| 0.80 \| \| 1.80 \| \| 3.19 \| \| 1.76 \| \| 2.42 \| \| 0.68 \| \|  \| | \| # PSMs \| \| --- \| \| 101 \| \| 43 \| \| 38 \| \| 37 \| \| 36 \| \| 30 \| \| 35 \| \| 25 \| \| 30 \| \| 20 \| \| 24 \| \| 21 \| \| 18 \| \| 23 \| \| 27 \| \| 18 \| \| 20 \| \| 13 \| \| 15 \| \| 20 \| \| 16 \| \| 16 \| \| 19 \| \| 20 \| \| 13 \| \| 19 \| \| 15 \| \| 16 \| \| 7 \| \| 13 \| \| 20 \| \| 23 \| \| 18 \| \| 8 \| \| 7 \| \| 6 \| \| 11 \| \| 11 \| \| 13 \| \| 8 \| \| 10 \| \| 9 \| \| 7 \| \| 6 \| \| 10 \| \| 6 \| \| 6 \| \| 7 \| \| 10 \| \| 5 \| \| 7 \| \| 9 \| \| 10 \| \| 8 \| \| 5 \| \| 6 \| \| 5 \| \| 4 \| \| 6 \| \| 9 \| \| 4 \| \| 7 \| \| 7 \| \| 6 \| \| 6 \| \| 7 \| \| 7 \| \| 5 \| \| 6 \| \| 5 \| \| 6 \| \| 4 \| \| 6 \| \| 6 \| \| 5 \| \| 5 \| \| 6 \| \| 3 \| \| 8 \| \| 3 \| \| 4 \| \| 3 \| \| 5 \| \| 3 \| \| 6 \| \| 6 \| \| 4 \| \| 4 \| \| 5 \| \| 6 \| \| 9 \| \| 5 \| \| 5 \| \| 4 \| \| 5 \| \| 4 \| \| 6 \| \| 5 \| \| 3 \| \| 4 \| \| 3 \| \| 3 \| \| 3 \| \| 3 \| \| 5 \| \| 3 \| \| 4 \| \| 3 \| \| 8 \| \| 2 \| \| 2 \| \| 4 \| \| 4 \| \| 3 \| \| 2 \| \| 2 \| \| 3 \| \| 3 \| \| 2 \| \| 2 \| \|  \| | \| # Peptides \| \| --- \| \| 40 \| \| 16 \| \| 14 \| \| 18 \| \| 16 \| \| 15 \| \| 16 \| \| 14 \| \| 16 \| \| 12 \| \| 14 \| \| 11 \| \| 9 \| \| 9 \| \| 14 \| \| 8 \| \| 11 \| \| 6 \| \| 9 \| \| 13 \| \| 8 \| \| 8 \| \| 11 \| \| 12 \| \| 2 \| \| 8 \| \| 9 \| \| 9 \| \| 4 \| \| 8 \| \| 14 \| \| 12 \| \| 9 \| \| 4 \| \| 3 \| \| 3 \| \| 7 \| \| 5 \| \| 7 \| \| 7 \| \| 5 \| \| 4 \| \| 3 \| \| 3 \| \| 5 \| \| 3 \| \| 3 \| \| 4 \| \| 5 \| \| 2 \| \| 4 \| \| 4 \| \| 4 \| \| 4 \| \| 3 \| \| 4 \| \| 3 \| \| 2 \| \| 3 \| \| 4 \| \| 2 \| \| 4 \| \| 4 \| \| 3 \| \| 3 \| \| 3 \| \| 4 \| \| 3 \| \| 3 \| \| 3 \| \| 4 \| \| 2 \| \| 3 \| \| 4 \| \| 3 \| \| 2 \| \| 5 \| \| 2 \| \| 5 \| \| 2 \| \| 2 \| \| 2 \| \| 4 \| \| 2 \| \| 3 \| \| 4 \| \| 2 \| \| 2 \| \| 2 \| \| 4 \| \| 5 \| \| 3 \| \| 5 \| \| 3 \| \| 3 \| \| 2 \| \| 4 \| \| 3 \| \| 2 \| \| 2 \| \| 3 \| \| 2 \| \| 2 \| \| 2 \| \| 3 \| \| 2 \| \| 3 \| \| 3 \| \| 5 \| \| 2 \| \| 2 \| \| 3 \| \| 2 \| \| 3 \| \| 2 \| \| 2 \| \| 3 \| \| 2 \| \| 2 \| \| 2 \| \|  \| | \| # AAs \| \| --- \| \| 2799 \| \| 1224 \| \| 1136 \| \| 1194 \| \| 1107 \| \| 1270 \| \| 5090 \| \| 1129 \| \| 4684 \| \| 1230 \| \| 1174 \| \| 1382 \| \| 1030 \| \| 1074 \| \| 1031 \| \| 998 \| \| 1118 \| \| 1176 \| \| 1230 \| \| 2871 \| \| 1025 \| \| 1052 \| \| 1253 \| \| 1156 \| \| 685 \| \| 1091 \| \| 1041 \| \| 1116 \| \| 1007 \| \| 1217 \| \| 1278 \| \| 1102 \| \| 1042 \| \| 1060 \| \| 1002 \| \| 1014 \| \| 1224 \| \| 1029 \| \| 2363 \| \| 1360 \| \| 988 \| \| 1054 \| \| 1187 \| \| 1137 \| \| 1430 \| \| 976 \| \| 1356 \| \| 1071 \| \| 624 \| \| 1083 \| \| 1085 \| \| 957 \| \| 1055 \| \| 1231 \| \| 915 \| \| 895 \| \| 1032 \| \| 1124 \| \| 1066 \| \| 1304 \| \| 1028 \| \| 1001 \| \| 1101 \| \| 1294 \| \| 1538 \| \| 1117 \| \| 980 \| \| 1443 \| \| 2035 \| \| 1001 \| \| 2032 \| \| 1143 \| \| 1082 \| \| 1007 \| \| 1202 \| \| 1075 \| \| 1148 \| \| 3224 \| \| 2140 \| \| 841 \| \| 1133 \| \| 1154 \| \| 963 \| \| 1199 \| \| 1096 \| \| 1173 \| \| 1204 \| \| 1087 \| \| 1220 \| \| 1050 \| \| 1192 \| \| 1104 \| \| 1094 \| \| 1056 \| \| 1143 \| \| 1332 \| \| 979 \| \| 1391 \| \| 1104 \| \| 1000 \| \| 1068 \| \| 1294 \| \| 1083 \| \| 1129 \| \| 1164 \| \| 913 \| \| 1094 \| \| 1179 \| \| 1976 \| \| 1048 \| \| 957 \| \| 951 \| \| 1059 \| \| 1087 \| \| 2364 \| \| 1054 \| \| 1222 \| \| 1134 \| \| 1076 \| \| 4128 \| \|  \| | \| MW [kDa] \| \| --- \| \| 309.2 \| \| 136.6 \| \| 131.9 \| \| 133.9 \| \| 123.6 \| \| 140.9 \| \| 555.3 \| \| 124.3 \| \| 531.5 \| \| 136.3 \| \| 133.8 \| \| 166.5 \| \| 115.2 \| \| 116.9 \| \| 117.3 \| \| 112.3 \| \| 121.8 \| \| 134.4 \| \| 138.5 \| \| 331.6 \| \| 116.5 \| \| 121.8 \| \| 145.1 \| \| 128.9 \| \| 77.0 \| \| 126.2 \| \| 119.2 \| \| 128.0 \| \| 116.9 \| \| 135.5 \| \| 148.3 \| \| 128.2 \| \| 117.7 \| \| 117.8 \| \| 113.0 \| \| 113.0 \| \| 138.3 \| \| 118.2 \| \| 267.1 \| \| 152.7 \| \| 111.4 \| \| 122.5 \| \| 133.6 \| \| 127.3 \| \| 160.1 \| \| 111.0 \| \| 151.3 \| \| 120.8 \| \| 69.6 \| \| 122.1 \| \| 124.2 \| \| 110.5 \| \| 122.1 \| \| 141.2 \| \| 102.6 \| \| 100.2 \| \| 117.7 \| \| 124.1 \| \| 122.5 \| \| 145.7 \| \| 117.1 \| \| 113.6 \| \| 128.7 \| \| 141.0 \| \| 169.3 \| \| 124.5 \| \| 110.0 \| \| 160.8 \| \| 208.6 \| \| 118.0 \| \| 225.4 \| \| 130.9 \| \| 120.1 \| \| 113.5 \| \| 135.3 \| \| 113.3 \| \| 128.4 \| \| 358.0 \| \| 242.4 \| \| 92.8 \| \| 127.7 \| \| 133.2 \| \| 109.9 \| \| 136.9 \| \| 123.4 \| \| 136.3 \| \| 136.2 \| \| 114.5 \| \| 139.2 \| \| 119.5 \| \| 131.8 \| \| 122.8 \| \| 121.2 \| \| 119.1 \| \| 128.0 \| \| 150.2 \| \| 110.1 \| \| 155.1 \| \| 120.8 \| \| 110.4 \| \| 118.5 \| \| 149.6 \| \| 121.2 \| \| 125.9 \| \| 132.6 \| \| 105.3 \| \| 118.2 \| \| 127.0 \| \| 228.9 \| \| 110.3 \| \| 108.7 \| \| 104.0 \| \| 112.1 \| \| 120.9 \| \| 274.4 \| \| 120.7 \| \| 134.0 \| \| 123.7 \| \| 120.2 \| \| 468.8 \| \|  \| | \| calc. pI \| \| --- \| \| 5.85 \| \| 6.92 \| \| 9.38 \| \| 8.78 \| \| 7.03 \| \| 6.84 \| \| 5.60 \| \| 6.61 \| \| 5.96 \| \| 5.78 \| \| 6.87 \| \| 6.79 \| \| 5.40 \| \| 9.04 \| \| 9.29 \| \| 6.39 \| \| 8.88 \| \| 7.30 \| \| 8.98 \| \| 6.81 \| \| 6.21 \| \| 8.09 \| \| 6.90 \| \| 5.10 \| \| 7.66 \| \| 6.99 \| \| 6.80 \| \| 5.97 \| \| 10.26 \| \| 5.26 \| \| 7.31 \| \| 5.55 \| \| 6.52 \| \| 8.72 \| \| 5.02 \| \| 8.88 \| \| 7.66 \| \| 8.47 \| \| 5.02 \| \| 6.90 \| \| 6.18 \| \| 8.09 \| \| 7.15 \| \| 8.02 \| \| 8.40 \| \| 6.43 \| \| 7.68 \| \| 6.70 \| \| 6.44 \| \| 8.69 \| \| 6.83 \| \| 6.99 \| \| 5.34 \| \| 5.43 \| \| 5.47 \| \| 5.67 \| \| 8.38 \| \| 8.07 \| \| 7.31 \| \| 7.09 \| \| 6.65 \| \| 6.23 \| \| 8.38 \| \| 8.47 \| \| 9.03 \| \| 5.72 \| \| 6.21 \| \| 6.40 \| \| 7.46 \| \| 6.81 \| \| 7.80 \| \| 6.87 \| \| 7.28 \| \| 9.16 \| \| 5.99 \| \| 8.59 \| \| 6.62 \| \| 6.20 \| \| 6.80 \| \| 10.11 \| \| 8.50 \| \| 7.55 \| \| 5.57 \| \| 8.19 \| \| 8.82 \| \| 7.44 \| \| 5.80 \| \| 7.11 \| \| 8.32 \| \| 5.27 \| \| 8.94 \| \| 5.83 \| \| 6.04 \| \| 5.64 \| \| 7.56 \| \| 5.94 \| \| 6.20 \| \| 6.16 \| \| 6.83 \| \| 5.69 \| \| 6.70 \| \| 8.53 \| \| 7.50 \| \| 5.62 \| \| 7.68 \| \| 5.68 \| \| 7.06 \| \| 6.98 \| \| 5.54 \| \| 9.83 \| \| 7.56 \| \| 8.09 \| \| 7.30 \| \| 5.06 \| \| 5.57 \| \| 6.54 \| \| 5.67 \| \| 5.66 \| \| 8.50 \| \| 7.12 \| \|  \| | \| Score \| \| --- \| \| 1698.48 \| \| 947.28 \| \| 686.71 \| \| 653.06 \| \| 652.65 \| \| 562.07 \| \| 539.56 \| \| 502.59 \| \| 478.68 \| \| 434.79 \| \| 409.98 \| \| 406.75 \| \| 396.09 \| \| 392.08 \| \| 378.22 \| \| 357.77 \| \| 356.83 \| \| 339.05 \| \| 328.54 \| \| 319.13 \| \| 302.19 \| \| 285.51 \| \| 282.59 \| \| 281.90 \| \| 277.10 \| \| 273.74 \| \| 268.22 \| \| 254.16 \| \| 253.02 \| \| 252.46 \| \| 242.35 \| \| 239.05 \| \| 231.48 \| \| 215.00 \| \| 208.76 \| \| 186.85 \| \| 182.94 \| \| 178.42 \| \| 164.20 \| \| 164.13 \| \| 163.81 \| \| 162.56 \| \| 158.86 \| \| 155.84 \| \| 154.56 \| \| 152.21 \| \| 150.68 \| \| 149.57 \| \| 148.36 \| \| 147.18 \| \| 142.27 \| \| 129.63 \| \| 129.18 \| \| 129.12 \| \| 122.23 \| \| 121.77 \| \| 120.16 \| \| 118.99 \| \| 117.90 \| \| 117.02 \| \| 115.99 \| \| 115.04 \| \| 109.54 \| \| 108.96 \| \| 108.80 \| \| 106.80 \| \| 106.69 \| \| 106.33 \| \| 99.76 \| \| 98.06 \| \| 97.27 \| \| 96.02 \| \| 95.30 \| \| 92.90 \| \| 92.07 \| \| 90.13 \| \| 86.81 \| \| 86.06 \| \| 84.97 \| \| 84.46 \| \| 83.14 \| \| 82.27 \| \| 80.58 \| \| 79.57 \| \| 78.28 \| \| 76.01 \| \| 75.97 \| \| 74.86 \| \| 72.72 \| \| 72.27 \| \| 70.36 \| \| 68.70 \| \| 66.86 \| \| 66.73 \| \| 66.58 \| \| 65.59 \| \| 65.02 \| \| 63.74 \| \| 63.02 \| \| 62.60 \| \| 55.93 \| \| 54.54 \| \| 53.34 \| \| 53.32 \| \| 50.49 \| \| 48.74 \| \| 47.83 \| \| 44.61 \| \| 43.31 \| \| 41.52 \| \| 41.36 \| \| 39.44 \| \| 39.38 \| \| 34.22 \| \| 32.52 \| \| 31.97 \| \| 27.25 \| \| 22.60 \| \| 21.40 \| \| 20.60 \| \|  \| | \| Description \| \| --- \| \| E3 ubiquitin-protein ligase UBR5 OS=Homo sapiens GN=UBR5 PE=1 SV=2 - [UBR5_HUMAN] \| \| WD repeat-containing protein 11 OS=Homo sapiens GN=WDR11 PE=1 SV=1 - [WDR11_HUMAN] \| \| Unconventional myosin-Ib OS=Homo sapiens GN=MYO1B PE=2 SV=3 - [MYO1B_HUMAN] \| \| Putative ATP-dependent RNA helicase DHX30 OS=Homo sapiens GN=DHX30 PE=1 SV=1 - [DHX30_HUMAN] \| \| DNA polymerase delta catalytic subunit OS=Homo sapiens GN=POLD1 PE=1 SV=2 - [DPOD1_HUMAN] \| \| ATP-dependent RNA helicase A OS=Homo sapiens GN=DHX9 PE=1 SV=4 - [DHX9_HUMAN] \| \| Epiplakin OS=Homo sapiens GN=EPPK1 PE=1 SV=2 - [EPIPL_HUMAN] \| \| Regulator of nonsense transcripts 1 OS=Homo sapiens GN=UPF1 PE=1 SV=2 - [RENT1_HUMAN] \| \| Plectin OS=Homo sapiens GN=PLEC PE=1 SV=3 - [PLEC_HUMAN] \| \| Cullin-associated NEDD8-dissociated protein 1 OS=Homo sapiens GN=CAND1 PE=1 SV=2 - [CAND1_HUMAN] \| \| DNA-directed RNA polymerase II subunit RPB2 OS=Homo sapiens GN=POLR2B PE=1 SV=1 - [RPB2_HUMAN] \| \| Eukaryotic translation initiation factor 3 subunit A OS=Homo sapiens GN=EIF3A PE=1 SV=1 - [EIF3A_HUMAN] \| \| Protein phosphatase 1 regulatory subunit 12A OS=Homo sapiens GN=PPP1R12A PE=1 SV=1 - [MYPT1_HUMAN] \| \| Zinc finger RNA-binding protein OS=Homo sapiens GN=ZFR PE=1 SV=2 - [ZFR_HUMAN] \| \| Probable ATP-dependent RNA helicase DDX46 OS=Homo sapiens GN=DDX46 PE=1 SV=2 - [DDX46_HUMAN] \| \| General transcription factor II-I OS=Homo sapiens GN=GTF2I PE=1 SV=2 - [GTF2I_HUMAN] \| \| Constitutive coactivator of PPAR-gamma-like protein 1 OS=Homo sapiens GN=FAM120A PE=1 SV=2 - [F120A_HUMAN] \| \| Leucine--tRNA ligase, cytoplasmic OS=Homo sapiens GN=LARS PE=1 SV=2 - [SYLC_HUMAN] \| \| DNA-directed RNA polymerase, mitochondrial OS=Homo sapiens GN=POLRMT PE=1 SV=2 - [RPOM_HUMAN] \| \| Desmoplakin OS=Homo sapiens GN=DSP PE=1 SV=3 - [DESP_HUMAN] \| \| Myosin phosphatase Rho-interacting protein OS=Homo sapiens GN=MPRIP PE=1 SV=3 - [MPRIP_HUMAN] \| \| SWI/SNF-related matrix-associated actin-dependent regulator of chromatin subfamily A member 5 OS=Homo sapiens GN=SMARCA5 PE=1 SV=1 - [SMCA5_HUMAN] \| \| Cytoplasmic FMR1-interacting protein 1 OS=Homo sapiens GN=CYFIP1 PE=1 SV=1 - [CYFP1_HUMAN] \| \| Nuclear pore complex protein Nup133 OS=Homo sapiens GN=NUP133 PE=1 SV=2 - [NU133_HUMAN] \| \| Polyubiquitin-C OS=Homo sapiens GN=UBC PE=1 SV=3 - [UBC_HUMAN] \| \| Structural maintenance of chromosomes protein 6 OS=Homo sapiens GN=SMC6 PE=1 SV=2 - [SMC6_HUMAN] \| \| Putative pre-mRNA-splicing factor ATP-dependent RNA helicase DHX16 OS=Homo sapiens GN=DHX16 PE=1 SV=2 - [DHX16_HUMAN] \| \| ELKS/Rab6-interacting/CAST family member 1 OS=Homo sapiens GN=ERC1 PE=1 SV=1 - [RB6I2_HUMAN] \| \| Serine/threonine-protein kinase PRP4 homolog OS=Homo sapiens GN=PRPF4B PE=1 SV=3 - [PRP4B_HUMAN] \| \| Splicing factor 3B subunit 3 OS=Homo sapiens GN=SF3B3 PE=1 SV=4 - [SF3B3_HUMAN] \| \| Cytoplasmic FMR1-interacting protein 2 OS=Homo sapiens GN=CYFIP2 PE=1 SV=2 - [CYFP2_HUMAN] \| \| Ubiquitin carboxyl-terminal hydrolase 7 OS=Homo sapiens GN=USP7 PE=1 SV=2 - [UBP7_HUMAN] \| \| Superkiller viralicidic activity 2-like 2 OS=Homo sapiens GN=SKIV2L2 PE=1 SV=3 - [SK2L2_HUMAN] \| \| Histone lysine demethylase PHF8 OS=Homo sapiens GN=PHF8 PE=1 SV=3 - [PHF8_HUMAN] \| \| Golgin subfamily A member 2 OS=Homo sapiens GN=GOLGA2 PE=1 SV=3 - [GOGA2_HUMAN] \| \| Poly [ADP-ribose] polymerase 1 OS=Homo sapiens GN=PARP1 PE=1 SV=4 - [PARP1_HUMAN] \| \| Coatomer subunit alpha OS=Homo sapiens GN=COPA PE=1 SV=2 - [COPA_HUMAN] \| \| U2 snRNP-associated SURP motif-containing protein OS=Homo sapiens GN=U2SURP PE=1 SV=2 - [SR140_HUMAN] \| \| Nucleoprotein TPR OS=Homo sapiens GN=TPR PE=1 SV=3 - [TPR_HUMAN] \| \| DNA mismatch repair protein Msh6 OS=Homo sapiens GN=MSH6 PE=1 SV=2 - [MSH6_HUMAN] \| \| Junction-mediating and -regulatory protein OS=Homo sapiens GN=JMY PE=1 SV=2 - [JMY_HUMAN] \| \| Probable global transcription activator SNF2L1 OS=Homo sapiens GN=SMARCA1 PE=1 SV=2 - [SMCA1_HUMAN] \| \| Non-receptor tyrosine-protein kinase TYK2 OS=Homo sapiens GN=TYK2 PE=1 SV=3 - [TYK2_HUMAN] \| \| DNA mismatch repair protein Msh3 OS=Homo sapiens GN=MSH3 PE=1 SV=4 - [MSH3_HUMAN] \| \| Probable ATP-dependent RNA helicase YTHDC2 OS=Homo sapiens GN=YTHDC2 PE=1 SV=2 - [YTDC2_HUMAN] \| \| Poly(ADP-ribose) glycohydrolase OS=Homo sapiens GN=PARG PE=1 SV=1 - [PARG_HUMAN] \| \| Partitioning defective 3 homolog OS=Homo sapiens GN=PARD3 PE=1 SV=2 - [PARD3_HUMAN] \| \| SLIT-ROBO Rho GTPase-activating protein 2 OS=Homo sapiens GN=SRGAP2 PE=1 SV=2 - [FNBP2_HUMAN] \| \| Kelch-like ECH-associated protein 1 OS=Homo sapiens GN=KEAP1 PE=1 SV=2 - [KEAP1_HUMAN] \| \| 5-azacytidine-induced protein 1 OS=Homo sapiens GN=AZI1 PE=1 SV=3 - [AZI1_HUMAN] \| \| SLIT-ROBO Rho GTPase-activating protein 1 OS=Homo sapiens GN=SRGAP1 PE=1 SV=1 - [SRGP1_HUMAN] \| \| ERC protein 2 OS=Homo sapiens GN=ERC2 PE=1 SV=3 - [ERC2_HUMAN] \| \| Ubiquitin carboxyl-terminal hydrolase 25 OS=Homo sapiens GN=USP25 PE=1 SV=4 - [UBP25_HUMAN] \| \| Cohesin subunit SA-2 OS=Homo sapiens GN=STAG2 PE=1 SV=3 - [STAG2_HUMAN] \| \| Scaffold attachment factor B1 OS=Homo sapiens GN=SAFB PE=1 SV=4 - [SAFB1_HUMAN] \| \| Splicing factor 3B subunit 2 OS=Homo sapiens GN=SF3B2 PE=1 SV=2 - [SF3B2_HUMAN] \| \| MORC family CW-type zinc finger protein 2 OS=Homo sapiens GN=MORC2 PE=1 SV=2 - [MORC2_HUMAN] \| \| Actin-binding protein anillin OS=Homo sapiens GN=ANLN PE=1 SV=2 - [ANLN_HUMAN] \| \| WD repeat-containing protein 60 OS=Homo sapiens GN=WDR60 PE=1 SV=3 - [WDR60_HUMAN] \| \| Splicing factor 3B subunit 1 OS=Homo sapiens GN=SF3B1 PE=1 SV=3 - [SF3B1_HUMAN] \| \| Formin-like protein 3 OS=Homo sapiens GN=FMNL3 PE=1 SV=3 - [FMNL3_HUMAN] \| \| E3 ubiquitin-protein ligase BRE1B OS=Homo sapiens GN=RNF40 PE=1 SV=4 - [BRE1B_HUMAN] \| \| Structural maintenance of chromosomes protein 5 OS=Homo sapiens GN=SMC5 PE=1 SV=2 - [SMC5_HUMAN] \| \| CLIP-associating protein 2 OS=Homo sapiens GN=CLASP2 PE=1 SV=2 - [CLAP2_HUMAN] \| \| CLIP-associating protein 1 OS=Homo sapiens GN=CLASP1 PE=1 SV=1 - [CLAP1_HUMAN] \| \| Cytospin-A OS=Homo sapiens GN=SPECC1L PE=1 SV=2 - [CYTSA_HUMAN] \| \| Ankycorbin OS=Homo sapiens GN=RAI14 PE=1 SV=2 - [RAI14_HUMAN] \| \| Cleavage and polyadenylation specificity factor subunit 1 OS=Homo sapiens GN=CPSF1 PE=1 SV=2 - [CPSF1_HUMAN] \| \| Host cell factor 1 OS=Homo sapiens GN=HCFC1 PE=1 SV=2 - [HCFC1_HUMAN] \| \| RNA-binding protein 12B OS=Homo sapiens GN=RBM12B PE=1 SV=2 - [RB12B_HUMAN] \| \| Cytoskeleton-associated protein 5 OS=Homo sapiens GN=CKAP5 PE=1 SV=3 - [CKAP5_HUMAN] \| \| Condensin-2 complex subunit G2 OS=Homo sapiens GN=NCAPG2 PE=1 SV=1 - [CNDG2_HUMAN] \| \| SURP and G-patch domain-containing protein 2 OS=Homo sapiens GN=SUGP2 PE=1 SV=2 - [SUGP2_HUMAN] \| \| RNA-binding protein 26 OS=Homo sapiens GN=RBM26 PE=1 SV=3 - [RBM26_HUMAN] \| \| PAB-dependent poly(A)-specific ribonuclease subunit 2 OS=Homo sapiens GN=PAN2 PE=1 SV=3 - [PAN2_HUMAN] \| \| Ataxin-2-like protein OS=Homo sapiens GN=ATXN2L PE=1 SV=2 - [ATX2L_HUMAN] \| \| Trafficking protein particle complex subunit 9 OS=Homo sapiens GN=TRAPPC9 PE=1 SV=2 - [TPPC9_HUMAN] \| \| E3 SUMO-protein ligase RanBP2 OS=Homo sapiens GN=RANBP2 PE=1 SV=2 - [RBP2_HUMAN] \| \| Dedicator of cytokinesis protein 7 OS=Homo sapiens GN=DOCK7 PE=1 SV=4 - [DOCK7_HUMAN] \| \| MAP7 domain-containing protein 1 OS=Homo sapiens GN=MAP7D1 PE=1 SV=1 - [MA7D1_HUMAN] \| \| DNA-directed RNA polymerase III subunit RPC2 OS=Homo sapiens GN=POLR3B PE=1 SV=2 - [RPC2_HUMAN] \| \| Tyrosine-protein kinase JAK1 OS=Homo sapiens GN=JAK1 PE=1 SV=2 - [JAK1_HUMAN] \| \| Squamous cell carcinoma antigen recognized by T-cells 3 OS=Homo sapiens GN=SART3 PE=1 SV=1 - [SART3_HUMAN] \| \| Transcription initiation factor TFIID subunit 2 OS=Homo sapiens GN=TAF2 PE=1 SV=3 - [TAF2_HUMAN] \| \| La-related protein 1 OS=Homo sapiens GN=LARP1 PE=1 SV=2 - [LARP1_HUMAN] \| \| WASH complex subunit 7 OS=Homo sapiens GN=KIAA1033 PE=1 SV=2 - [WASH7_HUMAN] \| \| Exportin-5 OS=Homo sapiens GN=XPO5 PE=1 SV=1 - [XPO5_HUMAN] \| \| Ubiquitin-associated protein 2-like OS=Homo sapiens GN=UBAP2L PE=1 SV=2 - [UBP2L_HUMAN] \| \| ATP-dependent RNA helicase DHX8 OS=Homo sapiens GN=DHX8 PE=1 SV=1 - [DHX8_HUMAN] \| \| Mitotic checkpoint serine/threonine-protein kinase BUB1 beta OS=Homo sapiens GN=BUB1B PE=1 SV=3 - [BUB1B_HUMAN] \| \| Plakophilin-4 OS=Homo sapiens GN=PKP4 PE=1 SV=2 - [PKP4_HUMAN] \| \| Extended synaptotagmin-1 OS=Homo sapiens GN=ESYT1 PE=1 SV=1 - [ESYT1_HUMAN] \| \| AP-3 complex subunit beta-1 OS=Homo sapiens GN=AP3B1 PE=1 SV=3 - [AP3B1_HUMAN] \| \| Kinesin-like protein KIF11 OS=Homo sapiens GN=KIF11 PE=1 SV=2 - [KIF11_HUMAN] \| \| Probable ATP-dependent RNA helicase DHX34 OS=Homo sapiens GN=DHX34 PE=2 SV=2 - [DHX34_HUMAN] \| \| Elongator complex protein 1 OS=Homo sapiens GN=IKBKAP PE=1 SV=3 - [ELP1_HUMAN] \| \| Ubiquitin carboxyl-terminal hydrolase 37 OS=Homo sapiens GN=USP37 PE=1 SV=2 - [UBP37_HUMAN] \| \| Nuclear pore complex protein Nup155 OS=Homo sapiens GN=NUP155 PE=1 SV=1 - [NU155_HUMAN] \| \| Ankyrin repeat and BTB/POZ domain-containing protein BTBD11 OS=Homo sapiens GN=BTBD11 PE=2 SV=3 - [BTBDB_HUMAN] \| \| Uncharacterized protein KIAA0528 OS=Homo sapiens GN=KIAA0528 PE=1 SV=1 - [K0528_HUMAN] \| \| Cytospin-B OS=Homo sapiens GN=SPECC1 PE=1 SV=1 - [CYTSB_HUMAN] \| \| Unconventional myosin-VI OS=Homo sapiens GN=MYO6 PE=1 SV=4 - [MYO6_HUMAN] \| \| Rho GTPase-activating protein 39 OS=Homo sapiens GN=ARHGAP39 PE=1 SV=2 - [RHG39_HUMAN] \| \| WD repeat and HMG-box DNA-binding protein 1 OS=Homo sapiens GN=WDHD1 PE=1 SV=1 - [WDHD1_HUMAN] \| \| UPF0614 protein C14orf102 OS=Homo sapiens GN=C14orf102 PE=1 SV=3 - [CN102_HUMAN] \| \| Eukaryotic translation initiation factor 3 subunit C OS=Homo sapiens GN=EIF3C PE=1 SV=1 - [EIF3C_HUMAN] \| \| Protein transport protein Sec24C OS=Homo sapiens GN=SEC24C PE=1 SV=3 - [SC24C_HUMAN] \| \| Protein FAM83H OS=Homo sapiens GN=FAM83H PE=1 SV=3 - [FA83H_HUMAN] \| \| Myosin-10 OS=Homo sapiens GN=MYH10 PE=1 SV=3 - [MYH10_HUMAN] \| \| Histone deacetylase complex subunit SAP130 OS=Homo sapiens GN=SAP130 PE=1 SV=1 - [SP130_HUMAN] \| \| Pre-mRNA-processing factor 40 homolog A OS=Homo sapiens GN=PRPF40A PE=1 SV=2 - [PR40A_HUMAN] \| \| Coiled-coil and C2 domain-containing protein 1A OS=Homo sapiens GN=CC2D1A PE=1 SV=1 - [C2D1A_HUMAN] \| \| Microtubule-associated protein 1S OS=Homo sapiens GN=MAP1S PE=1 SV=2 - [MAP1S_HUMAN] \| \| Transcription elongation factor SPT5 OS=Homo sapiens GN=SUPT5H PE=1 SV=1 - [SPT5H_HUMAN] \| \| Spectrin beta chain, brain 1 OS=Homo sapiens GN=SPTBN1 PE=1 SV=2 - [SPTB2_HUMAN] \| \| DIS3-like exonuclease 1 OS=Homo sapiens GN=DIS3L PE=1 SV=2 - [DI3L1_HUMAN] \| \| Nodal modulator 3 OS=Homo sapiens GN=NOMO3 PE=2 SV=2 - [NOMO3_HUMAN] \| \| Vinculin OS=Homo sapiens GN=VCL PE=1 SV=4 - [VINC_HUMAN] \| \| Leucine zipper protein 1 OS=Homo sapiens GN=LUZP1 PE=1 SV=2 - [LUZP1_HUMAN] \| \| DNA-dependent protein kinase catalytic subunit OS=Homo sapiens GN=PRKDC PE=1 SV=3 - [PRKDC_HUMAN] \| \|  \| |
| --- | --- | --- | --- | --- | --- | --- | --- | --- | --- | --- | --- | --- | --- | --- | --- | --- | --- | --- | --- | --- | --- | --- | --- | --- | --- | --- | --- | --- | --- | --- | --- | --- | --- | --- | --- | --- | --- | --- | --- | --- | --- | --- | --- | --- | --- | --- | --- | --- | --- | --- | --- | --- | --- | --- | --- | --- | --- | --- | --- | --- | --- | --- | --- | --- | --- | --- | --- | --- | --- | --- | --- | --- | --- | --- | --- | --- | --- | --- | --- | --- | --- | --- | --- | --- | --- | --- | --- | --- | --- | --- | --- | --- | --- | --- | --- | --- | --- | --- | --- | --- | --- | --- | --- | --- | --- | --- | --- | --- | --- | --- | --- | --- | --- | --- | --- | --- | --- | --- | --- | --- | --- | --- | --- | --- | --- | --- | --- | --- | --- | --- | --- | --- | --- | --- | --- | --- | --- | --- | --- | --- | --- | --- | --- | --- | --- | --- | --- | --- | --- | --- | --- | --- | --- | --- | --- | --- | --- | --- | --- | --- | --- | --- | --- | --- | --- | --- | --- | --- | --- | --- | --- | --- | --- | --- | --- | --- | --- | --- | --- | --- | --- | --- | --- | --- | --- | --- | --- | --- | --- | --- | --- | --- | --- | --- | --- | --- | --- | --- | --- | --- | --- | --- | --- | --- | --- | --- | --- | --- | --- | --- | --- | --- | --- | --- | --- | --- | --- | --- | --- | --- | --- | --- | --- | --- | --- | --- | --- | --- | --- | --- | --- | --- | --- | --- | --- | --- | --- | --- | --- | --- | --- | --- | --- | --- | --- | --- | --- | --- | --- | --- | --- | --- | --- | --- | --- | --- | --- | --- | --- | --- | --- | --- | --- | --- | --- | --- | --- | --- | --- | --- | --- | --- | --- | --- | --- | --- | --- | --- | --- | --- | --- | --- | --- | --- | --- | --- | --- | --- | --- | --- | --- | --- | --- | --- | --- | --- | --- | --- | --- | --- | --- | --- | --- | --- | --- | --- | --- | --- | --- | --- | --- | --- | --- | --- | --- | --- | --- | --- | --- | --- | --- | --- | --- | --- | --- | --- | --- | --- | --- | --- | --- | --- | --- | --- | --- | --- | --- | --- | --- | --- | --- | --- | --- | --- | --- | --- | --- | --- | --- | --- | --- | --- | --- | --- | --- | --- | --- | --- | --- | --- | --- | --- | --- | --- | --- | --- | --- | --- | --- | --- | --- | --- | --- | --- | --- | --- | --- | --- | --- | --- | --- | --- | --- | --- | --- | --- | --- | --- | --- | --- | --- | --- | --- | --- | --- | --- | --- | --- | --- | --- | --- | --- | --- | --- | --- | --- | --- | --- | --- | --- | --- | --- | --- | --- | --- | --- | --- | --- | --- | --- | --- | --- | --- | --- | --- | --- | --- | --- | --- | --- | --- | --- | --- | --- | --- | --- | --- | --- | --- | --- | --- | --- | --- | --- | --- | --- | --- | --- | --- | --- | --- | --- | --- | --- | --- | --- | --- | --- | --- | --- | --- | --- | --- | --- | --- | --- | --- | --- | --- | --- | --- | --- | --- | --- | --- | --- | --- | --- | --- | --- | --- | --- | --- | --- | --- | --- | --- | --- | --- | --- | --- | --- | --- | --- | --- | --- | --- | --- | --- | --- | --- | --- | --- | --- | --- | --- | --- | --- | --- | --- | --- | --- | --- | --- | --- | --- | --- | --- | --- | --- | --- | --- | --- | --- | --- | --- | --- | --- | --- | --- | --- | --- | --- | --- | --- | --- | --- | --- | --- | --- | --- | --- | --- | --- | --- | --- | --- | --- | --- | --- | --- | --- | --- | --- | --- | --- | --- | --- | --- | --- | --- | --- | --- | --- | --- | --- | --- | --- | --- | --- | --- | --- | --- | --- | --- | --- | --- | --- | --- | --- | --- | --- | --- | --- | --- | --- | --- | --- | --- | --- | --- | --- | --- | --- | --- | --- | --- | --- | --- | --- | --- | --- | --- | --- | --- | --- | --- | --- | --- | --- | --- | --- | --- | --- | --- | --- | --- | --- | --- | --- | --- | --- | --- | --- | --- | --- | --- | --- | --- | --- | --- | --- | --- | --- | --- | --- | --- | --- | --- | --- | --- | --- | --- | --- | --- | --- | --- | --- | --- | --- | --- | --- | --- | --- | --- | --- | --- | --- | --- | --- | --- | --- | --- | --- | --- | --- | --- | --- | --- | --- | --- | --- | --- | --- | --- | --- | --- | --- | --- | --- | --- | --- | --- | --- | --- | --- | --- | --- | --- | --- | --- | --- | --- | --- | --- | --- | --- | --- | --- | --- | --- | --- | --- | --- | --- | --- | --- | --- | --- | --- | --- | --- | --- | --- | --- | --- | --- | --- | --- | --- | --- | --- | --- | --- | --- | --- | --- | --- | --- | --- | --- | --- | --- | --- | --- | --- | --- | --- | --- | --- | --- | --- | --- | --- | --- | --- | --- | --- | --- | --- | --- | --- | --- | --- | --- | --- | --- | --- | --- | --- | --- | --- | --- | --- | --- | --- | --- | --- | --- | --- | --- | --- | --- | --- | --- | --- | --- | --- | --- | --- | --- | --- | --- | --- | --- | --- | --- | --- | --- | --- | --- | --- | --- | --- | --- | --- | --- | --- | --- | --- | --- | --- | --- | --- | --- | --- | --- | --- | --- | --- | --- | --- | --- | --- | --- | --- | --- | --- | --- | --- | --- | --- | --- | --- | --- | --- | --- | --- | --- | --- | --- | --- | --- | --- | --- | --- | --- | --- | --- | --- | --- | --- | --- | --- | --- | --- | --- | --- | --- | --- | --- | --- | --- | --- | --- | --- | --- | --- | --- | --- | --- | --- | --- | --- | --- | --- | --- | --- | --- | --- | --- | --- | --- | --- | --- | --- | --- | --- | --- | --- | --- | --- | --- | --- | --- | --- | --- | --- | --- | --- | --- | --- | --- | --- | --- | --- | --- | --- | --- | --- | --- | --- | --- | --- | --- | --- | --- | --- | --- | --- | --- | --- | --- | --- | --- | --- | --- | --- | --- | --- | --- | --- | --- | --- | --- | --- | --- | --- | --- | --- | --- | --- | --- | --- | --- | --- | --- | --- | --- | --- | --- | --- | --- | --- | --- | --- | --- | --- | --- | --- | --- | --- | --- | --- | --- | --- | --- | --- | --- | --- | --- | --- | --- | --- | --- | --- | --- | --- | --- | --- | --- | --- | --- | --- | --- | --- | --- | --- | --- | --- | --- | --- | --- | --- | --- | --- | --- | --- | --- | --- | --- | --- | --- | --- | --- | --- | --- | --- | --- | --- | --- | --- | --- | --- | --- | --- | --- | --- | --- | --- | --- | --- | --- | --- | --- | --- | --- | --- | --- | --- | --- | --- | --- | --- | --- | --- | --- | --- | --- | --- | --- | --- | --- | --- | --- | --- | --- | --- | --- | --- | --- | --- | --- | --- | --- | --- | --- | --- | --- | --- | --- | --- | --- | --- | --- | --- | --- | --- | --- | --- | --- | --- | --- | --- | --- | --- | --- | --- | --- | --- | --- | --- | --- | --- | --- | --- | --- | --- | --- | --- | --- | --- | --- | --- | --- | --- | --- | --- | --- | --- | --- | --- | --- | --- | --- | --- | --- | --- | --- | --- | --- | --- | --- | --- | --- | --- |
